# Drug delivery, biodistribution and anti-EGFR activity: theragnostic nanoparticles for simultaneous *in vivo* delivery of tyrosine kinase inhibitors and kinase activity biosensors†

**DOI:** 10.1039/d1nr02770k

**Published:** 2021-11-03

**Authors:** Robin Bofinger, Gregory Weitsman, Rachel Evans, Matthias Glaser, Kerstin Sander, Helen Allan, Daniel Hochhauser, Tammy L. Kalber, Erik Årstad, Helen C. Hailes, Tony Ng, Alethea B. Tabor

**Affiliations:** Department of Chemistry, University College London 20 Gordon Street London WC1H 0AJ UK a.b.tabor@ucl.ac.uk; School of Cancer and Pharmaceutical Sciences, King's College London London SE1 1UL UK tony.ng@kcl.ac.uk; UCL Cancer Institute, Paul O'Gorman Building, University College London London WC1E 6DD UK; Centre for Radiopharmaceutical Chemistry Kathleen Lonsdale Building 5 Gower Place London WC1E 6BS UK; Centre for Advanced Biomedical Imaging, Paul O'Gorman Building, University College London London WC1E 6DD UK

## Abstract

*In vivo* delivery of small molecule therapeutics to cancer cells, assessment of the selectivity of administration, and measuring the efficacity of the drug in question at the molecule level, are important ongoing challenges in developing new classes of cancer chemotherapeutics. One approach that has the potential to provide targeted delivery, tracking of biodistribution and readout of efficacy, is to use multimodal theragnostic nanoparticles to deliver the small molecule therapeutic. In this paper, we report the development of targeted theragnostic lipid/peptide/DNA lipopolyplexes. These simultaneously deliver an inhibitor of the EGFR tyrosine kinase, and plasmid DNA coding for a Crk-based biosensor, Picchu-X, which when expressed in the target cells can be used to quantify the inhibition of EGFR *in vivo* in a mouse colorectal cancer xenograft model. Reversible bioconjugation of a known analogue of the tyrosine kinase inhibitor Mo-IPQA to a cationic peptide, and co-formulation with peptides containing both EGFR-binding and cationic sequences, allowed for good levels of inhibitor encapsulation with targeted delivery to LIM1215 colon cancer cells. Furthermore, high levels of expression of the Picchu-X biosensor in the LIM1215 cells *in vivo* allowed us to demonstrate, using fluorescence lifetime microscopy (FLIM)-based biosensing, that EGFR activity can be successfully suppressed by the tyrosine kinase inhibitor, released from the lipopolyplexes. Finally, we measured the biodistribution of lipopolyplexes containing ^125^I-labelled inhibitors and were able to demonstrate that the lipopolyplexes gave significantly higher drug delivery to the tumors compared with free drug.

## Abstract 2

We report the development of targeted theragnostic lipid/peptide/DNA lipopolyplexes for delivery of both a tyrosine kinase inhibitor, and plasmid DNA coding for a biosensor. These are used to quantify EGFR inhibition in cancer cell lines *in vivo*.

## Graphical abstract

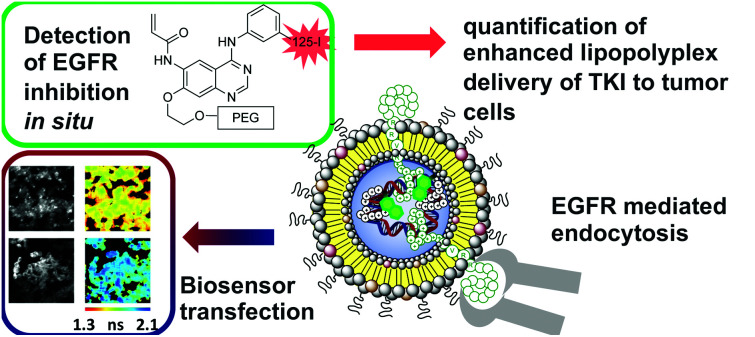

## Introduction

Current challenges in next-generation medicine have stimulated the rapid development of theragnostic agents. These are of increasing importance for diseases such as cancer, where no two patients will have exactly the same biomarkers and oncogenic mutations, and where most currently available therapeutic agents have limited target selectivity, poor localization, and undesirable side-effects. Theragnostic agents can be based on small molecules conjugated to a targeting moiety, such as an antibody; on engineered mammalian cells; and on various types of nanoparticles (*e.g.* iron oxide, gold, polymeric or liposomal).^1^ Whilst all of these have limitations,^2^ liposome-based nanoparticles have several key advantages. These include the ability to design multimodal nanoparticles with several functionalities contained in, or attached to, a single liposome: rapid cellular uptake; a wide range of cellular compatibilities and low toxicity; and long circulating half-life combined with eventual biodegradability.^3^

Many theragnostic agents have now been developed which are capable of diagnosing, through imaging, the presence of cancerous cells and then delivering a precisely targeted therapeutic intervention.^4^ Whilst these approaches can be used to measure biodistribution and accumulation of small molecule drugs at the tumor, none of the theragnostic agents reported to date can quantify the effectiveness of the therapeutic at the molecular level *in vivo*. This is particularly important in targeted cancer therapy. Although diagnostic assays are available to determine protein expression status for some types of cancer, for many tumor types there are either no validated assays, or the expression status of the protein cannot in practice be correlated with the clinical response to the drug.^5^ The development of theragnostic nanoparticles which would improve drug delivery, whilst also allowing both the biodistribution *and* response to the drug to be measured, would be a major step towards the development of more effective cancer therapies, tailored towards the driving molecular phenotype of an individual tumor.

Epidermal growth factor receptor (EGFR) is found to be overexpressed and/or constitutively activated in a variety of tumors including breast, lung and colon^6^ and is a validated target for drug development. The EGFR family^7^ consists of four members (EGFR, HER2, HER3 and HER4) with more than 10 different ligands^8^ able to activate downstream signaling, leading to growth proliferation and inhibition of apoptosis, angiogenesis and metastasis. Several therapeutic strategies targeting EGFR have been devised. In particular, small molecule inhibitors of the EGFR intracellular tyrosine kinase activity (TKI), and antibodies blocking ligand interaction have been developed and tested in preclinical and clinical studies.^9,10^ Unfortunately, the factors that predict clinical response to anti-EGFR agents remain unclear, and the success rate for these reagents in the clinic is low. Expression of EGFR does not correlate with efficacy of the EGFR antibody cetuximab^11^ and the use of the antibody is limited by toxicity.^12^ In recent clinical studies of EGFR-expressing breast^13^ and colorectal^14^ cancer patients, response rates to EGFR-targeted therapies were low and varied between cancer types, with the majority of patients eventually developing resistance to these reagents. It is clear that in order for these strategies to be successful, much more research is needed to understand how EGFR inhibitors and antibodies mechanistically function *in vivo* at both the cellular and molecular level.^15^

A strategy for overcoming the poor pharmacokinetic profiles and low target selectivity of anticancer drugs is to use liposomes, liposome-based nanoparticles and polymersomes as delivery vehicles.^16^ The polyethylene glycol (PEG)-shielded liposomal doxorubicin (DOXIL®) is clinically approved for the treatment of ovarian and breast cancer, with significantly longer circulation times and lower cardiotoxicity compared to the free drug, and several other liposomal drug formulations are in Phase II trials for other cancers.^17^ At the preclinical level, radiolabeling^18^ of liposome-encapsulated dasatinib with ^18^F and liposome-encapsulated Mo-IPQA labeled^19^ with ^124^I have enabled quantification, demonstrating some of the highest tumor uptakes *in vivo* of TKI so far observed. Non-targeted liposomal drug formulations were believed to rely on the passive accumulation of these nanoparticles (100–200 nm) in tumors through the Enhanced Permeability and Retention (EPR) effect. However, many clinical studies have failed to show either increased nanoparticle accumulation in tumors or increased efficacy, and it is now clear that this effect is, at best, heterogeneous in humans.^20,21^ Ligand-mediated cell-selective targeting offers additional advantages in terms of target cell specificity and cellular uptake. For example, immunoliposomes have been prepared using anti-EGFR antibodies as targeting moieties and were used to deliver drugs such as 5-fluorouracil,^22^ cisplatin^23^ and doxorubicin^24^ to cancer cells *in vivo*, with moderate to significant improvements in cytotoxicity and cell selectivity compared to non-targeted liposomes. Likewise, polymersomes encapsulating plitidepsin and targeted with an anti-EGFR antibody showed greatly increased cytotoxicity and cellular uptake in colorectal cancer cell lines compared to an untargeted polymersome.^25^ Liposomes targeted with EGFR-binding peptides have also been used to deliver doxorubicin^26^ and cisplatin^27^ effectively to cancer cells. Finally, lipoplexes targeted with anti-EGFR antibodies have been formulated to deliver siRNA to non-small cell lung cancer cell lines^28^ and into EGFR-overexpressing hepatocellular carcinoma.^29^ Surface modification was necessary for specific uptake of the siRNA into the cells and resulted in enhanced tumor accumulation when compared to untargeted lipoplexes.

We have previously reported^30^ the development of EGFR-targeted lipopolyplexes for the delivery of pDNA encoding an EGFR biosensor.^31^ This allowed us to monitor EGFR activity in a breast cancer model *in vivo*, before and after separate treatment with a TKI, Mo-IPQA.^30^ As well as successfully demonstrating the inhibition of tyrosine phosphorylation by EGFR *in vivo*, and showing a significant degree of intratumoral heterogeneity in EGFR activity, our results showed that small molecule TKIs have a very poor uptake in cancer cells, and that the uptake is unselective. In this paper, we report the development of the first targeted, lipopolyplex-based theragnostic nanoparticle that improves drug delivery to cancer cells, allows quantification of the biodistribution, and also provides a readout of TKI mediated enzyme inhibition. As the reasons for the heterogeneity of response of colorectal cancer cells to EGFR-targeted therapies, and the rapid emergence of chemotherapy-resistant strains, are still poorly understood^15^ these nanoparticles have been used for preliminary investigation of biodistribution and uptake in a murine xenograft colorectal cancer model.

## Results and discussion

### Design of lipopolyplex-based theragnostic nanoparticles

In our previous work^32–38^ we have developed lipopolyplexes formulated from a ternary mixture of lipids, peptides and DNA for the targeted delivery of pDNA to tumor cells. The peptide component is a trifunctional sequence comprising: a linear K_16_ domain to condense pDNA; a cell-targeting sequence that is displayed at the surface of the nanoparticle; and a linker sequence, RVRR, that is a substrate for the endosomal enzyme furin. Mixtures of cationic and neutral lipids are used and are co-formulated with the neutral lipid dioleoylphosphatidylethanolamine (DOPE). The cationic lipids include both DOTMA^39^ and the analogue DODEG4^32^ which has a short ethylene glycol oligomer headgroup. These give shielded nanocomplexes that have better stability within the systemic circulation but do not impede cellular uptake.

The targeting peptide sequence at the surface of these lipopolyplexes binds to the cell of interest and mediates internalisation *via* receptor-mediated endocytosis: cleavage of the peptide at the RVRR sequence allows partial disassembly of the nanoparticle after internalisation,^33^ and the neutral lipid DOPE promotes fusion with the endosomal membrane and mediates escape of the pDNA/peptide complex from the endosome.^40^ These nanoparticles are able to transfect tumor cells *in vivo* with transfection efficiencies comparable to viral methods,^34^ and lipopolyplexes generally have lower cytotoxicity than commercially available transfection reagents such as Lipofectamine2000.^37,38,41,42^ We have previously carried out biophysical characterisation of these lipopolyplexes by a variety of techniques including TEM,^37^ freeze-fracture EM^35^ and cryo-EM.^36^ These EM studies suggest that these lipopolyplexes form spherical particles with a dense internal core and an irregular outer layer.

To report the levels of EGFR activity in cells using these ternary lipopolyplexes, we used a genetically encoded CrkII-based biosensor (Picchu-X) which undergoes conformational changes upon phosphorylation of tyrosine-221 by EGFR.^31,43^ These changes are quantified by fluorescence resonance energy transfer (FRET) as monitored by fluorescence lifetime imaging microscopy (FLIM). We transfected this biosensor into EGFR-positive tumor cells using targeted ternary liposome-base nanoparticles bearing EGFR-binding peptides at the surface, and measured the response of these tumor cells to a TKI, Mo-IPQA, administered separately.^30^

**Fig. 1:**
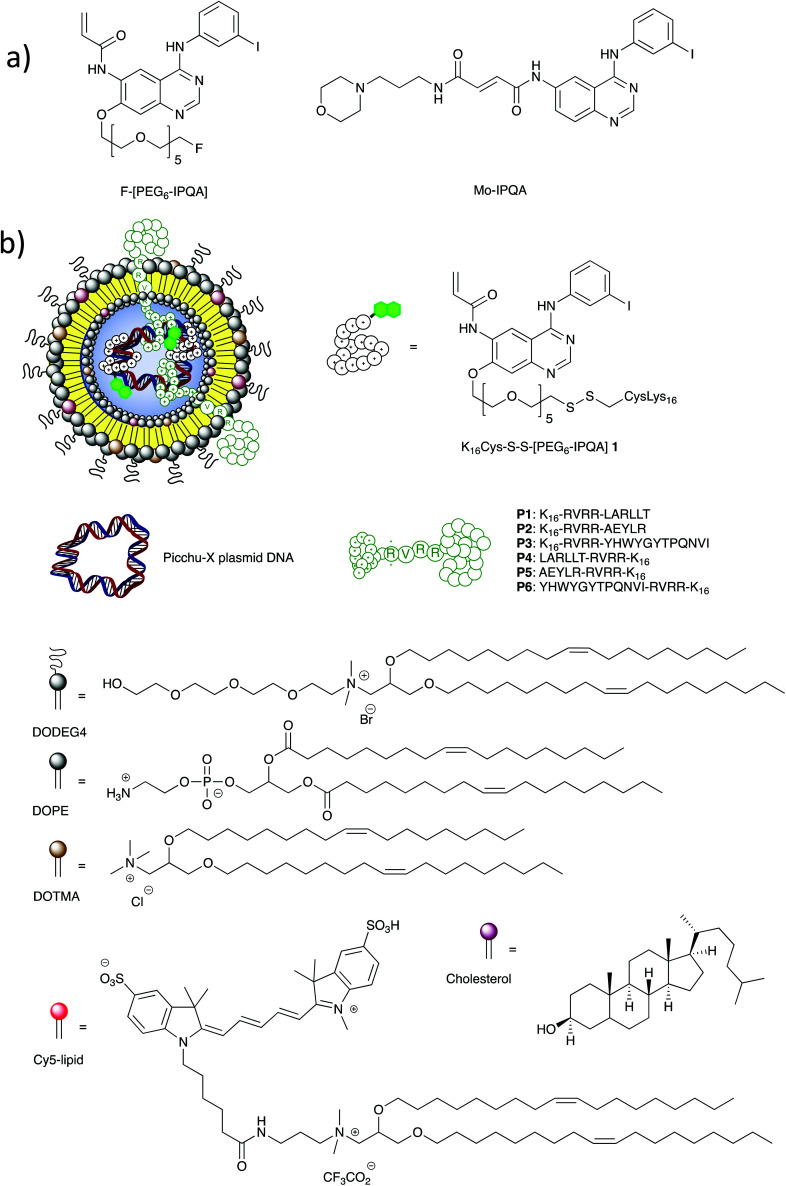
(a) Structures of the previously reported ^18^F PET radiotracer F-[PEG_6_-IPQA]^52^ and the EGFR TKI Mo-IPQA;^51^ (b) peptide, lipid and plasmid DNA components of the lipopolyplex nanoparticles used in this work.

In order to achieve our goals of selectively delivering a TKI to colorectal cancer cells, imaging the inhibition of EGFR within the tumor and evaluating the biodistribution of the TKI, we designed enhanced multifunctional lipopolyplex-based nanoparticles (Fig. 1). These lipopolyplex formulations had to include: mixtures of lipids that would enhance the particle stability and provide steric shielding; peptide targeting sequences that would promote uptake from colorectal cancer cells; and a cationic peptide–TKI bioconjugate that would enable incorporation of high levels of both the TKI and the pDNA within the lipopolyplex. Additionally, we needed to develop synthetic strategies to ensure that the cationic peptide–TKI bioconjugate could be radiolabelled with ^125^I, thus allowing the biodistribution of both the TKI and the lipopolyplex to be assessed.

With this next generation of theragnostic lipopolyplexes, in addition to DOPE, DOTMA and DODEG-4 we included cholesterol to further increase bilayer stability,^44^ and Cy-5-DOTMA^30^ to enable the uptake of the nanoparticles to the tumor cells to be easily monitored. In order to target the nanoparticles efficiently to cell lines over-expressing EGFR, we synthesized trifunctional peptides with DNA-binding linear K_16_, RVRR and cell-targeting sequences, as before. We have evaluated three peptide sequences for their ability to target colorectal cancer cells. Peptides **P1** and **P4** use the LARLLT (D4) sequence,^45^**P2** and **P5** the AEYLR^46^ and **P3** and **P6** the YHWYGYTPQNVI (GE11) sequence.^47,48^ These sequences had been previously reported to bind tightly to EGFR and, when displayed at the surface of nanoparticles, mediate uptake to EGFR+ tumor cells. Indeed, we have previously demonstrated that lipopolyplexes formulated with **P1** effectively deliver pDNA encoding for a biosensor to HCC1954 and MDA-MB-231 breast cancer cell lines *in vivo*,^30^ and that lipopolyplexes formulated with **P1**, **P2** or **P3** effectively transfect HCC1954 cells *in vitro*.^49^

The major challenge to the design of these self-assembling nanoparticles was the co-encapsulation of the small molecule TKI with pDNA. Whilst several groups have developed multifunctional nanoparticles for combination therapy with small molecules and nucleic acids,^50–54^ most of these approaches have focused on the co-delivery of siRNA, and many of the reported approaches are based on dendrimers, which have associated toxicities and result in the formation of heterogeneous mixtures.^55^ In some cases where liposomal co-formulation of both nucleic acid and small molecule was attempted, aggregation of the liposome and leakage of the drug was seen.^56^ In order to ensure that the TKI was successfully encapsulated in the self-assembling nanoparticle, and was retained within the nanoparticle after formulation, we sought to reversibly bioconjugate the TKI to one of the components of the nanoparticle. We therefore designed a cationic peptide–TKI bioconjugate **1** (Fig. 1) with a disulfide linkage between the two moieties. We based our design on the known EGFR TKI, F-[PEG_6_-IPQA], a modified form of Mo-IPQA^19,51^ that had been developed previously as an ^18^F PET radiotracer (Fig. 1a).^57^ As the PEG moiety of this TKI is predicted to protrude from the active site of the enzyme^58^ we reasoned that minimal modification to give a thiol-terminated PEG_6_ would not adversely affect the potency or selectivity of this inhibitor, but would allow bioconjugation to K_16_Cys to give K_16_Cys-S-S-[PEG_6_-IPQA] **1**. It was also envisaged that the K_16_ cationic sequence of this bioconjugate would bind to pDNA with the same efficiency as the trifunctional K_16_-RVRR-[targeting sequence] or [targeting sequence]-RVRR-K_16_ peptides **P1–P6**, and that we could therefore prepare lipopolyplexes using a mixture of **1** and peptides **P1–P6**. After internalization of the lipopolyplex, we predicted that the disulfide linkage would be cleaved in the reducing environment of the endosome,^59,60^ releasing PEG_6_-IPQA into the cytoplasm.

### Synthesis of K_16_Cys-S-S-[PEG_6_-IPQA] **1** and MeO-[PEG_6_-IPQA] **9**

**Scheme 1:**
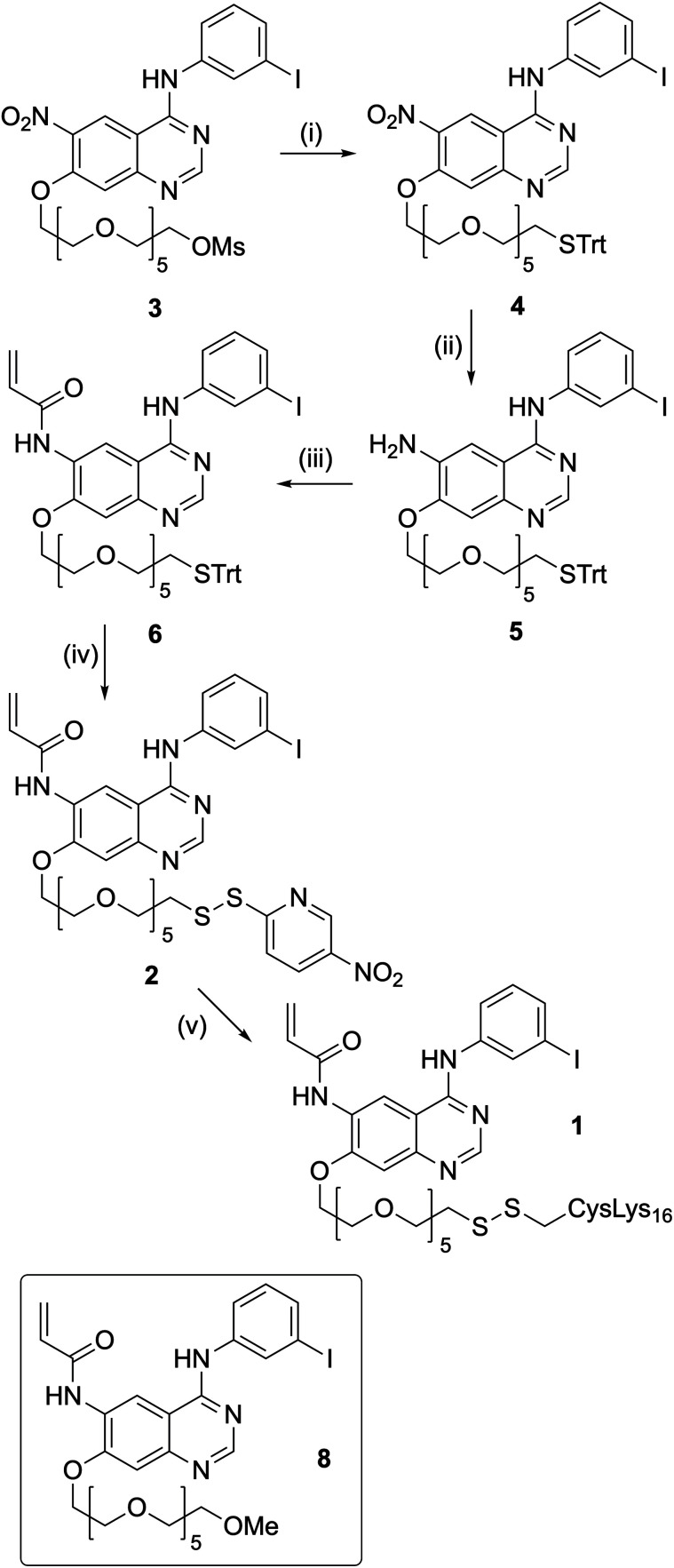
Reagents and conditions: (i) Ph_3_CSH, NaH, THF, rt, 2 h (82%); (ii) SnCl_2_·H_2_O, THF, reflux, 3 h (61%); (iii) isobutyl chloroformate, acrylic acid, Et_3_N, −40 °C, 35 min (57%); (iv) DTNP, CH_2_Cl_2_, CF_3_COOH, rt, 2 h (48%); (v) CysLys_16_**7**, MeOH, rt, 1 h (59%).

In order to prepare K_16_Cys-S-S-[PEG_6_-IPQA] **1**, we required the key intermediate NPys-S-PEG_6_-IPQA **2**, with the SH group activated as the NPys derivative^61^ for coupling to the peptide (Scheme 1). The previously reported 17-((4-((3-iodophenyl)amino)-6-nitroquinazolin-7-yl)oxy)-3,6,9,12,15-pentaoxaheptadecyl methanesulfonate (**3**) was synthesized in 52% yield from 7-fluoro-6-nitro-3*H*-quinazolin-4-one in 3 steps using modified literature procedures (see ESI† for details).

This was subsequently reacted with the sodium salt of triphenylmethanethiol to yield the trityl protected thiol compound **4**. Reduction of the nitro group was carried out using tin(ii)chloride which gave the desired amino intermediate **5** in 61% yield. Using a previously reported procedure^58^ compound **5** was reacted with acryloyl isobutyl carbonate, prepared *in situ* from acrylic acid and isobutyl chloroformate, to afford compound **6** in 57% yield. At this point, the trityl protecting group was replaced by NPys in a one pot reaction under acidic conditions using 2,2′-dithiobis(5-nitropyridine)^62^ to afford **2** in 48% yield. In order to prevent competing Michael reaction between the thiol group and the unsaturated amide, it was necessary to maintain the thiol protection throughout the synthetic sequence, and to carry out the replacement of Trt by NPys at acidic pH. Finally, reaction^63^ with the cationic peptide CysLys_16_**7** in MeOH followed by HPLC gave **1** in 59% yield.

### *In vitro* optimization of nanoparticle formulation

To test the ability of lipopolyplexes to deliver pDNA and inhibitor we used LIM1215 colon cancer cells. These are known to have a high level of EGFR expression and response to the TKI inhibitor,^64,65^ and we had previously demonstrated that a reliable xenograft model could be established.^30^ As a control, to check the response of the colorectal cancer cell lines to this class of TKI, we prepared an analogue of **1**, with the disulfide-linked peptide replaced with an –OMe group. TKI analogue MeO-[PEG_6_-IPQA] **8** was synthesized from intermediate **3***via* a similar route to that of **1** (ESI†) and demonstrated to act as a potent inhibitor of EGFR activation in SW48 cells (Fig. S1†).

Initially, the sequence of the trifunctional peptides was investigated to see whether placing the targeting sequence at the N or the C terminal had any effect on lipopolyplex uptake and expression in colorectal cancer cells. Preliminary experiments (ESI, Fig. S2†) suggested that the expression levels were lower for all peptides with the AEYLR sequence (**P2**, **P5**), and higher for LARLLT (**P1**, **P4**) and GE11 (**P3**, **P6**) in DLD1 colorectal cancer cells. In this cell line, the C-terminal LARLLT (**P1**) and N-terminal GE11 (**P3**) peptides yielded the highest expression levels. The best performing formulation (CLA1, using the **P1** sequence) was tested in DLD1 and LIM1215 cells (Fig. S3†). Expression levels of Picchu-X biosensor were far higher in LIM1215 cells, which were selected for further optimisation of the lipopolyplex formulations. Subsequently, the impact of plasmid DNA concentration and targeting peptide (**P1–P6**) on the transfection efficiency in LIM1215 cells were assessed (Fig. S4 and S5†). Although formulations with the C-terminal LARLLT peptide (**P1**) still showed the best transfection efficiency, formulations with N-terminal AEYLR (**P5**) and N-terminal GE11 (**P6**) also performed well in this cell line. In order to optimize the receptor binding and transfection properties of these lipopolyplexes, we then investigated formulations using different targeting peptides **P1**, **P5**, **P6** and differing lipid compositions (Table 1), but initially without K_16_Cys-S-S-[PEG_6_-IPQA] **1**. While we observed that all lipopolyplex formulations were equally internalized by the target cells, changes in the lipid composition and in the concentrations of bifunctional peptides **P1**, **P5**, **P6**, had an impact on the transfection of the Picchu biosensor.

**Table 1:** Lipopolyplex formulations. CLA lipopolyplexes are formed from C-terminal LARLLT bifunctional peptide **P1** and varying amounts of cholesterol. NAE lipopolyplexes are formed from N-terminal AEYLR bifunctional peptide **P5** and varying amounts of cholesterol. NGE lipopolyplexes are formed from N-terminal (GE11) bifunctional peptide **P6** and varying amounts of cholesterol (Fig. 2a). The F1LA and F2LA formulations are based on the CLA1 and CLA2 lipopolyplexes, but with the addition of the cationic peptide–EGFR inhibitor bioconjugate K_16_Cys-S-S-[PEG_6_-IPQA] **1** at varying concentrations (Fig. 2b)

	DODEG4	DOPE	DOTMA	Cholesterol	Cy5	Targeting peptide	K_16_Cys-S-S-[PEG_6_-IPQA] 1
CLA1	39	21	15	23	2	**P1**	—
CLA2	39	44	15	0	2	**P1**	—
CLA3	39	37	23	0	2	**P1**	—
CLA4	30	37	23	8	2	**P1**	—
NAE1	39	21	15	23	2	**P5**	—
NAE2	39	44	15	0	2	**P5**	—
NAE3	39	37	23	0	2	**P5**	—
NAE4	30	37	23	8	2	**P5**	—
NGE1	39	21	15	23	2	**P5**	—
NGE2	39	44	15	0	2	**P6**	—
NGE3	39	37	23	0	2	**P6**	—
NGE4	30	37	23	8	2	**P6**	—
F1LA1	39	21	15	23	2	**P1**	8 μM
F1LA2	39	21	15	23	2	**P1**	16 μM
F1LA3	39	21	15	23	2	**P1**	32 μM
F2LA1	39	44	15	0	2	**P1**	8 μM
F2LA2	39	44	15	0	2	**P1**	16 μM
F2LA3	39	44	15	0	2	**P1**	32 μM

**Fig. 2:**
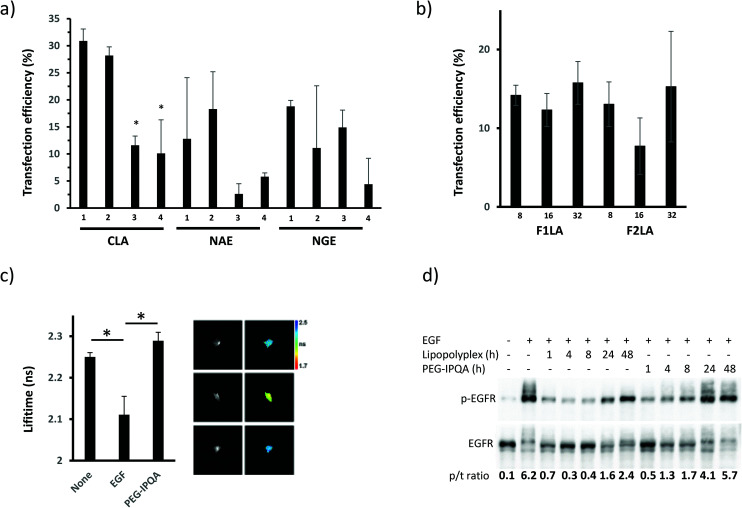
Effect of different lipopolyplex formulations and drug loading on the expression of EGFR in targeted cells and inhibition of its activity upon stimulation with EGF. LIM1215 cells transfected with different lipopolyplexes without inhibitor (CLA1–4, NAE1–4, NGE1–4) (a) or with increasing amount of inhibitor per particle (F1LA1–3, F2LA1–3) (b) 24 h after seeding. High resolution images of 2 × 2 mm area used to quantify transfection efficiency (number of cells expressing biosensor, GFP channel, over total number of cells in field of view, UV channel. (c) FRET-FLIM analysis of the inhibition of EGF-induced EGFR activity in cells by free K_16_Cys-S-S-[PEG_6_-IPQA] **1** (10 mM). *P = 0.03, the difference is statistically significant between the groups. (d) Time course of the inhibitory effect of K_16_Cys-S-S-[PEG_6_-IPQA] **1** delivered to the cells by lipopolyplex or as a free agent.

In contrast to our initial screen, these studies demonstrated that the CLA1 formulation, formulated with **P1** (Table 1) gave the maximum transfection efficiency (Fig. 2A, S3 and S4†), and the optimal final concentration of lipopolyplexes in the media was found to be 10 μM (based on the concentration of the lipids) (Fig. S6†). We then prepared variant lipopolyplex formulations, based on CLA1 and including the EGFR inhibitor K_16_Cys-S-S-[PEG_6_-IPQA] **1** (Table 1). These lipopolyplex formulations (with increasing amount of inhibitor per particle) gave slightly decreased transfection efficiency (Fig. 2B) compared to particles without the drug (Fig. 2A), however the overall number of transfected tumor cells was not affected. For efficient perfusion through the leaky endothelial cells of the tumor, lipopolyplexes must be <150 nm in diameter. The lipopolyplex formulations were characterised using dynamic light scattering (DLS) and zeta potential measurements (ESI Table S1†) and were found to have particle sizes within the ideal diameter for tumor delivery, reasonable polydispersities, and positive zeta potentials which would minimize particle aggregation and afford a reasonable degree of serum stability. We also carried out *in vitro* studies to check that the disulfide linker between K_16_Cys and PEG-IPQA was labile under pseudo-intracellular conditions (GSH/GSSG, HEPES, pH 7.2).^66^ These indicated that under reducing conditions the linkage would be cleaved within 5 min (ESI Fig. S7†). Next, we assessed the effectiveness of K_16_Cys-S-S-[PEG_6_-IPQA] **1** as an inhibitor of EGFR activity as a free therapeutic or within lipopolyplexes by utilizing FRET-FLIM and validating our results by western blot (WB) techniques. Activation of EGFR in cells treated with EGF (natural ligand for EGFR) was evident by decreasing lifetime of green fluorescent protein (GFP) in Picchu-X biosensor (Fig. 2c) and by appearance of phosphorylated EGFR band in WB image (Fig. 2d). In both cases pre-treatment with MeO-[PEG_6_-IPQA] **8** for 1 h abolished these changes: lifetime of GFP remained at baseline levels (Fig. 2c) and the level of EGFR phosphorylation was significantly lower than in the sample without inhibitor (Fig. 2d, line 8 *vs*. line 2).

Furthermore, incorporation of K_16_Cys-S-S-[PEG_6_-IPQA] **1** within lipopolyplexes not only decreased EGFR activity to equally low levels as the free drug but was effective for longer duration (Fig. 2d, lines 3–7 *vs*. line 2). We observed EGFR inhibition when **1** was delivered within lipopolyplexes for up to 48 h, whereas free MeO-[PEG_6_-IPQA] **8** was effective only for 24 h (Fig. 2d). These results clearly reveal F1LA1 to be the optimal formulation for lipopolyplexes to achieve maximum transfection efficiency and prolong inhibition of EGFR in cells stimulated with ligand.

### Radiolabeling and biodistribution

**Scheme 2:**
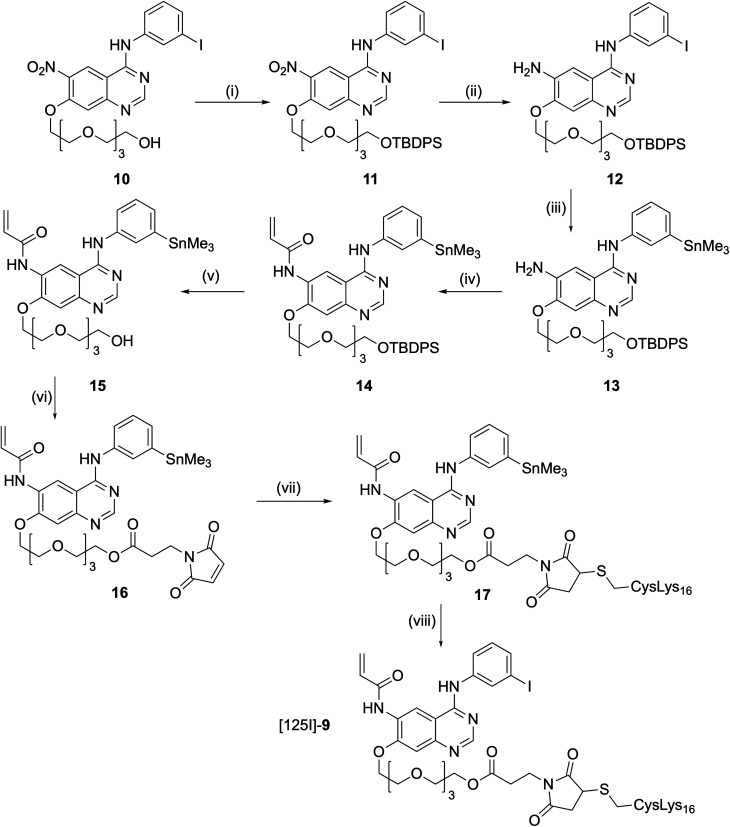
Reagents and conditions: (i) TBDPSCl, imidazole, CH_2_Cl_2_, 0 °C to rt, 3 h (98%); (ii) SnCl_2_·2H_2_O, THF, 60 °C, 3.5 h, (65%); (iii) Pd(PPh_3_)_4_, Sn_2_Me_6_, dioxane, reflux, 2.5 h, (95%); (iv) isobutyl chloroformate, acrylic acid, Et_3_N, −40 °C, 30 min (53%); (v) TBAF, THF, 0 °C to rt, 1 h (95%); (vi) *N*-maleoyl-β-alanine, DCC, DMAP, CH_2_Cl_2_, rt, 2 h, (89%); (vii) CysLys_16_**7**, NaHCO_3_, rt, 1 h (55%); (viii) sodium [^125^I]iodide, iodogen, 30 min rt, 28% radiochemical yield.

In order to assess the *in vivo* biodistribution of these liposome-based theragnostic nanoparticles, we required a [^125^I]-labelled analogue of **1**. As we particularly wished to measure the biodistribution of the IPQA–peptide bioconjugate, we elected to attach the radiolabelled PEG-[^125^I]IPQA moiety to K_16_Cys *via* a maleimide linkage, which would not be subject to reduction *in vivo*. The analogue K_16_Cys-SMal-[PEG_3_-[^125^I]IPQA] **9** was therefore designed with a similar linker length between the K_16_ DNA-binding peptide and IPQA analogue **1**. PEG_3_-IPQA precursor **10** was prepared using a modification of the literature procedure^58^ reported for PEG_6_-IPQA (ESI†). Similar methodology to that developed for the synthesis of **6** was used to access the key stannylated intermediate **16**. Thus, the free PEG terminus of **10** was protected to give TBDPS (^t^butyldiphenylsilyl) ether **11**, followed by reduction to give amine **12**. Stannylation to give **13** and amide formation to afford **14** were followed by TBDPS deprotection to give **15**. *N*-Maleoyl-β-alanine was then coupled to the PEG chain to give the key intermediate **16**. Conjugation of **16** to K_16_Cys (**7**) gave **17** as a precursor for radiolabeling. Incubation of **17** with sodium [^125^I]iodide in an iodogen coated tube (30 min, ambient temperature), followed by purification with semi-preparative radio-HPLC, provided the desired K_16_Cys-SMal-[PEG_3_-[^125^I]-IPQA] **9** in 28% radiochemical yield (Scheme 2).

Radiolabeled [^125^I]-**9** was subsequently formulated as the F1LA1 K_16_-Cys-SMal-[PEG_3_-[^125^I]-IPQA]-lipopolyplex, which was obtained in 64% radiochemical yield. Radiolabeled (MeO-[PEG_6_-[^125^I]-IPQA]) **8** (Scheme 1) was prepared using the same protocol as for [^125^I]-**9**, and was obtained in 38% radiochemical yield. The two radiolabeled tracers, [^125^I]-**8** and F1LA1 K_16_-Cys-SMal-[PEG_3_-[^125^I]-IPQA]-lipopolyplex, were synthesized in order to determine the biodistributions of the small molecule TKI inhibitor and the nanoparticle construct, respectively.

### *In vivo* results

**Fig. 3:**
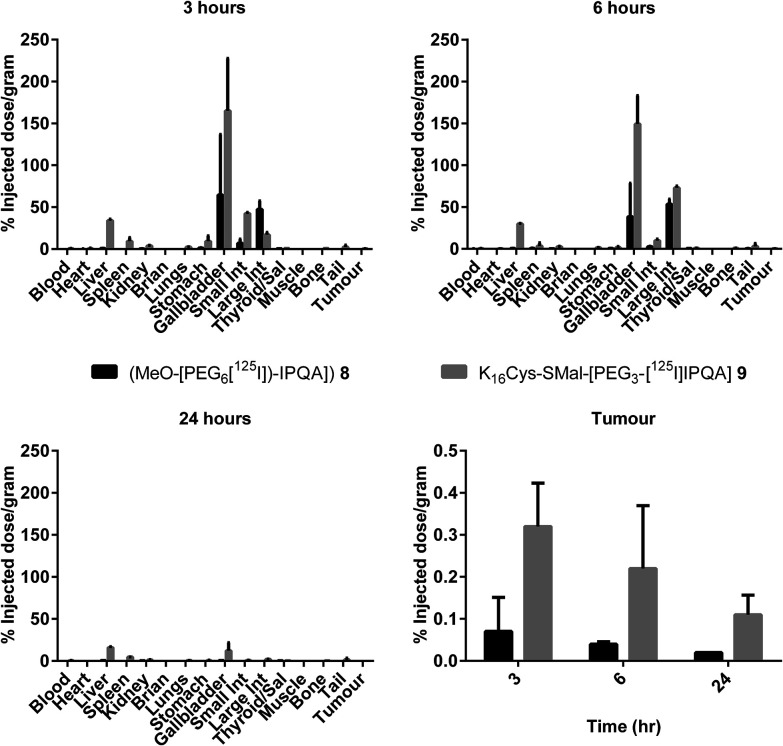
*In vivo* radioactive organ biodistribution data presented in % Injected Dose per gram (%ID g^−1^) for MeO-[PEG_6_-[^125I^]-IPQA] **8** (black columns) and F1LA1 K_16_Cys-SMal-[PEG_3_-[^125^I]-IPQA] **9** lipopolyplex (grey columns) 3, 6 and 24 h after injection. The tumor %ID g^−1^ at 3, 6 and 24 h for both tracers is represented on a separate graph.

LIM1215 xenografts were established in standard immunocompromised CD1 nude mice (Materials and methods section). The biodistribution data (Fig. 3) showed that the free drug, radiolabelled MeO-[PEG_6_-[^125^I]-IPQA] **8** was rapidly cleared *via* the hepatobiliary route with high uptake in gallbladder and intestines at 3 and 6 h.

Whereas F1LA1 K_16_Cys-SMal-[PEG_3_-[^125^I]-IPQA]-lipopolyplex showed uptake within the liver and spleen as well as high uptake in the gallbladder and intestines consistent with lipopolyplex uptake and degradation by the Kupffer cells of the reticuloendothelial system. Tumor uptake was highest for both MeO-[PEG_6_-[^125^I]-IPQA] **8** and F1LA1 K_16_Cys-SMal-[PEG_3_-[^125^I]-IPQA]-lipopolyplex at 3 h with gradual reduction over the 24 h time course. However, the uptake of F1LA1 K_16_Cys-SMal-[PEG_3_-[^125^I]-IPQA]-lipopolyplex within tumors was higher than free MeO-[PEG_6_-[^125^I]-IPQA] **8** at all time points.

**Fig. 4:**
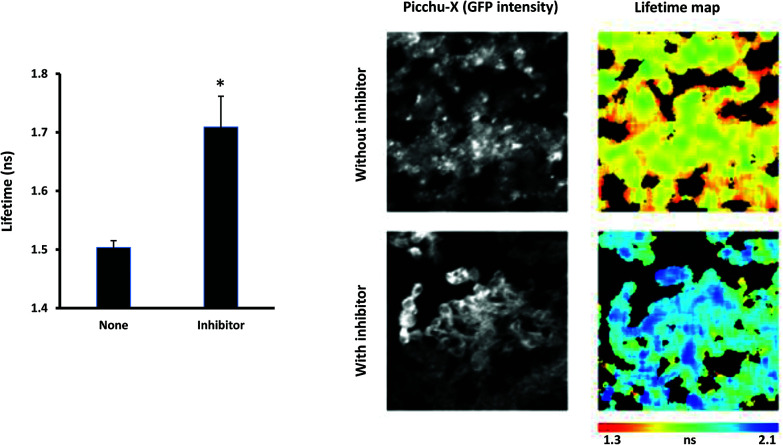
The effect of the inhibitor on EGFR activity measured by Picchu-X biosensor delivered by lipopolyplexes to tumor cells *in vivo*. Fresh frozen LIM1215 xenografts from animals injected with CLA1 lipopolyplex with pDNA encoding for Picchu-X biosensor alone, or F1LA1 K_16_Cys-SMal-[PEG_3_-[^125^I]-IPQA]-lipopolyplex (additionally loaded with K_16_Cys-SMal-[PEG_3_-[^125^I]-IPQA]) were cut and lifetime images were taken (see Material and methods section). Tumors from control group (injected with lipopolyplex without inhibitor) exhibited low lifetime of GFP in the biosensor (yellow-red colors in pseudocolor map) indicating high level of EGFR activation in the cells. In tumors from animals injected with lipopolyplex loaded with EGFR inhibitor we found high lifetime of GFP (blue color on pseudocolor map) indicating inhibition of EGFR activity in cells. *P = 0.004 (*N* = 8 images per group), the difference is statistically significant between the groups.

After gamma counting, the tumors were snap frozen, allowing simultaneous expression of biosensor and inhibition of EGFR activity by the liposome-encapsulated K_16_Cys-SMal-[PEG_3_-[^125^I]-IPQA] **9**. Fig. 4 shows representative images of tissue from mice injected with CLA1 lipopolyplexes, which only contain the pDNA encoding for the Picchu-X biosensor (top images) and from mice injected with F1LA1 K_16_Cys-SMal-[PEG_3_-[^125^I]-IPQA]-lipopolyplexes, which contain both Picchu-X biosensor pDNA and PEG-IPQA (bottom images). Lifetime measurements of GFP revealed that EGFR activity can be successfully suppressed (Fig. 4) by PEG-IPQA released from the F1LA1 K_16_Cys-SMal-[PEG_3_-[^125^I]-IPQA]-lipopolyplexes. Tissues treated with inhibitor had significantly higher GFP lifetime than control tissues (Fig. 4), which can also be seen on the images (shift from red-yellow to blue colours in pseudocolor map).

## Conclusions

Liposome-based theragnostic nanoparticles hold considerable promise for the delivery of small molecule therapeutic drugs to tumours, and for determining their biodistribution and efficacy *in vivo*. A major barrier to the success of such approaches is the difficulty encountered in loading sufficient quantities of the drug into the liposome-based formulation, particularly where co-delivery of DNA or RNA is required.^67^ In this work, we present a novel solution to this problem. The reversible bioconjugation of the small molecule therapeutic to a cationic peptide allows the co-formulation of both a TKI EGFR inhibitor and pDNA coding for a biosensor into a targeted lipopolyplex. The lipopolyplex is designed to disassemble on internalisation into the target cells. Our results indicate that release of the components from the endosome is accompanied by release of the TKI from the bioconjugate and inhibition of EGFR.

This approach also allowed us to further investigate the basis for the enhanced circulation and better tumour selectivity afforded by nanoparticle delivery of small molecule therapeutics. The biodistribution data in Fig. 3 suggests different kinetics for either uptake of free drug *vs*. liposomes, and/or elimination of the free drug *vs*. drug encapsulated/shielded in liposome in the cells. This phenomenon will need further investigation of potential differences in trafficking of the drug within the cell post-uptake, both in terms of the subcellular compartments involved and kinetics of intracellular trafficking. However, we believe that the *in vivo* trafficking/clearance differences may be more significant and therefore relevant for the therapeutic effectiveness. The clearance *in vivo* of radiolabelled Mo-IPQA has recently been studied,^30,57^ and has shown that this highly lipophilic inhibitor has limited tumour selectivity and undergoes significant hepatobiliary clearance. More hydrophilic PEGylated analogues, such as [^18^F]F-PEG_6_-IPQA,^68^ which formed the basis for the design of our own bioconjugate, show improved pharmacokinetics and quicker *in vivo* clearance, but still have little selectivity for tumour cells.^69,70^ In contrast, liposomal formulations of the structurally-related, radiolabelled quinazoline TKI inhibitor, SKI-212243 had a significantly longer circulation time, increased overall tumour uptake and selectivity compared to the free drug.^19^ Moreover, a different pattern of biodistribution was observed, suggesting that the liposomal formulation is cleared through a different route to the free drug.

Many potent TKI have been developed as anti-EGFR agents, and have shown excellent inhibitory properties *in vitro*. However, translation to clinically viable agents, particularly for the treatment of colorectal cancer, has generally been unsuccessful, and the reasons for the poor clinical efficacy of these treatments remains elusive. Knowing how these therapies actually act at a molecular level when administered to patients would be a crucial step towards improving the success rate of such inhibitors. Our results demonstrate that lipopolyplexes are a successful delivery system for both an EGFR biosensor and a TKI inhibitor, which will allow surveillance of EGFR activity while testing the potency of such drugs *in vivo*. This approach in turn opens a new possibility for manipulating and monitoring the activity of EGFR *in situ*, which in turn will allow a deeper understanding of the dynamics of EGFR activation/inhibition and sensitivity of target cells to EGFR inhibition. Whilst we have previously demonstrated that it is possible to image EGFR activity *in vivo* utilizing an endoscopy-based fluorescence lifetime imaging methodology,^71^ this was based on an endoscope requiring physical contact with the area of interest to be imaged, and is a highly invasive procedure. Currently, we envisage that the most useful application of our methodology will be to investigate human derived xenografts to test the responsiveness of the tumour to different treatment regimes.

Finally, we have demonstrated that the reversible bioconjugation of TKIs to cationic peptides and their formulation into targeted nanoparticles results in greatly improved biodistribution relative to the free drug. This in turn will minimize toxicity due to off-target effects. Ultimately, we believe that this approach can be generalised. It will allow for the targeted co-delivery of small molecule/toxic gene, small molecule/gene therapy, and small molecule/siRNA combinations,^72^ opening up exciting possibilities for combined therapeutic delivery and sensing.

## Experimental section

### Chemical synthesis

General methods for chemical synthesis and solid phase peptide synthesis, sources of chemicals, purification and analytical methods are in the ESI.† Peptides **P1**,^49^**P2**,^49^**P3**,^49^**P4**,^30^ and **P6**^30^ were synthesized according to the published literature procedures. Experimental procedures for the synthesis of **3**, **8**, **10**, **S6**, **P5** and **7**; HPLC/HRMS for peptides **1**, **17**, **P5**, **7** and ^1^H/^13^C NMR spectra for novel compounds can be found in the ESI.†

### Synthesis of K_16_Cys-S-S-[PEG_6_-IPQA] (**1**)

#### *N*-(3-Iodophenyl)-6-nitro-7-((1,1,1-triphenyl-5,8,11,14,17-pentaoxa-2-thianonadecan-19-yl)oxy)quinazolin-4-amine (**4**)

Compound **3** (702 mg, 0.940 mmol) and triphenylmethane thiol (310 mg, 1.12 mg) were dissolved in THF (16 mL) and NaH (89 mg, 3.7 mmol) was added gradually. The dark purple reaction mixture was stirred for 2 h at rt and subsequently quenched with cold NaHCO_3_-solution. The aqueous phase was extracted (3 × CH_2_Cl_2_) and the combined organic phases washed with saturated NaCl solution (2×). The combined organic phases were dried (MgSO_4_), filtered and concentrated *in vacuo*. Purification was achieved using flash column chromatography (3% MeOH/CH_2_Cl_2_) to give **4** (712 mg, 82%) as a yellowish oil. ^1^H NMR (600 MHz, CDCl_3_) *δ* 8.86 (s, 1H), 8.71 (s, 1H), 8.25 (s, 1H), 7.82 (d, *J* = 8.1 Hz, 1H), 7.50 (d, *J* = 7.9 Hz, 1H), 7.30–7.33 (m, 6H), 7.20–7.23 (m, 7H), 7.15–7.17 (m, 3H), 7.11 (t, *J* = 8.0 Hz, 1H), 4.22–4.23 (m, 2H), 3.84–3.85 (m, 2H), 3.69–3.71 (m, 2H), 3.61–3.64 (m, 2H), 3.47–3.52 (m, 6H), 3.40–3.44 (m, 6H), 3.25 (t, *J* = 6.8 Hz, 2H), 2.33 (t, *J* = 6.8 Hz, 2H) ppm; ^13^C NMR (150 MHz, CDCl_3_) *δ* 158.2, 157.4, 155.0, 144.8, 139.32, 139.29, 133.9, 131.4, 130.5, 129.5, 128.04, 127.99, 126.7, 122.3, 121.7, 110.0, 108.2, 94.1, 71.3, 70.8, 70.7, 70.56, 70.52, 70.46, 70.42, 70.2, 69.9, 69.8, 69.3, 66.6, 31.6 ppm; HRMS (ESI) *m*/*z*: [M + H]+ Calcd for C_45_H_48_IN_4_O_8_S 931.2238; Found 931.2240.

#### *N*-(3-Iodophenyl)-6-amino-7-((1,1,1-triphenyl-5,8,11,14,17-pentaoxa-2-thianonadecan-19-yl)oxy)quinazolin-4-amine (**5**)

Compound **4** (50 mg, 0.054 mmol) was dissolved in THF (10 mL) and SnCl_2_·2H_2_O (30 mg, 0.16 mmol) was added. The reaction was heated to reflux for 3 h then cooled to rt. Saturated NaHCO_3_ solution (20 mL) was added followed by H_2_O (50 mL). The aqueous phase was extracted (3 × EtOAc) and the combined organic phases dried (MgSO_4_), filtered and concentrated *in vacuo*. Purification was achieved using flash column chromatography (3% MeOH/CH_2_Cl_2_) to give **5** (30 mg, 61%) as a yellowish oily solid. ^1^H NMR (600 MHz, CDCl_3_) *δ* 8.57 (s, 1H), 8.12–8.13 (m, 1H), 7.73 (d, *J* = 8.0 Hz, 1H), 7.43 (br s, 1H), 7.41–7.42 (m, 1H), 7.32–7.34 (m, 6H), 7.20–7.22 (m, 6H), 7.14–7.17 (m, 4H), 7.12 (s, 1H), 7.07 (t, *J* = 8.0 Hz, 1H), 4.62 (br s, 2H), 4.28–4.30 (m, 2H), 3.91–3.92 (m, 2H), 3.75–3.76 (m, 2H), 3.57–3.67 (m, 12H), 3.52–3.54 (m, 2H), 3.31 (t, *J* = 6.8 Hz, 2H), 2.39 (t, *J* = 6.8 Hz, 2H) ppm; ^13^C NMR (150 MHz, CDCl_3_) *δ* 155.3, 152.6, 151.5, 145.2, 144.8, 140.6, 138.8, 132.4, 130.5, 129.7, 129.6, 128.0, 126.7, 120.4, 110.6, 107.6, 101.0, 94.2, 71.1, 70.8, 70.78, 70.76, 70.73, 70.63, 70.55, 70.2, 69.8, 69.1, 68.4, 66.7, 31.6 ppm; HRMS (ESI) *m*/*z*: [M + H]+ Calcd for C_45_H_50_IN_4_O_6_S 901.2496; Found 901.2498.

#### *N*-(4-((3-Iodophenyl)amino)-7-((1,1,1-triphenyl-5,8,11,14,17-pentaoxa-2-thianonadecan-19-yl)oxy)quinazolin-6-yl)acrylamide (**6**)

A solution of acrylic acid (50 μL, 0.74 mmol) in THF (2.5 mL) was cooled to 0 °C and treated with isobutylchloroformate (81 μL, 0.62 mmol), added dropwise, followed by Et_3_N (175 μL, 1.26 mmol). The reaction was cooled to −40 °C using an acetonitrile/dry ice bath and a solution of **5** (192 mg, 0.211 mmol) in THF (1 mL) was added in one portion. The resultant reaction mixture was stirred for 35 min and then quenched with saturated NaHCO_3_ solution. The solution was extracted (3 × CH_2_Cl_2_) and the combined organic phases dried (MgSO_4_), filtered and concentrated *in vacuo*. Purification was achieved using automated flash column chromatography (0–2% MeOH/CH_2_Cl_2_) to give **6** (120 mg, 57%) as a yellowish oil. ^1^H NMR (600 MHz, CDCl_3_) *δ* 9.14 (s, 1H) 8.68 (s, 1H) 8.59 (s, 1H) 8.20 (t, *J* = 1.8 Hz, 1H) 7.74 (ddd, *J* = 8.2, 2.2, 0.9 Hz, 1H) 7.56 (s, br, 1H) 7.48 (ddd, *J* = 7.9, 1.6, 1.0 Hz, 1H) 7.37–7.43 (m, 6H) 7.24–7.33 (m, 6H) 7.17–7.22 (m, 3H) 7.12 (t, *J* = 8.0 Hz, 1H) 6.50 (dd, *J* = 16.8, 1.5 Hz, 1H) 6.43 (dd, *J* = 16.8, 9.8 Hz, 1H) 5.84 (dd, *J* = 9.8, 1.5 Hz, 1H) 4.36–4.39 (m, 2H) 3.94–4.00 (m, 2H) 3.73–3.77 (m, 2H) 3.68–3.72 (m, 2H) 3.57–3.67 (m, 8H) 3.53–3.56 (m, 2H) 3.40–3.46 (m, 2H) 3.29 (t, *J* = 6.9 Hz, 2H) 2.41 (t, *J* = 6.9 Hz, 2H) ppm; ^13^C NMR (150 MHz, CDCl_3_) *δ* 164.3, 156.8, 154.2, 152.4, 144.9, 139.6, 133.3, 131.3, 130.5, 129.7, 128.6, 128.6, 128.04, 127.99, 127.4, 126.8, 121.1, 110.0, 107.8, 94.2, 70.8, 70.7, 70.62, 70.56, 70.2, 69.7, 69.0, 68.9, 66.7, 31.7 ppm (3 OCH_2_ peaks overlapping – evident by HSQC); HRMS (ESI) *m*/*z*: [M + H]+ Calcd for C_48_H_52_IN_4_O_7_S 955.2601; Found 955.2528.

#### *N*-(4-((3-Iodophenyl)amino)-7-((1-((5-nitropyridin-2-yl)disulfanyl)-2,5,8,11,14-pentaoxahexadecan-16-yl)oxy)quinazolin-6-yl)acrylamide (**2**)

To a solution **6** (50 mg, 0.050 mmol) in CH_2_Cl_2_ (5 mL) was added DTNP (24 mg, 0.078 mmol) and TFA (500 μL, 6.53 mmol). The reaction mixture was stirred for 2 h at rt. The solvent was removed *in vacuo* and purification was achieved using flash column chromatography (5% MeOH/CH_2_Cl_2_) to give **2** (21 mg, 48%) as a yellow oil. ^1^H NMR (600 MHz, CDCl_3_) *δ* 9.23 (dd, *J* = 2.6, 0.7 Hz, 1H), 9.12 (s, 1H), 8.73 (s, 1H), 8.67 (s, 1H), 8.42 (dd, *J* = 8.9, 2.6 Hz, 1H), 8.20 (t, *J* = 1.8 Hz, 1H), 8.02 (dd, *J* = 8.9, 0.8 Hz, 1H), 7.79 (br s, 1H), 7.75 (ddd, *J* = 8.1, 1.8, 0.8 Hz, 1H), 7.48 (ddd, *J* = 7.9, 1.4, 0.9 Hz, 1H), 7.32 (s, 1H), 7.11 (t, *J* = 8.0 Hz, 1H), 6.43–6.49 (m, 2H), 5.83 (dd, *J* = 7.4, 4.1 Hz, 1H), 4.37–4.39 (m, 2H) 3.96–3.98 (m, 2H) 3.73–3.75 (m, 2H) 3.60–3.71 (m, 14H), 3.53–3.55 (m, 2H), 3.01 (t, *J* = 6.0 Hz, 2H) ppm; ^13^C NMR (150 MHz, CDCl_3_) *δ* 169.3, 164.3, 154.1, 152.5, 145.0, 142.1, 139.7, 133.3, 132.6, 132.0, 131.3, 131.0, 130.6, 130.5, 128.6, 128.5, 121.1, 119.5, 110.2, 109.6, 107.7, 94.2, 70.8, 70.74, 70.66, 70.642, 70.615, 70.56, 69.1, 68.9, 68.5, 39.0 ppm (3 OCH_2_ peaks overlapping); HRMS (ESI) *m*/*z*: [M + H]+ Calcd for C_34_H_40_IN_6_O_9_S_2_I 867.1335; Found 867.1337.

#### K_16_Cys-S-S-[PEG_6_-IPQA] (**1**)

A solution of CysLys_16_**7** (5 mg) and **2** (3 mg) in MeOH were stirred in MeOH at room temperature for 1 h, monitoring by analytical HPLC. The solvent was removed *in vacuo* and the resulting biconjugate was purified by HPLC to give **1** (5.9 mg, 59% yield).

### Synthesis of K_16_-Cys-SMal-[PEG_3_-*N*-(4-((3-trimethylstannyl)amino)quinazolin-6-yl)acrylamide] (**17**)

#### 7-((2,2-Dimethyl-3,3-diphenyl-4,7,10,13-tetraoxa-3-silapentadecan-15-yl)oxy)-*N*-(3-iodophenyl)-6-nitroquinazolin-4-amine (**11**)

A solution of **10** (200 mg, 0.342 mmol) and imidazole (69 mg, 1.03 mmol) in DCM (10 mL) was cooled to 0 °C and treated with TBDPS-Cl (179 μL, 0.685 mmol), added slowly. The reaction mixture was allowed to warm to rt and stirred for 3 h. After this time the reaction mixture was diluted with CH_2_Cl_2_ and washed with saturated NaCl solution (2×) and H_2_O. The organic layer was dried (MgSO_4_), filtered and concentrated *in vacuo*. Purification was achieved using flash column chromatography (0–2% MeOH /CH_2_Cl_2_) to give **11** (276 mg, 98%) as yellow amorphous solid. ^1^H NMR (600 MHz, CDCl_3_) *δ* 8.75 (s, 1H), 8.68 (s, 1H), 8.25 (t, *J* = 1.9 Hz, 1H), 8.20 (br s, 1H), 7.77 (ddd, *J* = 8.1, 2.1, 0.8 Hz, 1H), 7.61–7.63 (m, 4H), 7.51 (ddd, *J* = 7.8, 1.5, 0.8 Hz, 1H), 7.33–7.41 (m, 6H), 7.11–7.14 (m, 2H), 4.14–4.16 (m, 2H), 3.87–3.89 (m, 2H), 3.74–3.78 (m, 4H), 3.63–3.70 (m, 6H), 3.51 (t, *J* = 5.5 Hz, 2H), 1.00 (s, 9H) ppm; ^13^C NMR (150 MHz, CDCl_3_) *δ* 158.1, 157.9, 154.7, 153.7, 139.2, 139.1, 135.7, 133.9, 133.7, 130.9, 130.5, 129.7, 127.7, 121.4, 121.1, 110.6, 108.2, 94.2, 72.4, 71.2, 70.9, 70.81, 70.75, 69.7, 69.1, 63.4, 26.9, 19.3 ppm; HRMS (ESI) *m*/*z*: [M + H]+ Calcd for C_38_H_44_IN_4_O_7_Si 823.2018; Found 823.2018.

#### 7-((2,2-Dimethyl-3,3-diphenyl-4,7,10,13-tetraoxa-3-silapentadecan-15-yl)oxy)-*N*-(3-iodophenyl)quinazoline-4,6-diamine (**12**)

A solution of **11** (690 mg, 0.830 mmol) in THF (9 mL) was treated with SnCl_2_·H_2_O (605 mg, 2.63 mmol). The reaction mixture was heated to 60 °C for 3 h followed by heating to reflux for 30 min. After this time the reaction mixture was cooled to rt and H_2_O (50 mL) and saturated NaHCO_3_ solution (20 mL) were added. The aqueous phase was extracted (3 × EtOAc). The combined organic phases were washed with saturated NaCl solution and H_2_O, dried (MgSO_4_), filtered and concentrated *in vacuo*. Purification was achieved using automated flash column chromatography (0–20% MeOH/CH_2_Cl_2_) to give **12** (430 mg, 65%) as a yellow oil. ^1^H NMR (600 MHz, CDCl_3_) *δ* 8.57 (s, 1H), 8.13 (t, *J* = 2.2 Hz, 1H), 7.68–7.70 (m, 5H), 7.36–7.46 (m, 8H), 7.14 (s, 1H), 7.11 (m, 1H), 6.85 (s, 1H), 4.28–4.30 (m, 2H), 3.93–3.94 (m, 2H), 3.83 (t, *J* = 6.1 Hz, 2H), 3.70–3.75 (m, 4H), 3.67 (s, 4H), 3.61 (t, *J* = 6.1 Hz, 2H), 1.06 (s, 9H) ppm; ^13^C NMR (150 MHz, CDCl_3_) *δ* 155.1, 152.5, 151.4, 140.1, 138.3, 135.7, 133.8, 132.8, 130.5, 129.85, 129.80, 127.8, 127.7, 120.5, 110.2, 107.2, 100.4, 94.2, 72.6, 70.93, 70.86, 70.8, 70.7, 69.4, 68.3, 63.6, 27.0, 19.3 ppm; HRMS (ESI) *m*/*z*: [M + H]+ Calcd for C_38_H_46_IN_4_O_5_Si 793.2282; Found 793.2290.

#### 7-((2,2-Dimethyl-3,3-diphenyl-4,7,10,13-tetraoxa-3-silapentadecan-15-yl)oxy)-*N*-(3-trimethylstannyl)quinazoline-4,6-diamine (**13**)

A stream of argon was bubbled through a solution of **12** (430 mg, 0.542 mmol) in dioxane (20 mL) for 10 min. Pd(PPh_3_)_4_ (15 mg, 0.012 mmol) was added followed by hexamethylditin (355 mg, 224 μL, 1.08 mmol) and the resultant solution heated at reflux for 2.5 h. The reaction mixture was cooled to rt and the solvent evaporated *in vacuo*. CH_2_Cl_2_ and saturated NaHCO_3_ solution were added and the phases separated. The aqueous phase was washed (2 × CH_2_Cl_2_) and the combined organic phases washed with saturated NaCl solution then dried (MgSO_4_), filtered and concentrated *in vacuo*. Purification was achieved using automated flash column chromatography (0–10% MeOH/CH_2_Cl_2_) to give **13** (428 mg, 95%) as a yellow oil. ^1^H NMR (600 MHz, CDCl_3_) *δ* 8.57 (s, 1H), 7.80 (ddd, *J* = 8.0, 2.2, 1.0 Hz, 1H), 7.67–7.70 (m, 5H), 7.61 (d, *J* = 2.3 Hz, 1H), 7.36–7.42 (m, 8H), 7.17 (s, 1H), 7.11 (t, *J* = 9.7 Hz, 1H), 6.95 (br s, 1H), 6.85 (s, 1H), 4.30–4.35 (m, 4H), 3.94–3.95 (m, 2H), 3.83 (t, *J* = 5.1 Hz, 2H), 3.70–3.75 (m, 4H), 3.66 (s, 4H), 3.62 (t, *J* = 5.1 Hz, 2H), 1.06 (s, 9H), 0.32 (s, 9H) ppm; ^13^C NMR (150 MHz, CDCl_3_) *δ* 155.6, 152.3, 152.1, 143.4, 138.4, 137.9, 135.7, 133.8, 131.6, 129.8, 128.7, 128.6, 127.80, 127.76, 122.0, 110.3, 107.5, 100.6, 72.6, 70.95, 70.89, 70.8, 70.7, 69.4, 68.2, 63.6, 27.0, 19.3, −9.3 ppm; HRMS (ESI) *m*/*z*: [M + H]+ Calcd for C_41_H_55_N_4_O_5_SiSn 831.2964; Found 831.2973.

#### 7-((2,2-Dimethyl-3,3-diphenyl-4,7,10,13-tetraoxa-3-silapentadecan-15-yl)oxy)-*N*-(4-((3-trimethylstannyl)amino)quinazolin-6-yl)acrylamide (**14**)

A solution of acrylic acid (63 mg, 60 μl, 0.88 mmol) in THF (8 mL) was cooled to 0 °C and treated with isobutylchloroformate (104 mg, 99 μl, 0.76 mmol), added dropwise, followed by Et_3_N (163 μL, 1.26 mmol). The reaction was stirred at 0 °C for 10 min then the ice bath was replaced with an acetonitrile/dry ice bath and left for 2 min more. A solution of **13** (240 mg, 0.290 mmol) in THF (4 mL) was cooled in the dry ice bath and then added to the reaction in one portion, the resultant reaction mixture was stirred for 30 min and then quenched with saturated NaHCO_3_ solution. The solution was extracted (3 × CH_2_Cl_2_) and the combined organic phases were washed with saturated NaCl solution then dried (MgSO_4_), filtered and concentrated *in vacuo*. Purification was achieved using automated flash column chromatography (4% MeOH/CH_2_Cl_2_) to give **14** (137 mg, 53%) as a yellow oil. ^1^H NMR (600 MHz, CDCl_3_) *δ* 9.13 (s, 1H), 8.94 (br s, 1H), 8.64 (s, 1H), 7.86 (dd, *J* = 8.0, 2.0 Hz, 1H), 7.66–7.68 (m, 5H), 7.35–7.42 (m, 7H), 7.29 (d, *J* = 7.0 Hz, 1H), 7.24 (s, 1H), 6.47–6.49 (m, 2H), 5.76 (dd, *J* = 8.2, 3.1 Hz, 1H), 4.35–4.37 (m, 2H), 3.96–3.97 (m, 2H), 3.81 (t, *J* = 5.3 Hz, 2H), 3.64–3.76 (m, 8H), 3.61 (t, *J* = 5.3 Hz, 2H), 1.04 (s, 9H), 0.33 (s, 9H) ppm; ^13^C NMR (150 MHz, CDCl_3_) *δ* 164.4, 157.2, 154.9, 152.2, 148.3, 143.3, 138.0, 135.7, 133.6, 132.0, 131.3, 129.8, 129.2, 128.5, 128.24, 128.21, 127.8, 122.6, 110.5, 109.8, 107.9, 72.5, 70.9, 70.8, 70.7, 70.6, 69.2, 68.5, 63.5, 26.9, 19.3, −9.3 ppm; HRMS (ESI) *m*/*z*: [M + H]+ Calcd for C_44_H_57_N_4_O_6_SiSn 885.3069; Found 885.3074.

#### *N*-(7-(2-(2-(2-(2-Hydroxyethoxy)ethoxy)ethoxy)ethoxy)-4-((3-(trimethylstannyl)phenyl)amino)quinazolin-6-yl)acrylamide (**15**)

A solution of **14** (42 mg, 0.047 mmol) in THF (2 mL) was cooled to 0 °C and treated with TBAF (1 M in THF, 71 μL, 0.071 mmol), added slowly. The reaction mixture was allowed to warm to rt and stirred for 1 h. The organic phase was diluted with EtOAc and washed thoroughly with saturated NH_4_Cl solution to remove traces of TBAF. After filtration and evaporation of the solvent, purification was achieved by flash column chromatography (4% MeOH/CH_2_Cl_2_) to give **15** (29 mg, 95%) as a yellow oil. ^1^H NMR (600 MHz, CDCl_3_) *δ* 9.16 (s, 1H), 8.94 (br s, 1H), 8.65 (s, 1H), 7.86–7.88 (m, 1H), 7.66–7.68 (m, 1H), 7.64–7.73 (m, 1H), 7.59 (br s, 1H), 7.39–7.41 (m, 1H), 7.27–7.29 (m, 1H), 6.50–6.56 (m, 2H), 5.84 (dd, *J* = 7.9, 3.5 Hz, 1H), 4.39–4.41 (m, 2H), 3.98–4.00 (m, 2H), 3.60–3.77 (m, 12H), 0.33 (s, 9H) ppm; ^13^C NMR (150 MHz, CDCl_3_) *δ* 164.4, 157.2, 154.9, 152.3, 148.3, 143.3, 138.0, 131.9, 131.4, 129.1, 128.5, 128.3, 128.2, 122.5, 110.5, 109.8, 108.0, 72.7, 70.9, 70.6, 70.5, 70.3, 69.3, 68.5, 61.6, −9.3 ppm; HRMS (ESI) *m*/*z*: [M + H]+ Calcd for C_28_H_39_N_4_O_6_Sn 639.1912; Found 639.1916.

#### 2-(2-(2-(2-((6-Acrylamido-4-((3-(trimethylstannyl)phenyl)amino)quinazolin-7-yl)oxy)ethoxy)ethoxy)ethoxy)ethyl 3-((2,5-dioxocyclopent-3-en-1-yl)amino)propanoate (**16**)

A solution of *N*-maleoyl-β-alanine (4.7 mg, 0.028 mmol) in CH_2_Cl_2_ (0.5 mL) was treated with DCC (6.2 mg, 0.030 mmol) and a grain of DMAP. The reaction mixture was left stirring for 3 min then a solution of **15** (9 mg, 0.014 mmol) in CH_2_Cl_2_ (0.5 mL) was added in one portion. After 2 h, stirring at rt, the reaction mixture was diluted with CH_2_Cl_2_ and the washed with saturated NaHCO_3_ solution and saturated NaCl solution. The organic phase was dried (MgSO_4_), filtered and concentrated *in vacuo*. Purification was achieved using flash column chromatography (0–5% MeOH/CH_2_Cl_2_) to give **16** (10 mg, 89%) as a yellow oil. ^1^H NMR (600 MHz, CDCl_3_) *δ* 9.14 (s, 1H), 8.64 (s, 1H), 7.86 (ddd, *J* = 8.1, 2.3, 1.1 Hz, 1H), 7.69 (d, *J* = 4.1 Hz, 1H), 7.63 (br s, 1H), 7.40 (t, *J* = 7.3 Hz, 1H), 7.27–7.29 (m, 1H), 6.68 (s, 2H), 6.45–6.48 (m, 2H), 5.84 (dd, *J* = 9.1, 2.2 Hz, 1H), 4.38–4.40 (m, 2H), 4.20–4.22 (m, 2H), 3.98–3.99 (m, 2H), 3.83 (t, *J* = 7.1 Hz, 2H), 3.63–3.78 (m, 10H), 2.65 (t, *J* = 7.1 Hz, 2H), 0.33 (s, 9H) ppm; ^13^C NMR (150 MHz, CDCl_3_) *δ* 170.8, 170.5, 164.1, 157.2, 154.9, 152.1, 148.4, 143.3, 138.0, 134.3, 131.9, 131.4, 129.2, 128.5, 128.25, 128.20, 122.5, 10.3, 109.9, 108.3, 70.8, 70.7, 70.64, 70.62, 69.2, 69.1, 68.7, 63.9, 33.6, 32.9, −9.3 ppm; HRMS (ESI) *m*/*z*: [M + H]+ Calcd for C_35_H_44_N_5_O_9_Sn 798.2156; Found 798.2158.

#### K_16_-Cys-SMal-[PEG_3_-*N*-(4-((3-trimethylstannyl)amino)quinazolin-6-yl)acrylamide] (**17**)

A solution of **16** (3.0 mg), CysLys_16_**7** (10.0 mg) and NaHCO_3_ (5.0 mg) in MeOH (1 mL) was stirred at room temperature for 1 h, monitoring by analytical HPLC. The solvent was removed *in vacuo* and the resulting biconjugate was purified by HPLC to give **17** (6.1 mg, 55% yield).

### Liposome and lipopolyplex formulation

#### Lipopolyplex formulation for *in vitro* experiments

All lipid components were dissolved in CHCl_3_ to a concentration of 1 mM. These stock solutions were mixed to obtain the desired lipid quantities. The solvent was then removed under reduced pressure to form a thin film, which was further dried on a high vacuum line for at least 1 h. The thin film was hydrated with sterilized water to the desired total lipid concentration, sonicated for 10 min, and used immediately. Lipopolyplexes used for *in vitro* experiments were formulated as described (including Cy5-DOTMA 2mol-%). For every 100 μL of sonicated liposome solution, first peptide (4–8 μg) and/or peptide–drug conjugate (8–32 μM) was added followed by plasmid DNA (2–4 μg) coding for the Picchu-X sensor. The samples were mixed and used immediately.

#### F1LA1 K_16_Cys-S-S-[PEG6-IPQA] lipopolyplex formulation for *in vivo* experiments (example for **10** mice)

The following lipid solutions (1 mM in chloroform) were added to a round bottom flask:

DODEG4: 50 mol% → 500 μl of 1 mM lipid stock for 1 mL of liposome solution;

DOTMA: 20 mol% → 200 μl of 1 mM lipid Stock for 1 mL of liposome solution;

DOPE: 30 mol% → 300 μl of 1 mM lipid Stock for 1 mL of liposome solution;

CHOL: 30 mol% → 300 μl of 1 mM lipid Stock for 1 mL of liposome solution.

This gave a total concentration of the lipids in the liposomes of 1.3 mM. The solvent in the lipid mixture was slowly removed *via* evaporation *in vacuo* to form a lipid thin film and dried for at least 1 h on a high vacuum line. The appropriate amount of distilled water was added to reach a concentration of 1.3 mM total lipid in water. The sample was sonicated for 5 min in a bath sonicator and subsequently on a probe tip sonicator over 10 min using a cycle of 10 s sonication at level 3 and 3 s cooling time. The lipid solution was kept on ice during the sonication process to avoid degradation of the lipid components and overheating of the solution. This procedure gives liposomes of <100 nm and surface charge around +20 meV or above.

The liposomes were transferred into a Falcon tube and to this solution was added targeting peptide **P1** (K_16_-RVRR-LARLLT (1 mM stock)) to a final concentration of 170 μM followed by the drug peptide bioconjugate (K_16_Cys-S-S-[PEG_6_-IPQA] **1** (0.7 mM stock)) to a final concentration of 100 μM. The liposome peptide solution was then diluted to 3.5 mL. 680 μg Picchu-X plasmid DNA (1.68 μg μL^−1^ stock, 404 μL) was added to a Falcon tube and diluted to 3.5 mL. The DNA solution was then slowly (dropwise but steadily) added to the liposome–peptide solution while constantly swirling the solution, in order to avoid the concentration of DNA and peptide gets locally too high and precipitates. Once the whole solution has been added the now freshly formed lipopolyplexes have been transferred to a spin-column (MWCO above 5000 Da) and concentrated to the initial volume of 1 mL.

### Radiolabeling

General methods for radioiodination work and HPLC traces can be found in the ESI.†

#### Preparation of MeO-[PEG_6_-[^125^I]-IPQA] (**8**)

A Sep-Pak tC18 Plus Light Cartridge (145 mg, Waters Ltd, Cat. no. WAT036805) was flushed with ethanol (5 mL), HPLC water (10 mL) and was air-dried (10 mL). TKI-tin precursor (**S6**) (1.8 mg) was dissolved in PBS (0.18 mL, Sigma-Aldrich Ltd, Cat. no. P4417). This stock solution (40 μL) was added to an iodogen coated tube (Thermo-Fisher Ltd, Cat. no. 28601) followed by mixing with [^125^I]NaI (5 μL, 34.2 MBq). After standing for 30 min at room temperature the reaction was quenched by adding HPLC mobile phase (100 μL of MeOH/H_2_O/TFA 10/90/0.1 v/v/v). The reaction mixture was injected into a semi-preparative HPLC. The product peak (*t*_R_ = 18.9 min) was collected and diluted with H_2_O (10 mL). A conditioned ^t^C18 SepPak Light cartridge was loaded with that solution, washed with H_2_O (5 mL) and eluted with ethanol in six fractions (0.1 mL). The overall RCY was 38%. The radiochemical purity was >99% as determined by analytical HPLC. The UV channel (254 nm) did not show any stable impurities. The identity of the radioactive product was confirmed by co-injection with a non-radioactive reference solution of 9. The product was formulated by mixing aqueous cold compound (100 μM, 1.1 mL) with an ethanol fraction of [^125^I]-**8** as collected from the SepPak cartridge (6.8 MBq).

#### Preparation of K_16_-Cys-SMal-[PEG_3_-[^125^I]-IPQA] **9**

The radiolabeling procedure for preparing K_16_-Cys-SMal-[PEG_3_-[^125^I]-IPQA] (**9**) starting from the corresponding tin precursor (**17**) (using a stock solution of 13 mg mL^−1^) and [^125^I]NaI (5 μL, 37.5 MBq) followed the same protocol as detailed for [^125^I]-**8**. The overall RCY of [^125^I]-**9** was 28%. The radiochemical purity was >99% as determined by analytical HPLC. The UV channel (254 nm) did not show any stable impurities. The identity of the radioactive product was confirmed by co-injection with a non-radioactive K_16_-PEG-IPQA reference solution.

#### Preparation of the radiolabeled F1LA1 K_16_-Cys-SMal-[PEG_3_-[^125^I]-IPQA]-lipopolyplex

The volume of K_16_Cys-PEG-[^125^I]-IPQA ([^125^I]-**9**) in ethanol was reduced (40 μL, 7.8 MBq) using a stream of nitrogen. Pre-formulated liposome–peptide suspension (1.3 mM lipid mixture (50% DODEG4, 20% DOTMA), 170 mM targeting peptide **P1** K_16_-RVRRLARLLT, 100 μM K_16_Cys-S-S-[PEG_6_-IPQA] **1**) in distilled water (4 mL) was mixed with K_16_Cys-SMal-[PEG_3_-[^125^I]-IPQA] **9**. After slowly adding plasmid DNA (Picchu-X, 0.748 mg) in distilled water (4 mL) with vortexing, the resulting mixture was filtered using four spin columns (VIVASPIN 500, Sartorius Cat. no. VS0151) on a micro-centrifuge (TopSpin, 40 min, 5000 rpm). Please note that this procedure can result in formation of radioactive aerosol, and it must be carried out in a suitable ventilation cabinet with appropriate measures to control radioactive contamination. The final F1LA1 K_16_Cys-SMal-[PEG_3_-[^125^I]-IPQA]-lipopolyplex suspension was collected in 1.1 mL (64% RCY).

### *In vivo* and *in vitro* testing

#### Tissue culture, reagents and antibodies

LIM1215, DiFi, SW48 and DLD1 colon cancer cell lines (independently validated by STR DNA fingerprinting at The Institute of Cancer Research (London, UK), were maintained in RPMI-1640 (Life Technologies Ltd). Antibodies: anti-EGFR (clone D38B1) anti-p-Y1173-EGFR (clone 53A5) were from Cell Signaling Technology Ltd.

#### *In vitro* liposomal transfection and imaging

A10 μM solution of the non-radiolabelled F1LA1 K_16_Cys-S-S-[PEG_6_-IPQA] lipopolyplex was applied to cells at 60% confluence in 96-well-plates. Cells expressing Picchu-X biosensor were imaged on customised “open” microscope automated FLIM system.^73^ Time-domain fluorescence lifetime images were acquired *via* time correlated single photon counting (TCSPC) at a resolution of 256 by 256 pixels, with 256 time bins and 100 frames accumulated over 300 seconds, *via* excitation and emission filters suitable for the detection of GFP fluorescence (Excitation filter: Semrock FF01-470/22 nm; Beam Splitter: Edmund 48NT-392 30R/70 T; Emission filter: Semrock FF01-510/20 nm). Conventional wide field fluorescence images were acquired with filter cubes for FITC (Excitation 480/30 nm, emission 535/45 nm) on a CCD camera (Hamamatsu 1394 ORCA-ERA), with an exposure time of typically 100–500 ms. FLIM analysis was performed with the TRI2 software (Version 2.7.8.9, Gray Institute, Oxford) as described previously.^74,75^

#### *In vivo* experiments

All animal studies were approved by the University College London Biological Services Ethical Review Committee and licensed under the UK Home Office regulations and the Guidance for the Operation of Animals (Scientific Procedures) Act 1986 (Home Office, London, United Kingdom). 12 female 6–8 weeks old CD1 nu/nu mice (Charles River Laboratories, UK) were injected subcutaneously in the right flank with 2 × 10^6^ LIM1215 cells in 100 μL PBS. Once palpable tumors were measure in three orthogonal directions using the following equation: length × height × width × π/6. Once tumors had reached approximately 70–100 mm^3^ mice were randomly divided into 2 groups; group 1 received an intravenous injection of 0.5Mbq MeO-[PEG_6_-[^125^I]-IPQA] **8** (*n* = 6), group 2 received an intravenous injection of 0.5MBq F1LA1 K_16_Cys-SMal-[PEG_3_-[^125^I]-IPQA]-lipopolyplex (*n* = 6). Mice were then culled at 3, 6 and 24 hours (*n* = 2 per time point) and all organs were taken, weighed and ^125^I uptake quantified using a gamma counter (Wizard, PerkinElmer) to obtain % Injected dose per gram (%ID g^−1^).

## Author contributions

Robin Bofinger, Gregory Weitsman, Rachel Evans, Matthias Glaser, Kerstin Sander and Tammy Kalber performed the experiments (chemical and peptide synthesis, cancer cell biology, FRET/FLIM, radiochemistry and biodistribution). Helen Allan, Robin Bofinger, Gregory Weitsman and Alethea Tabor wrote the initial draft, and all authors contributed to writing and reviewing the final draft. Erik Arstad, Daniel Hochhauser, Tammy Kalber, Helen Hailes, Tony Ng and Alethea Tabor supervised the research groups, and Tammy Kalber, Erik Arstad, Tony Ng and Alethea Tabor acquired the financial support for the research.

## Conflicts of interest

There are no conflicts to declare.

## Supplementary Material

NR-013-D1NR02770K-s001
