# Supplementary material for: Drug delivery, biodistribution and anti-EGFR activity: theragnostic nanoparticles for simultaneous in vivo delivery of tyrosine kinase inhibitors and kinase activity biosensors
Source: Nanoscale. 2021 Nov 3;13(44):18520–35. doi: 10.1039/d1nr02770k (PMC8601123; doi:10.1039/d1nr02770k)
Supplement: NR-013-D1NR02770K-s001 [file NR-013-D1NR02770K-s001.pdf]

Electronic Supplementary Material (ESI) for Nanoscale.
This journal is © The Royal Society of Chemistry 2021

Drug delivery, biodistribution and anti-EGFR activity: theragnostic nanoparticles for simultaneous *in vivo* delivery of tyrosine kinase inhibitors and kinase activity biosensors

Robin Bofinger, Gregory Weitsman, Rachel Evans, Matthias Glaser, Kerstin Sander, Helen Allan, Daniel Hochhauser, Tammy L. Kalber, Erik Årstad, Helen C. Hailes, Tony Ng* and Alethea B Tabor*

Supporting Information

Table of Contents

Optimization of EGFR inhibitor delivery to target cells 2
Figure S1. Effect of MeO-[PEG6-IPQA] (8) on EGFR activation by EGF in SW48 cells...2
Figure S2. Delivery of Picchu-X to DLD1 cells by lipopolylexes with different targeting peptides (P1 – P6 and K16). 3
Figure S3. Expression of Picchu-X in LIM1215 and DLD1 cells treated with drug containing F1LA1 lipopolyplex. 4
Figure S4. Expression of Picchu-X in LIM 1215 cells delivered with lipopolyplexes carrying different targeted peptides. 5
Figure S5. Comparison of expression of Picchu-X in LIM1215 and DiFi cells after transfection with lipopolyplex formulated with CLA1, NAE1 and NGE1. 6
Figure S6. Effect of different concentration of CLA lipopolyplexes on transfection efficiency of Picchu-X in LIM1215 cells. 7
Figure S7. Cleavage of peptide-TKI bioconjugate 1 under pseudo-intracellular conditions.8
General Synthetic Experimental 9
General Experimental for Peptide Synthesis 9
General Experimental for Radiolabelling Experiments 10
Synthesis of the known TKI intermediates 3 and 10, including modified reaction procedures 11
Experimental for the synthesis of 8 and [^{125}I]-8 precursor 13
^{1}H and ^{13}C NMR spectra: 2, 3, 4, 5, 6, 8, 10, 11, 12, 13, 14, 15, 16, S1, S2, S3, S4, S5, S6 ..16
HPLC and HRMS for P5, 1, 17 34
HPLC of Radiolabelled Compounds 8 and 9... 38
Characterisation of lipopolyplexes 39

Optimization of EGFR inhibitor delivery to target cells

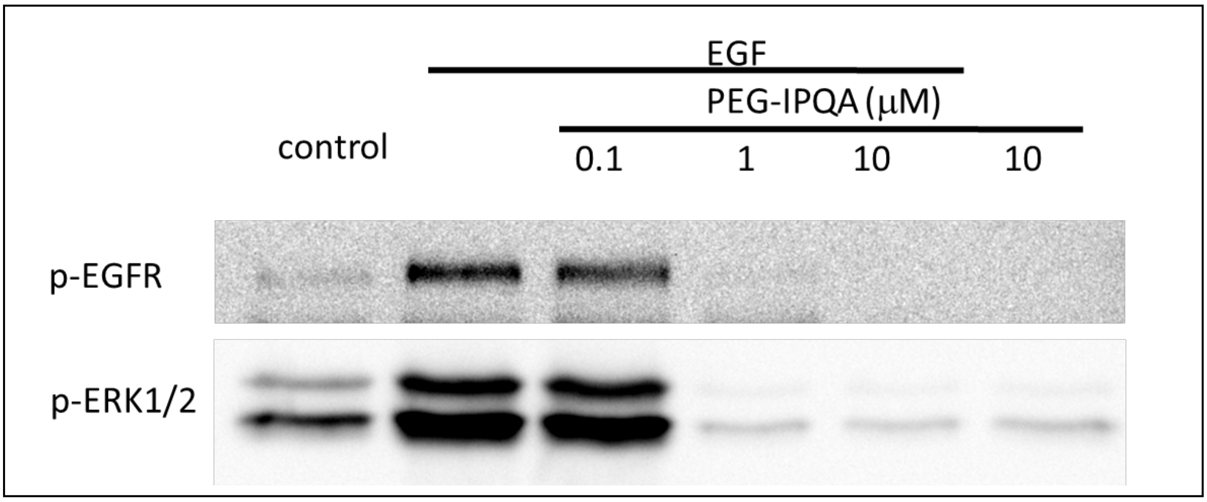

Figure S1. Effect of MeO-[PEG6-IPQA] (8) on EGFR activation by EGF in SW48 cells.

Cells were seeded in 6-well plate, 48 h later media was changed for media without serum. 24 h later cells were pre-treated with MeO-[PEG_6-IPQA] (**8**) at indicated concentrations for 1 h followed by treatment with EGF (100 ng/mL) for 5 min. Cells were lysed and subjected to western blot analysis for activation/phosphorylation of EGFR and downstream signalling molecule ERK1/2.

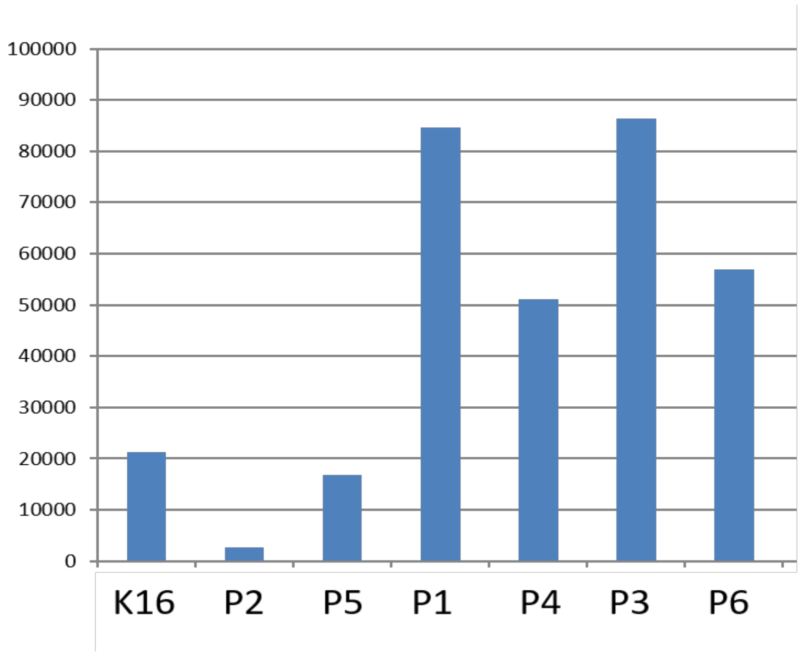

Figure S2. Delivery of Picchu-X to DLD1 cells by lipopolylexes with different targeting peptides (P1 – P6 and K16).

Cells were seeded in 96-well plate and treated with lipopolyplexes (10 μM) 24 h later. Expression of Picchu-X was measured by the number of expressing cells (red channel fluorescence) over the total number of cells in the culture (UV channel).

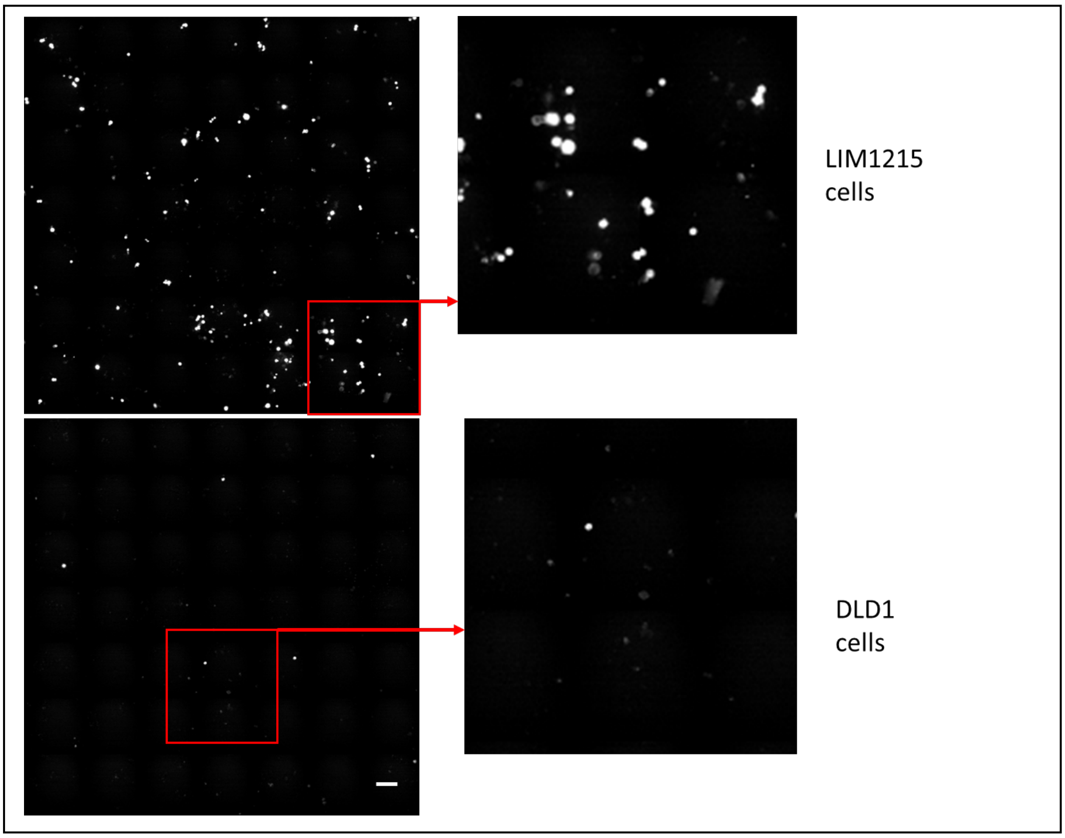

Figure S3. Expression of Picchu-X in LIM1215 and DLD1 cells treated with drug containing F1LA1 lipopolyplex.

Cells were seeded in 96-well plate, 24 h later lipopolyplexes were added at 10 mM concentration for 24 h. Cells were fixed and imaged on “open microscope” with 20x air objective (see materials and methods section). High resolution mosaic image (2 x 2 mm, consist of 49 individual images, scale bar – 150 mm) shows the expression pattern of Picchu-X in each cell line.

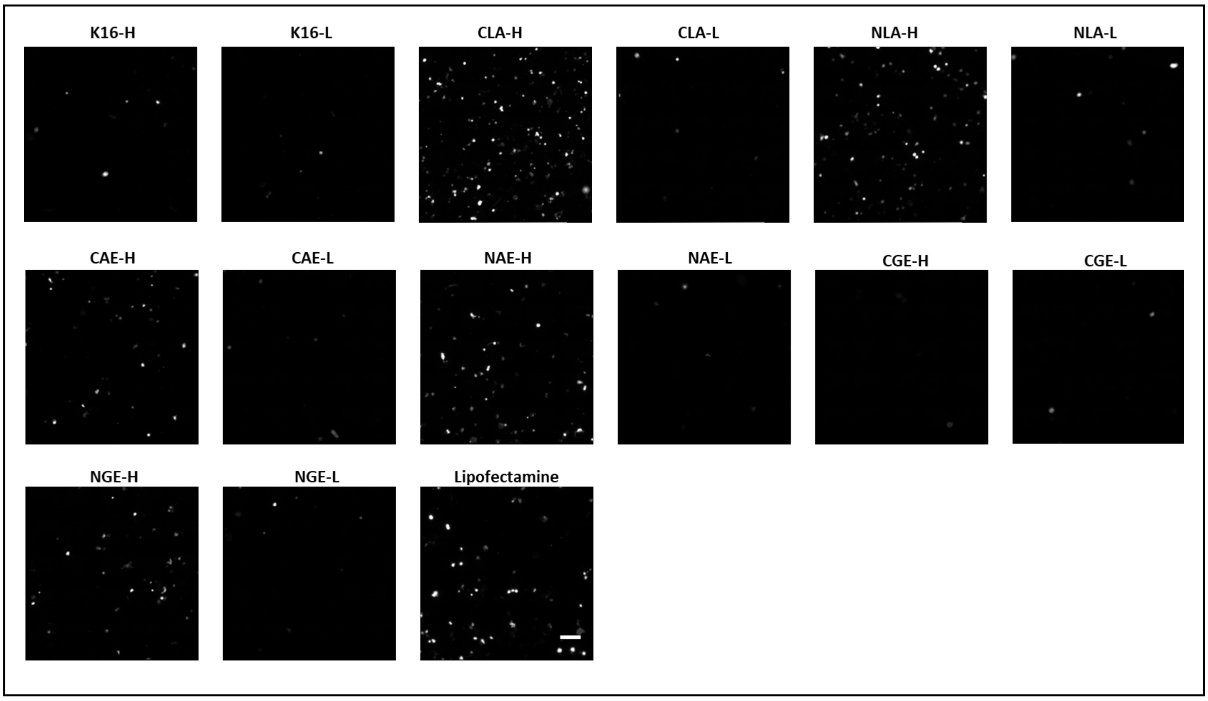

Figure S4. Expression of Picchu-X in LIM 1215 cells delivered with lipopolyplexes carrying different targeted peptides.

Stock concentration: 130 mM lipids, 10 µM peptide with 2 µg (L) and 4 µg (H) plasmid-DNA/100 µL lipososome/peptide solution. Cells were seeded in 96-well plate, 24 h later lipopolyplexes were added at 10 µM concentration for 24 h. Cells were fixed and imaged on "open microscope" with 20x air objective (see materials and methods section). High resolution mosaic image (2x2 mm, consist of 49 individual images, scale bar – 150 mm) shows the expression pattern in each cell culture depending on the lipopolyplex formulation. Bright dots are cells expressing Picchu-X (red channel).

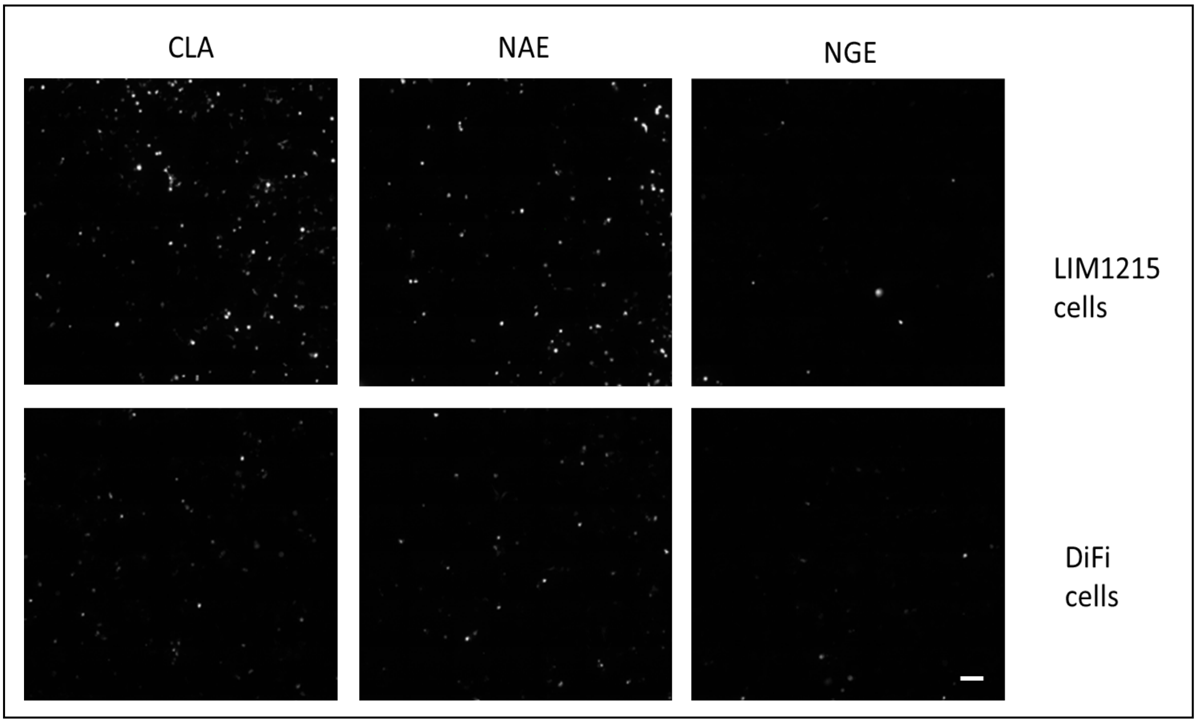

Figure S5. Comparison of expression of Picchu-X in LIM1215 and DiFi cells after transfection with lipopolyplex formulated with CLA1, NAE1 and NGE1.

Stock concentration: 130 mM lipids, 10 μM peptide (P1) 4 μg plasmid-DNA/100 μL liposome/peptide solution. Cells were seeded in 96-well plate, 24 h later lipopolyplexes were added at 10 μM concentration for 24 h. Cells were fixed and imaged on “open microscope” with 20x air objective (see materials and methods section). High resolution mosaic image (2 x 2 mm, consist of 49 individual images, scale bar – 150 mm) shows the expression pattern in each cell line depending on the lipopolyplex formulation. Bright dots are cells expressing Picchu-X (red channel).

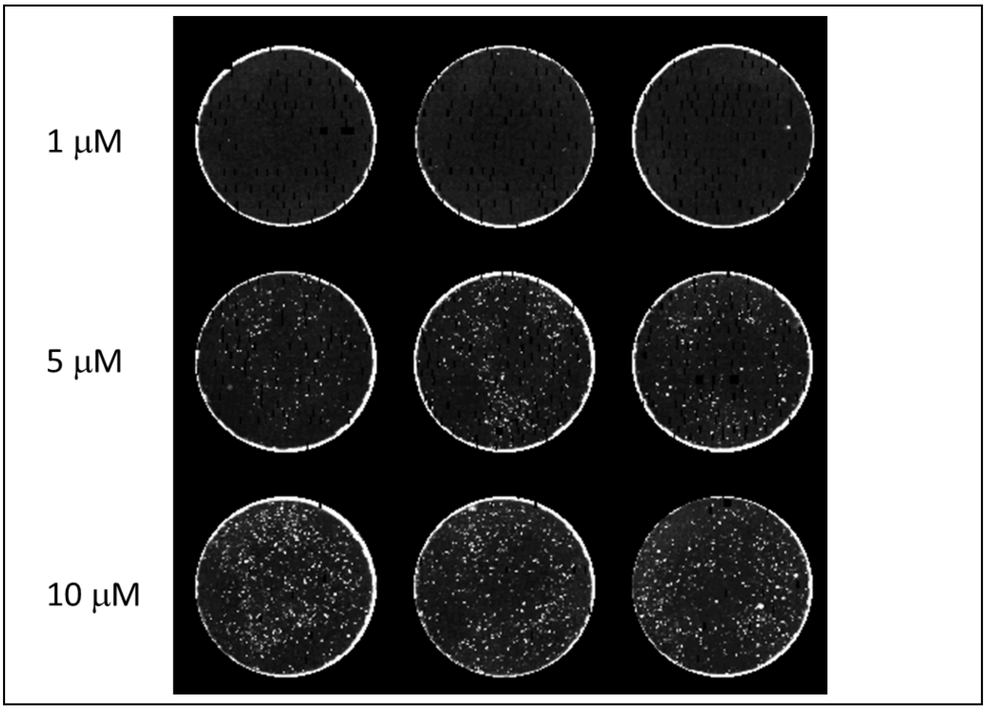

Figure S6. Effect of different concentration of CLA lipopolyplexes on transfection efficiency of Picchu-X in LIM1215 cells.

Stock concentration: 130 mM lipids, 10 μM peptide (P1) 4 μg plasmid-DNA/100 μL. Cells were seeded in 96-well plate, 24 h later indicated concentrations of lipopolyplexes (based on concentration of lipid) were added for 24 h. Low resolution mosaic image of Picchu-X expressing cells (green fluorescence signal) in the whole well was obtained on "open microscope" (see materials and methods section, 3 wells per group are shown). Bright dots are cells expressing Picchu-X (red channel).

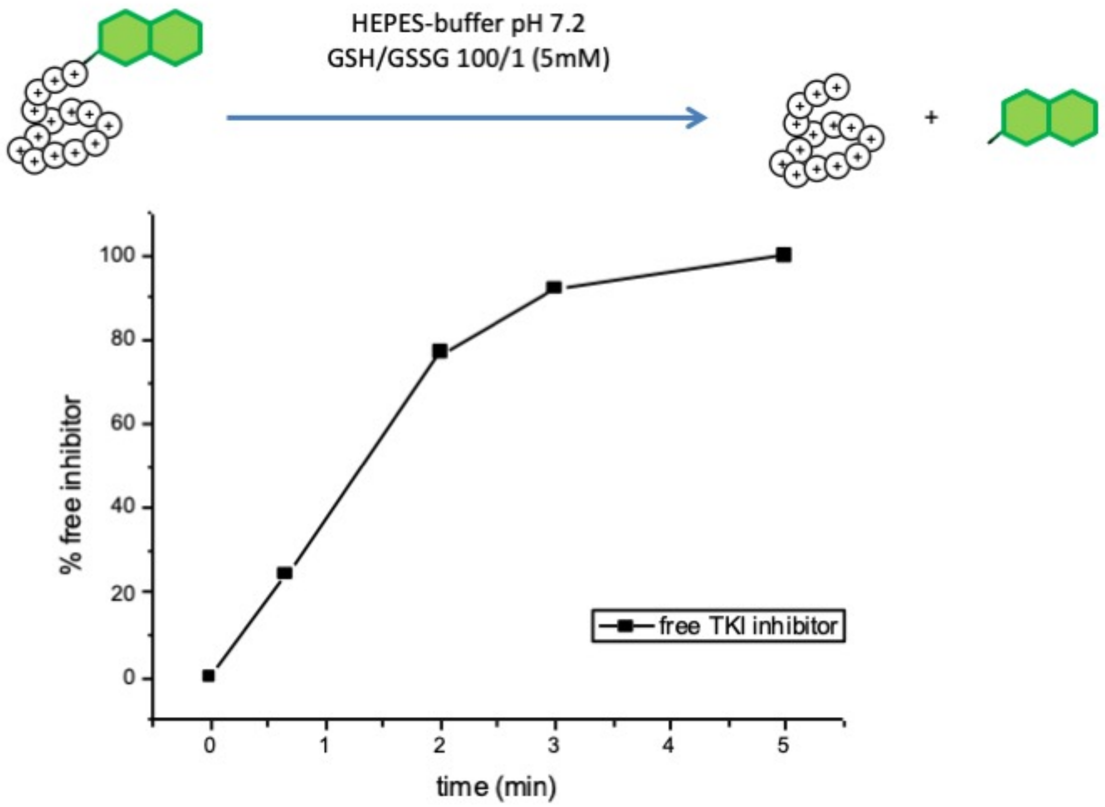

Figure S7. Cleavage of peptide-TKI bioconjugate 1 under pseudo-intracellular conditions. Release of the TKI was followed by HPLC.

General Synthetic Experimental

Reagents for chemical synthesis were purchased from Sigma-Aldrich Co. Ltd. unless otherwise stated and used without further purification. All reagents were of commercial quality and used as received and all solvents anhydrous. Thin Layer Chromatography (TLC) was performed on aluminium backed Sigma-Aldrich TLC plates with F254 fluorescent indicator. Visualisation was performed by quenching of UV fluorescence or by staining the plates with potassium permanganate solution (1.5 g $KMnO_4$, 10 g K_2CO_3, 1.25 mL 10% NaOH in 200 mL water), phosphomolybdic acid solution (10 % w/w in ethanol). Normal phase flash chromatography was carried out using silica gel (43–60 µm) supplied by Merck. An automated column system (TELEDYNE Isco, Combi Flash Companion) was used where specified (GraceResolv Cartridge (12g)). ^{1}H and ^{13}C NMR spectra were recorded on a Bruker Avance III 600, 500 or 300 instrument, as stated, at the field indicated. Chemical shifts (in ppm) were referenced to residual protonated solvent. Coupling constants (*J*) were measured in Hertz (Hz), multiplicities for ^{1}H coupling are shown as s (singlet), d (doublet), t (triplet), m (multiplet), or a combination of the above. Deuterated chloroform ($CDCl_3$) was used as a solvent. ESI-MS analysis was performed on an Agilent 6510 QTOF mass spectrometer. EI-MS analysis was performed on a Thermo Finingan MAT900 magnetic sector mass spectrometer.

General Experimental for Peptide Synthesis

All peptides were synthesized on a MultiSynTech Syro I automated system. Pre-loaded Fmoc-Lys(Boc)-NovaSyn TGT resin (0.2 mmolg-1, 100 mg, 21 µmol) with standard HBTU/DIPEA coupling chemistry was used. All resins were pre-swelled in DMF for 10 min prior to the start of the synthesis. The total volume of all reagents in each step was 1.5 mL. *N*-Fmoc-protected amino acids were purchased from Novobiochem. Where required, the following side-chain protected amino acids were used: Fmoc-Lys(Boc)-OH; Fmoc-Arg(Pbf)-OH; Fmoc-Tyr(tBu)-OH; Fmoc-His(Trt)-OH; Fmoc-Thr(tBu)-OH.

Fmoc removal: Each deprotection step was allowed to proceed for 3 min agitating for 20 s every minute in 40% piperidine in DMF at room temperature. The reagents were removed by filtration under vacuum and the resin washed with DMF (4 x 1.5 mL). The deprotection step was repeated using 20% piperidine in DMF with agitation for 20 s every minute for 10 min. The reagents were removed by filtration under vacuum and the resin washed with DMF (6 x 1.5 mL).

Amino acid coupling: To the resin was added 4 eq of the amino acid, 4 eq of HBTU and 8 eq of DIPEA to a total volume of 1.5 mL. The mixture was agitated for 20 s every 3 min for a total of 40 min at room temperature. The reagents were removed by filtration and the resin washed with DMF (4 x 1.5 mL).

Peptide Cleavage: After completion of the synthesis the peptide sequence was deprotected and cleaved from the resin by incubation in 2.5 mL of a cleavage mixture containing TFA/TES/EDT/H_2O (9400:250:100:250 µL) for 3 h at room temperature. The cleavage cocktail was drained into a 15 mL Falcon tube filled with diethyl ether and the peptide allowed to precipitate at -20 °C for 10 min. The suspension was centrifuged at 4000 rpm for 5 min, the supernatant was discarded and the pellet re-dissolved in diethyl ether. This purification process was repeated three times after which the precipitate was dissolved in 0.1% TFA containing HPLC grade water, frozen in liquid N_2 and freeze-dried overnight.

Purification: Preparative HPLC was performed on a Varian ProStar HPLC system with a Model 210 solvent delivery module and a Model 320 UV detector using a Varian column (21.2 x 100 mm, C18, 5 µm beads, flow rate of 10 $mLmin^{-1}$) or a Gemini column (4.6 x 250 mm, C18, 5 µm beads, flow rate of 4 $mLmin^{-1}$). The analysis of the chromatograms was conducted using Star Chromatography Workstation software Version 1.9.3.2. Analytical HPLC was performed using a Varian column (2.1 x 250 mm, C18, 5 µm beads, flow rate of 1 mL min^{-1}). A solvent system of water (0.1% TFA) as solvent A and acetonitrile (0.1% TFA) as solvent B was used with a gradient of 5% B: 0 to 2 min, 5% to 95% B: 2 to 19 min; 95% to 5% B: 19 to 20 min then 5% B 20 to 23 min. Fractions containing the correct peak were pooled, the solvent removed under reduced pressure to < 5 mL, and the solution freeze-dried overnight. The resulting crystalline powder was analyzed *via* HPLC and ESI-MS.

General Experimental for Radiolabelling Experiments

All labelling reactions were performed using radioiodine starting material [^{125}I]NaI purchased from Perkin Elmer LAS (UK) Ltd (#NEZ033A020MC). Radioactivity measurements were done using a calibrated ion chamber (PTW Curiementor 4). Unless specifically mentioned, chemicals were purchased from Fisher Scientific UK, VWR, or Sigma-Aldrich UK and used without purification. Radio-HPLC injections were run on an Agilent 1200 HPLC system equipped with a 1200 Series Diode Array Detector and a GABI Star NaI(TI) scintillation detector (energy window 40-100 keV). The system was used both for purification and characterization of radiotracers. Columns and conditions used for purification and quality controls (QC) are indicated in the protocol or next to the corresponding chromatogram.

Radiochemical yields were calculated as follows:

- Analytical radiochemical yields were determined using radio-HPLC chromatograms of the quenched crude labeling mixture and refer to the integrated radioactivity peak areas.
- Isolated radiochemical yields (RCY) refer to the activity of the HPLC purified tracer after SepPak cartridge formulation. The product activity is divided by the starting

activity of [^{125}I]NaI. No corrections were made for losses during transfer and cartridge formulation.

Synthesis of the known TKI intermediates 3 and 10, including modified reaction procedures

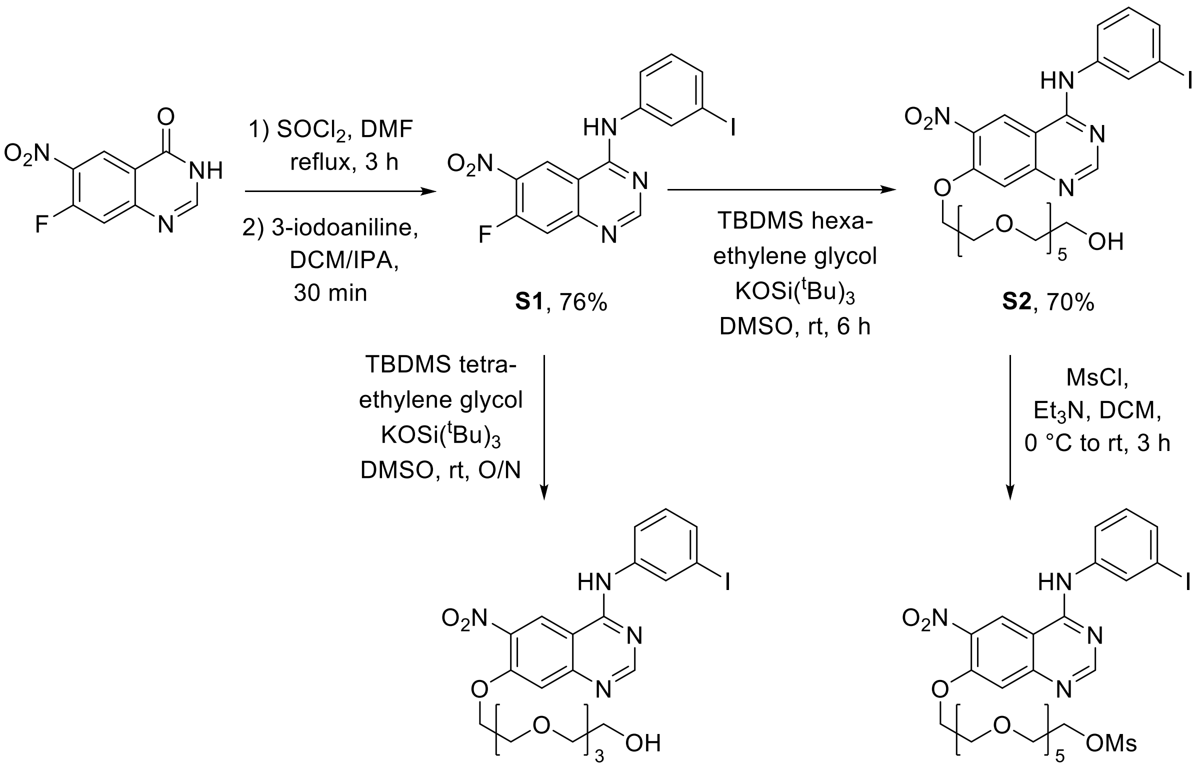

10, 78%

3, 98%

7-Fluoro-*N*-(3-iodophenyl)-6-nitroquinazolin-4-amine (S1).[2] **S1** was synthesised using a modified work up procedure.[2] 7-fluoro-6-nitroquinazolin-4(3H)-one[1] (1.00 g, 4.78 mmol) was dispersed in $SOCl_2$ (20 mL) and 3 drops of DMF were added. The reaction mixture was heated under reflux for 3 h. The now clear solution was cooled to room temperature and $SOCl_2$ was removed *in vacuo*. The crude material was dissolved in CH_2Cl_2 (10 mL) and a solution of 3-iodoaniline (1.15 g, 5.25 mmol) in isopropanol (25 mL) was added. The reaction was stirred for 30 min and a precipitate formed. Petroleum ether was added to ensure full precipitation of the product. The precipitate was filtered, redissolved in methanol (200 mL) and neutralised with Et_3N upon which the product precipitated out again. H_2O was added to ensure full precipitation. The product was collected by filtration and dried under high vacuum to give **S1** (1.50 g, 76%; Lit. yield[2] 45%) as a yellow solid. mp 214-216 °C (Lit.[2] 160-161 °C); ^{1}H NMR data ($CDCl_3$) agreed with that reported in the literature.[2]

4-(3-Iodophenylamino)-6-nitro-7-(PEG_6)-quinazoline (S2).[3] **S2** was synthesised using a modified work up procedure.[3] To a solution of **S1** (600 mg, 1.50 mmol) and *tert*-butyldimethylsilyl hexaethylene glycol (1.00 g, 2.50 mmol) in DMSO (40 mL) was added potassium tert-butylsilanolate (630 mg, 4.90 mmol) and the now deep crimson mixture stirred at rt for 6 h. Saturated aqueous $NaHCO_3$ solution was added and the organic phase extracted (3 x EtOAc). The combined organic phases were washed with 3 x saturated NaCl solution, dried ($MgSO_4$), filtered and concentrated *in vacuo*. Purification was achieved using flash column chromatography (2-5% MeOH/CH_2Cl_2) to give **S2** (690 mg, 70%) as a yellow oil. LRMS agreed with that reported in the literature.[3] ^{1}H NMR run in $CDCl_3$, literature data in DMSO. ^{1}H NMR (300 MHz, $CDCl_3$) δ 8.76 (s, 1H), 8.72 (s, 1H), 8.25 (t, J = 1.9 Hz, 1H), 8.24 (br s, 1H), 7.78-7.81 (m, 1H), 7.51-7.54 (m, 1H), 7.32 (s, 1H), 7.15 (t, J = 8.1 Hz, 1H), 4.33-4.36 (m, 2H), 3.92-3.95 (m, 2H) 3.52 - 3.77 (m, 20 H) ppm.

17-((4-((3-Iodophenyl)amino)-6-nitroquinazolin-7-yl)oxy)-3,6,9,12,15-pentaoxaheptadecyl methanesulfonate (3).[3] The title compound was synthesised according to the literature procedure[3] to give **3** (480 mg, 98%; Lit. yield[3] 67%) as a yellow oil. LRMS agreed with that reported in the literature.[3] ^{1}H NMR run in $CDCl_3$, literature data in DMSO. ^{1}H NMR (300 MHz, $CDCl_3$) δ 8.77 (s, 1H) 8.67 (s, 1H), 8.26 (t, J = 1.9 Hz, 1H), 7.99 (br s, 1H), 7.77-7.80 (m, 1H), 7.52-7.56 (m, 1H), 7.37 (s, 1H), 7.19 (t, J = 8.1 Hz, 1H), 4.34-4.40 (m, 4H), 3.93-3.96 (m, 2H), 3.35-3.77 (m, 18H), 3.08 (s, 3H) ppm.

2-(2-(2-(2-((4-((3-Iodophenyl)amino)-6-nitroquinazolin-7-yl)oxy)ethoxy)ethoxy)ethoxy)ethanol (10).[2] **10** was synthesised using a modified literature procedure.[2] To a solution of **S1** (500 mg, 1.21 mmol) and *tert*-butyldimethylsilyl tetraethylene glycol (643 mg, 2.08 mmol) in DMSO (30 mL) was added potassium *tert*-butylsilanolate (525 mg, 4.08 mmol). The reaction mixture was stirred at rt overnight, poured onto ice cooled water and extracted (3 x EtOAc). The combined organic phases were washed with brine, H_2O, dried ($MgSO_4$), filtered and concentrated *in vacuo*. Purification was achieved using flash column chromatography (3-5% MeOH/CH_2Cl_2) to give **10** (550 mg, 78%; Lit. yield[2] 48%) as a yellow oil. ^{1}H NMR data ($CDCl_3$) agreed with that reported in the literature.[2]

Experimental for the synthesis of 8 and [^{125}I]-8 precursor

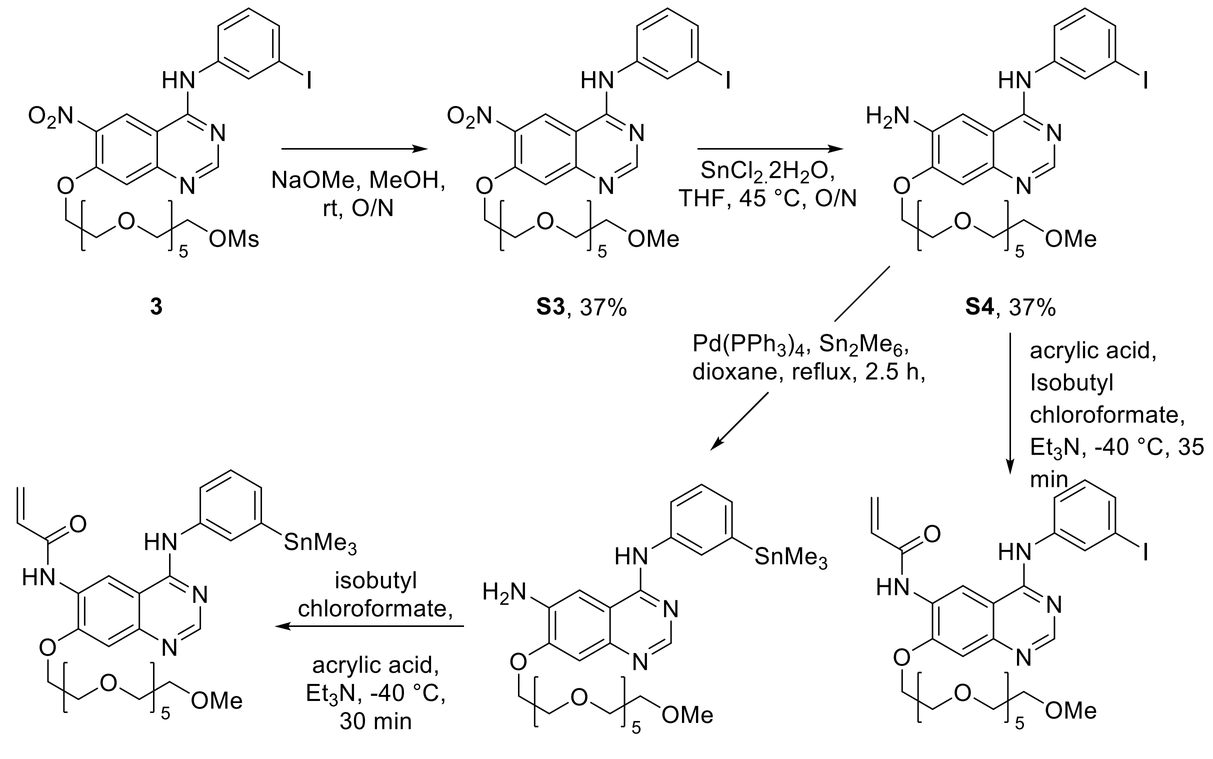

3 **S3**, 37% **S4**, 37% **S6** **S5** **8**, 27%

7-(2,5,8,11,14,17-Hexaoxanonadecan-19-yloxy)-N-(3-iodophenyl)-6-nitroquinazolin-4-amine (S3). A solution of **3** (300 mg, 0.416 mmol) in MeOH (5 mL) was treated with NaOMe solution (25 % in MeOH, 900 µL, 3.94 mmol) and the reaction mixture stirred at rt overnight. After this time, the reaction mixture was diluted with CH_2Cl_2 and the organic phase washed with saturated aqueous $NaHCO_3$ solution and the organic phase dried ($MgSO_4$), filtered and concentrated *in vacuo*. Purification was achieved using flash column chromatography (2% MeOH/CH_2Cl_2) to give **S3** (104 mg, 37%) as a yellow oil. ^{1}H NMR (600 MHz, $CDCl_3$) δ 8.87 (s, 1H), 8.82 (s, 1H), 8.73 (br s, 1H), 8.28 (t, *J* = 1.7 Hz, 1H), 7.85 (dd, *J* = 8.2, 1.3 Hz, 1H), 7.47 (d, *J* = 7.9 Hz, 1H), 7.10-7.13 (m, 2H), 4.17-4.18 (m, 2H), 3.84-3.86 (m, 2H), 3.65-3.73 (m, 4H), 3.55-3.60 (m, 6H), 3.47-3.52 (m, 6H), 3.41-3.44 (m, 4H), 3.26 (s, 3H) ppm; ^{13}C NMR (150 MHz, $CDCl_3$) δ 158.2, 158.0, 154.7, 153.6, 139.7, 139.2, 133.6, 131.0, 130.4, 122.2, 121.5, 110.4, 108.5, 94.1, 71.9, 71.3, 70.8, 70.61, 70.59, 70.6, 70.5, 70.4, 69.7, 69.2, 60.0 ppm (2 OCH_2 peaks overlapping); HRMS (ESI) *m/z*: [M+H]+ Calcd for $C_{27}H_{36}IN_4O_9$ 687.1587; Found 687.0877.

7-(2,5,8,11,14,17-Hexaoxanonadecan-19-yloxy)-N4-(3-iodophenyl)quinazoline-4,6-diamine (S4). A solution of **S3** (100 mg, 0.146 mmol) in THF (3 mL) was treated with $SnCl_2$*$2H_2O$ (98 mg, 20.4 mmol). The reaction mixture was heated to 45 °C overnight. After this time another portion of $SnCl_2$*$2H_2O$ (329 mg, 1.46 mmol, 10 eq.) was added and the

reaction heated to 60 °C for 1 h. 1 N HCl was added and the aqueous phase was extracted (3 x CH_2Cl_2). The aqueous phase was neutralized and extracted (2 x CH_2Cl_2). The combined organic phases were dried ($MgSO_4$), filtered and concentrated *in vacuo*. Purification was achieved using flash column chromatography (2% MeOH/CH_2Cl_2) to give **S4** (35 mg, 37%) as a yellow oil. ^1H NMR (500 MHz, $CDCl_3$) δ 8.56 (s, 1H), 8.20 (t, *J* = 1.7 Hz, 1H), 7.82 (br s, 1H), 7.78-7.80 (m, 1H), 7.40-7.42 (m, 1H), 7.32 (s, 1H), 7.14 (s, 1H), 7.09 (t, *J* = 8.0 Hz, 1H), 4.72 (br s, 2H), 4.27-4.29 (m, 2H), 3.92-3.94 (m, 2H), 3.55 - 3.77 (m, 20H), 3.26 (s, 3 H) ppm; ^{13}C NMR (125 MHz, $CDCl_3$) δ 155.4, 152.6, 151.4, 145.1, 140.8, 138.8, 132.2, 130.4, 129.7, 120.2, 110.7, 107.3, 101.4, 94.2, 72.0, 71.0, 70.79, 70.75, 70.71, 70.69, 70.63, 70.62, 70.56, 69.1, 68.3, 58.8 ppm (1 OCH_2 peaks overlapping); HRMS (ESI) *m/z*: [M+H]+ Calcd for $C_{27}H_{37}IN_4O_7$ 656.1707; Found 656.1707.

N-(7-(2,5,8,11,14,17-Hexaoxanonadecan-19-yloxy)-4-((3-iodophenyl)amino)quinazolin-6-yl)acrylamide (8). A solution of acrylic acid (12.7 µL, 0.186 mmol) in THF (1 mL) was cooled to 0 °C and treated with isobutyl chloroformate (19.1 µL, 0.148 mmol) followed by dropwise addition of Et_3N (29 µL, 0.209 mmol). The cloudy mixture was cooled to -40 °C using a acetonitrile/dry ice bath and **S4** (35 mg, 0.054 mmol) in THF (1 mL) was added in one portion. The reaction was stirred at -40 °C for 35 min, then saturated aqueous $NaHCO_3$ solution was added and the aqueous phase extracted (3 x CH_2Cl_2). The combined organic phases were dried (MgSO4), filtered and concentrated in vacuo. Purification was achieved using flash column chromatography (0-2% MeOH/CH_2Cl_2) to give **8** (10 mg, 27%) as a yellow oil. ^1H NMR (600 MHz, $CDCl_3$) δ 9.16 (s, 1H), 8.90 (br s, 1H), 8.67 (s, 1H), 8.17 (s, 1H), 7.73-7.74 (m, 1H), 7.44-7.54 (m, 2H), 7.14 (t, *J* = 7.9 Hz, 1H), 6.50-6.51 (m, 2H), 5.86 (dd, *J* = 6.3, 5.3 Hz, 1H), 4.40-4.41 (m, 2H), 3.96-4.00 (m, 2H), 3.52-3.77 (m, 20H), 3.37 (s, 3H); ^{13}C NMR (150 MHz, CDCl3) δ 164.4, 157.2, 153.1, 131.2, 130.9, 130.5, 128.9, 128.5, 121.5, 110.7, 109.1, 94.1, 72.0, 70.8, 70.7, 70.60, 70.57, 69.1, 69.0, 59.2, 1.2 ppm (some peaks missing due to poor signal to noise); HRMS (ESI) *m/z*: [M+H]+ Calcd for $C_{30}H_{39}IN_4O_8$ 711.1885 Found 711.1893.

***N*-(7-((2,5,8,11,14,17-Hexaoxanonadecan-19-yl)oxy)-4-((3-(trimethylstannyl)phenyl)amino)quinazolin-6-yl)acrylamide (S5).** Compound **S5** was synthesised in an analogous manner to that of compound **13** utilising **S4** in place of **12** to give **S5** as a yellow oil. ^1H NMR (600 MHz, $CDCl_3$) δ 8.54 (s, 1H), 7.84-7.86 (m, 1H), 7.66-7.76 (m, 1H), 7.68 (br s, 1H), 7.34-7.39 (m, 1H), 7.28 (s, 1H), 7.17-7.28 (m, 1H), 7.13 (s, 1H), 4.27-4.29 (m, 2H), 3.93-3.94 (m, 2H), 3.75-3.75 (m, 2H), 3.61-3.70 (m, 16H), 3.55-3.56 (m, 2H), 3.28 (s, 3H), 0.30 (s, 9H) ppm; ^{13}C NMR (125 MHz, $CDCl_3$) δ 155.9, 152.4, 152.0, 145.3, 143.1, 139.0, 138.5, 131.3, 128.6, 128.5, 121.7, 110.8, 107.4, 101.6, 72.0, 71.0, 70.80, 70.76,

70.72, 70.66, 70.6, 69.2, 68.2, 58.9, -9.3 ppm (3 OCH_2 peaks overlapping); LRMS (ESI) *m/z*: [M+H]+ ($C_{30}H_{47}N_4O_7Sn$) 695.

7-((2,5,8,11,14,17-hexaoxanonadecan-19-yl)oxy)-*N*^{4}-(3-(trimethylstannyl)phenyl)quinazoline-4,6-diamine (S6). Compound **S6** was synthesised in an analogous manner to that of compound **14** utilising **S5** in place of **13** to give **S6** as a yellow oil. ^{1}H NMR (600 MHz, $CDCl_3$) δ 9.14 (s, 1H), 8.98 (br s, 1H), 7.83-7.85 (m, 1H), 7.78 (br s, 1H), 7.78 (d, *J* = 2.0 Hz, 1H), 7.40 (t, *J* = 7.7 Hz, 1H), 7.27-7.29 (m, 2H), 6.48-6.56 (m, 2H), 5.83 (dd, *J* = 9.2, 2.1 Hz, 1H), 4.37-4.39 (m, 2H), 3.98-3.99 (m, 2H), 3.60-3.77 (m, 18H), 3.52-3.54 (m, 2H), 3.36 (s, 3H), 0.33 (s, 9H) ppm; ^{13}C NMR (150 MHz, $CDCl_3$) δ 164.4, 157.3, 154.5, 152.5, 147.7, 143.3, 137.9, 132.1, 131.4, 129.4, 128.5, 128.35, 128.30, 122.7, 110.6, 109.6, 107.6, 72.0, 70.8, 70.7, 70.63, 70.61, 70.60, 69.1, 68.6, 59.1, -9.3 ppm (4 OCH_2 peaks overlapping); HRMS (ESI) m/z: [M+H]+ Calcd for $C_{33}H_{49}N_4O_8Sn$ 749.2572; Found 749.2592.

^{1}H and ^{13}C NMR spectra: 2, 3, 4, 5, 6, 8, 10, 11, 12, 13, 14, 15, 16, S1, S2, S3, S4, S5, S6

Compound 2

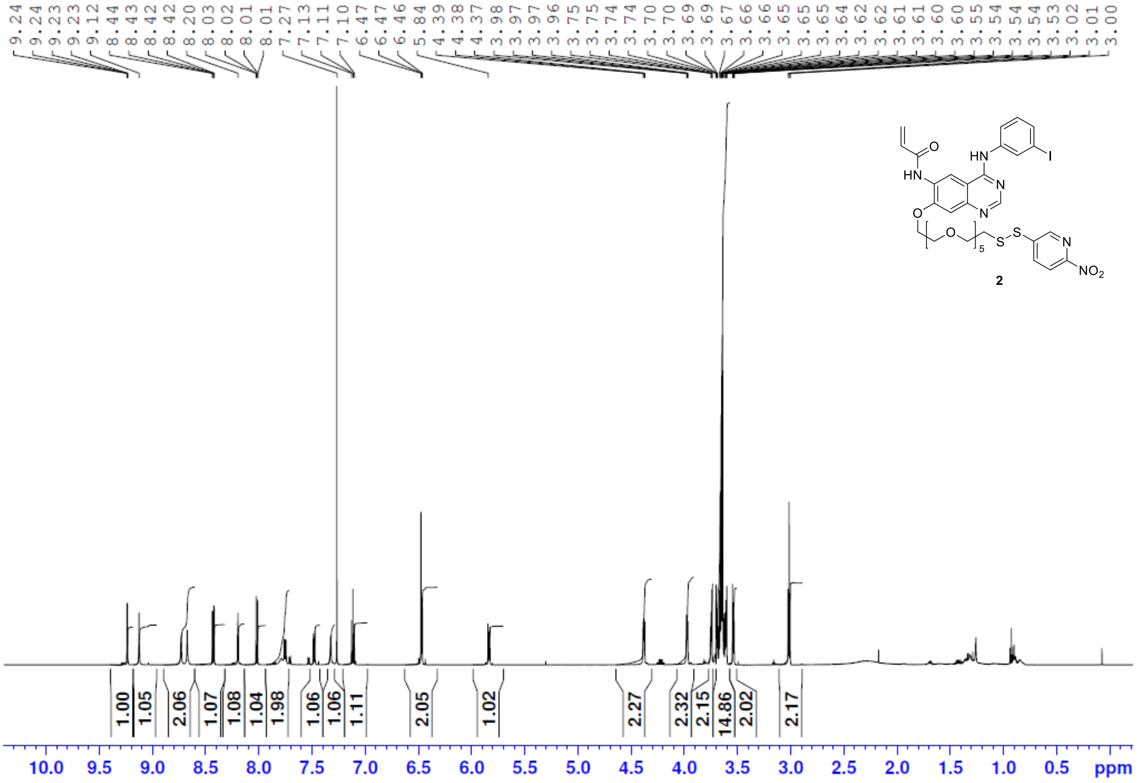

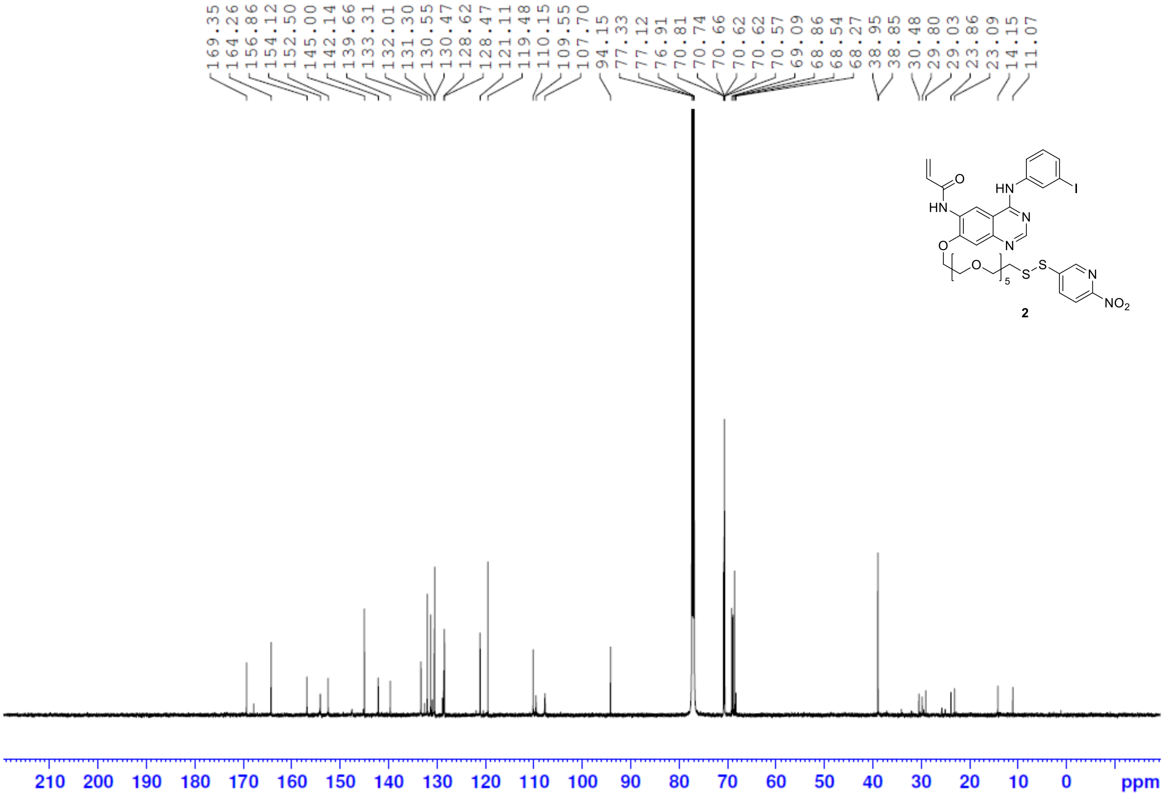

Compound 3

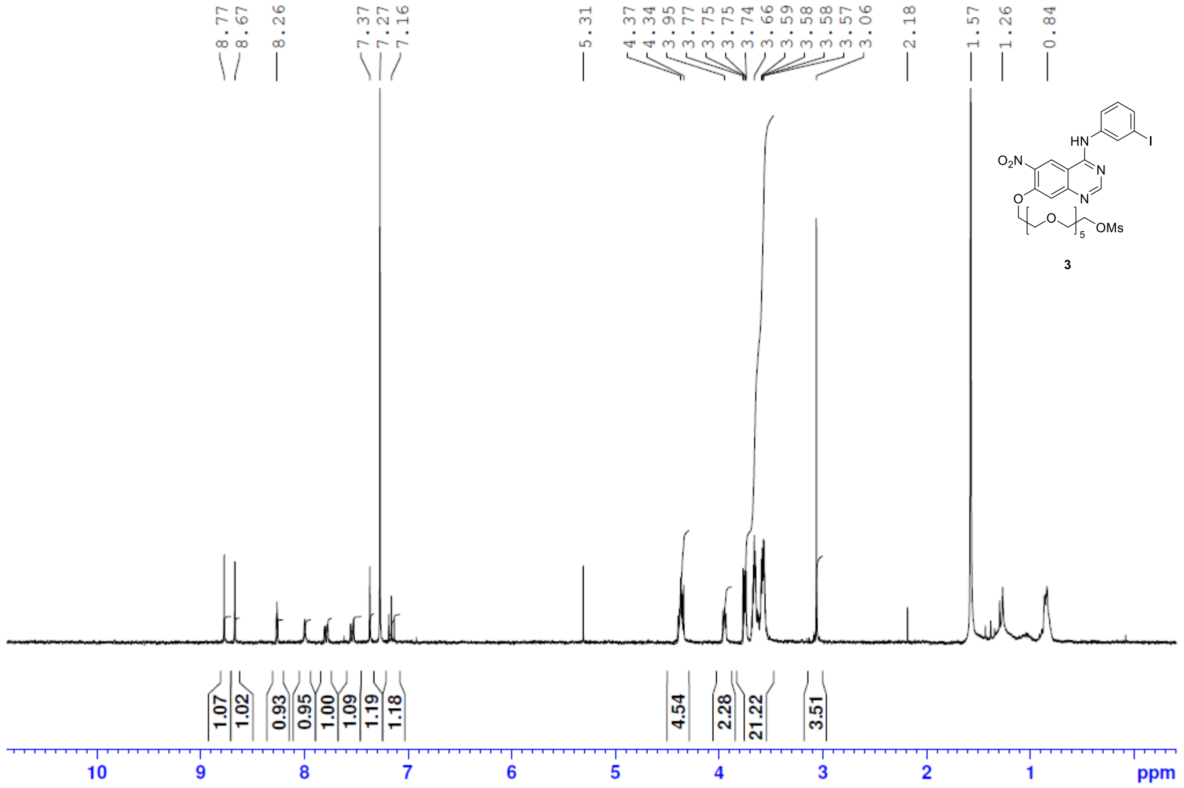
8.77
8.67
8.26
7.37
7.27
7.16
5.31
4.37
4.34
3.95
3.77
3.75
3.75
3.74
3.66
3.59
3.58
3.58
3.57
3.06
2.18
1.57
1.26
0.84
HN
I
O2N
N
N
O
O
5
OMs
3
1.07
1.02
0.93
0.95
1.00
1.09
1.19
1.18
4.54
2.28
21.22
3.51
10
9
8
7
6
5
4
3
2
1
ppm

Compound 4

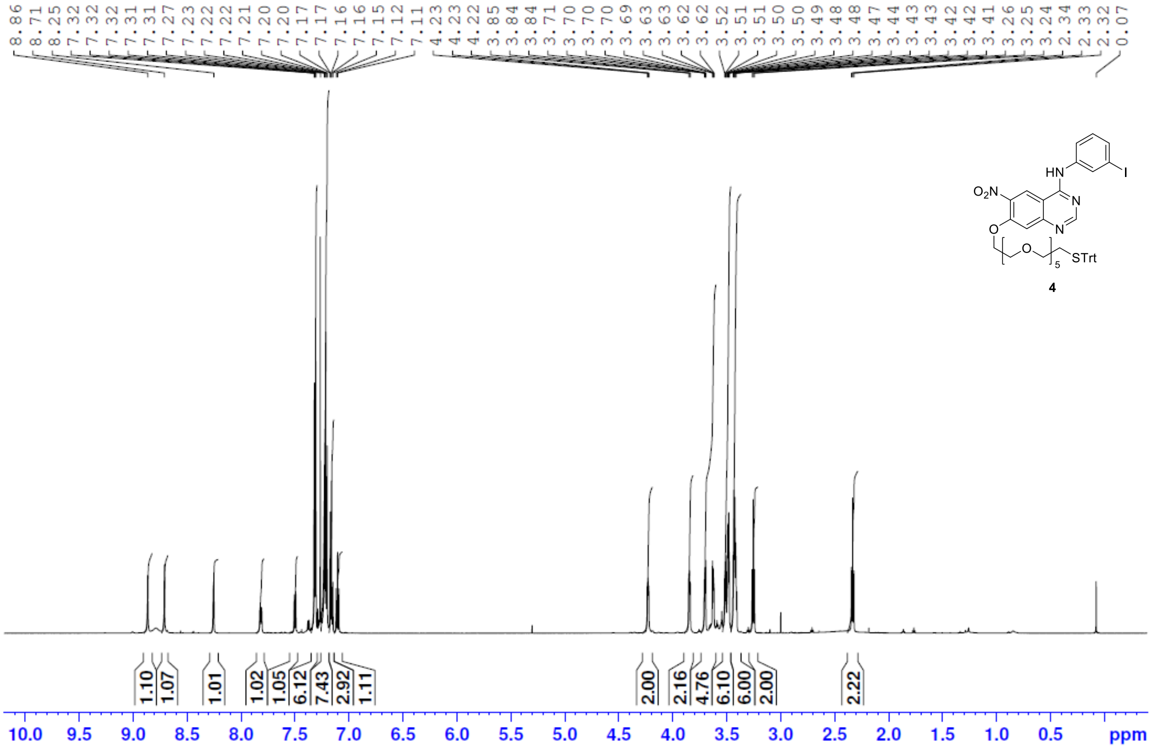

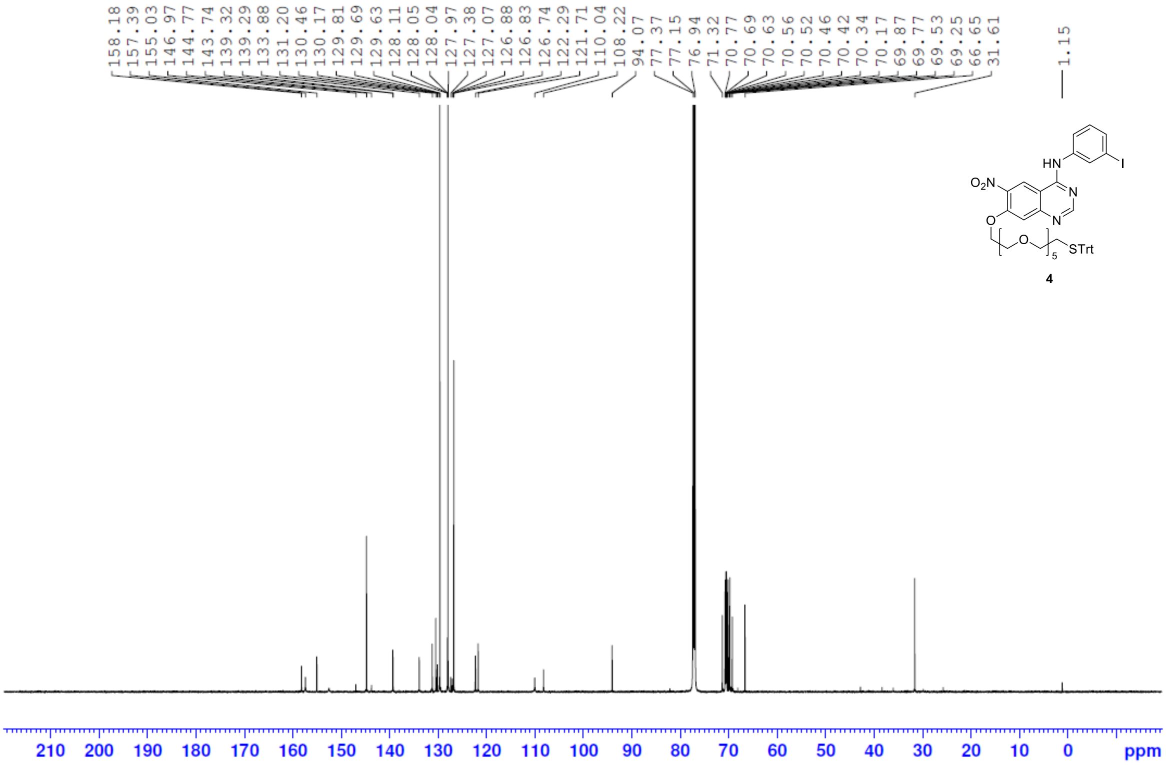

Compound 5

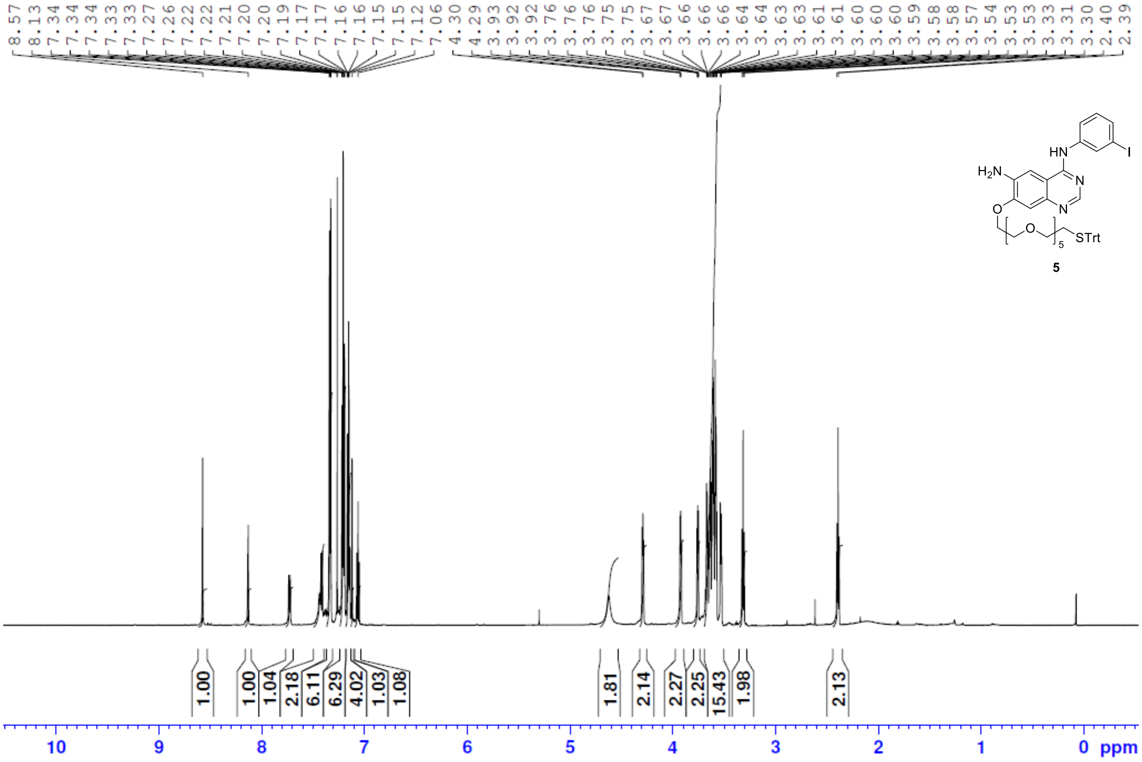
8.57
8.13
7.34
7.34
7.33
7.33
7.27
7.26
7.22
7.21
7.20
7.19
7.17
7.17
7.16
7.16
7.15
7.15
7.12
7.06
4.30
4.29
3.93
3.92
3.76
3.76
3.75
3.75
3.67
3.67
3.66
3.66
3.66
3.64
3.64
3.63
3.63
3.61
3.61
3.60
3.60
3.60
3.59
3.58
3.58
3.57
3.54
3.53
3.53
3.33
3.31
3.30
2.40
2.39
HN
I
H_2N
N
N
O
O
5
STrt
5
1.00
1.00
1.04
2.18
6.11
6.29
4.02
1.03
1.08
1.81
2.14
2.27
2.25
15.43
1.98
2.13
10 9 8 7 6 5 4 3 2 1 0 ppm

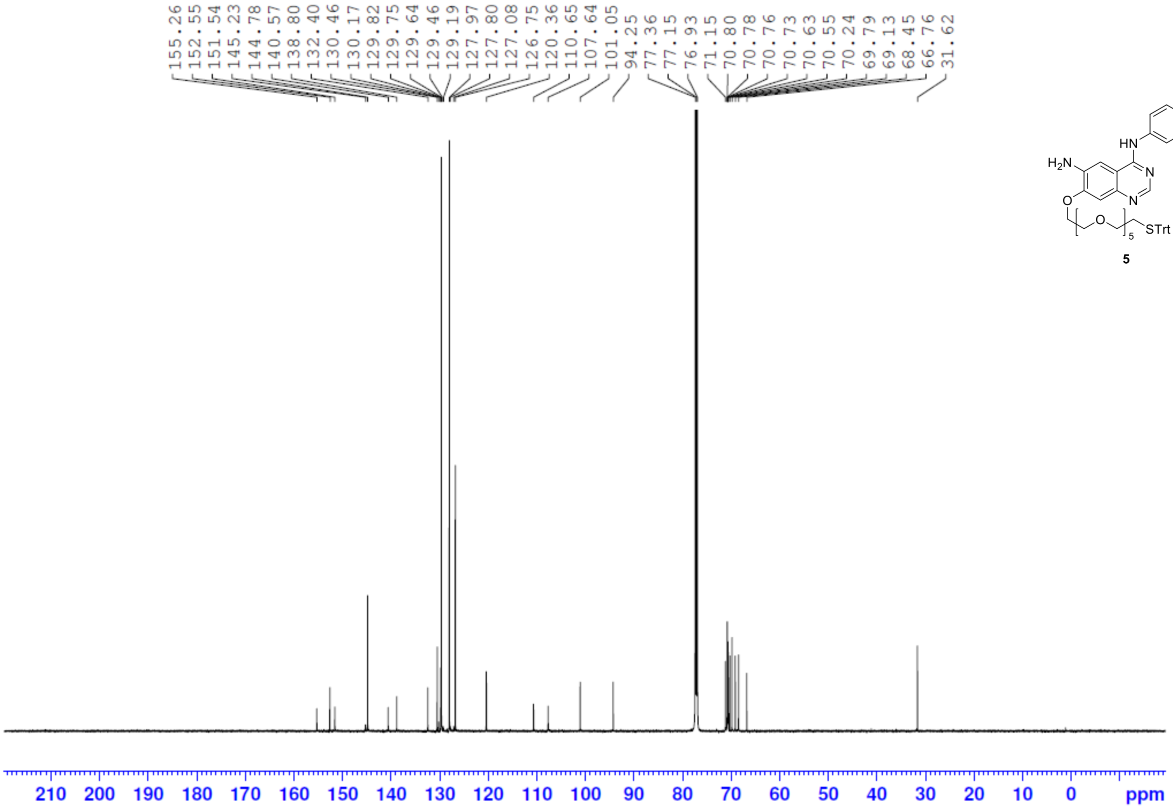
155.26
152.55
151.54
145.23
144.78
140.57
138.80
132.40
130.46
130.17
129.82
129.75
129.64
129.46
129.19
127.97
127.80
127.08
126.75
120.36
110.65
107.64
101.05
94.25
77.36
77.15
76.93
71.15
70.80
70.78
70.76
70.73
70.63
70.55
70.24
69.79
69.13
68.45
66.76
31.62
HN
I
H_2N
N
N
O
O
5
STrt
5
210 200 190 180 170 160 150 140 130 120 110 100 90 80 70 60 50 40 30 20 10 0 ppm

Compound 6

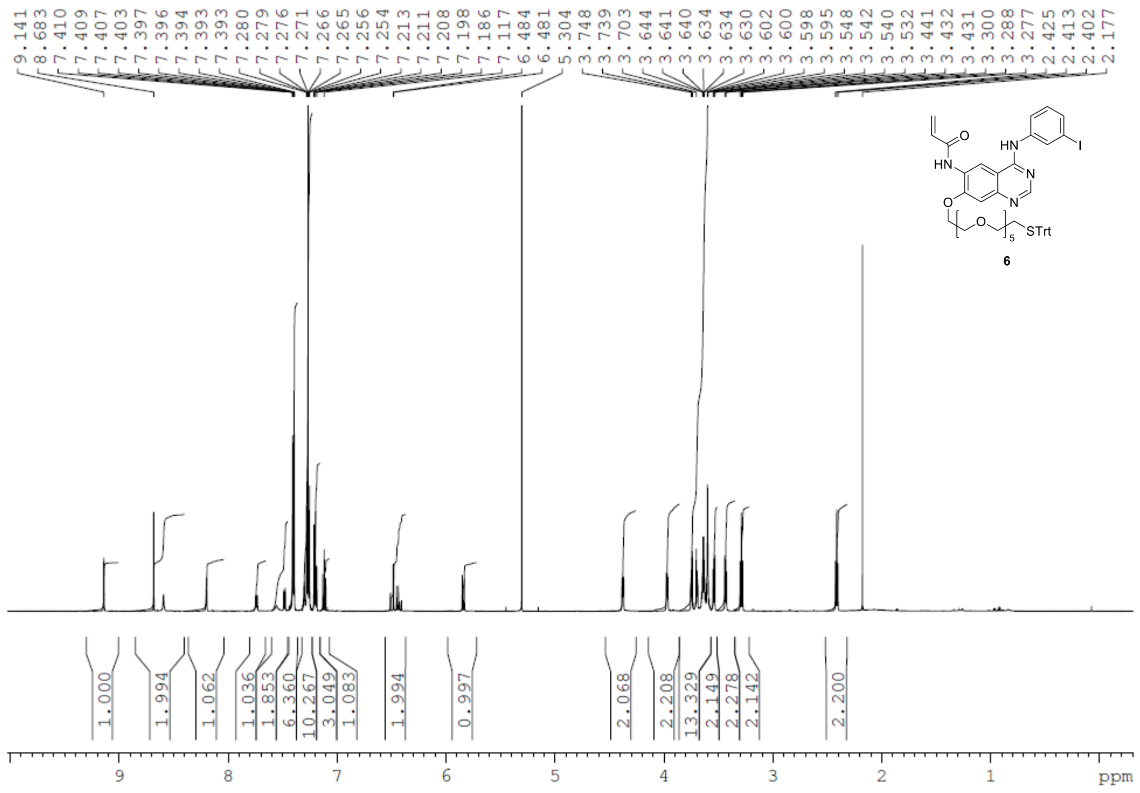
9.141
8.683
7.410
7.409
7.407
7.403
7.397
7.396
7.394
7.393
7.393
7.280
7.279
7.276
7.271
7.266
7.265
7.256
7.254
7.213
7.211
7.208
7.198
7.186
7.117
6.484
6.481
5.304
3.748
3.739
3.703
3.644
3.641
3.640
3.634
3.634
3.630
3.602
3.600
3.598
3.595
3.548
3.542
3.540
3.532
3.441
3.432
3.431
3.300
3.288
3.277
2.425
2.413
2.402
2.177
O
HN
HN
I
N
N
O
O
5
STrt
6
1.000
1.994
1.062
1.036
1.853
6.360
10.267
3.049
1.083
1.994
0.997
2.068
2.208
13.329
2.149
2.278
2.142
2.200
9
8
7
6
5
4
3
2
1
ppm

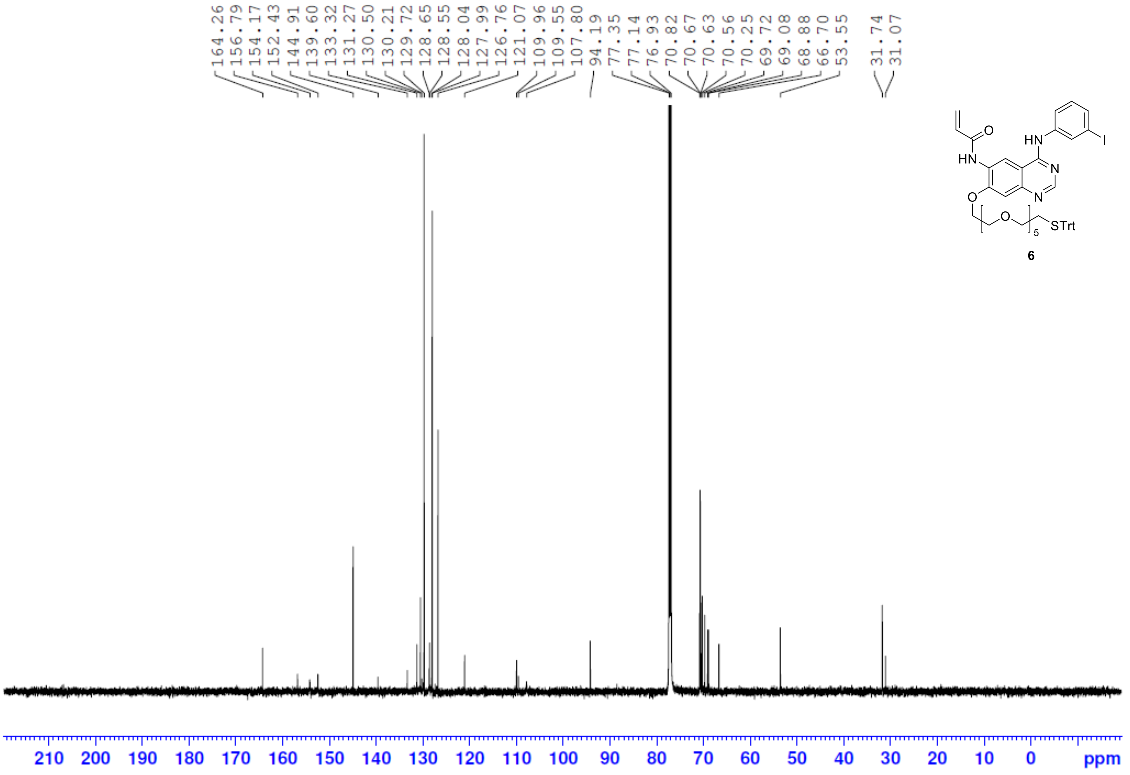
164.26
156.79
154.17
152.43
144.91
139.60
133.32
131.27
130.50
130.21
129.72
128.65
128.55
128.04
127.99
126.76
121.07
109.96
109.55
107.80
94.19
77.35
77.14
76.93
70.82
70.67
70.63
70.56
70.25
69.72
69.08
68.88
66.70
53.55
31.74
31.07
O
HN
HN
I
N
N
O
O
5
STrt
6
210
200
190
180
170
160
150
140
130
120
110
100
90
80
70
60
50
40
30
20
10
0
ppm

Compound 8

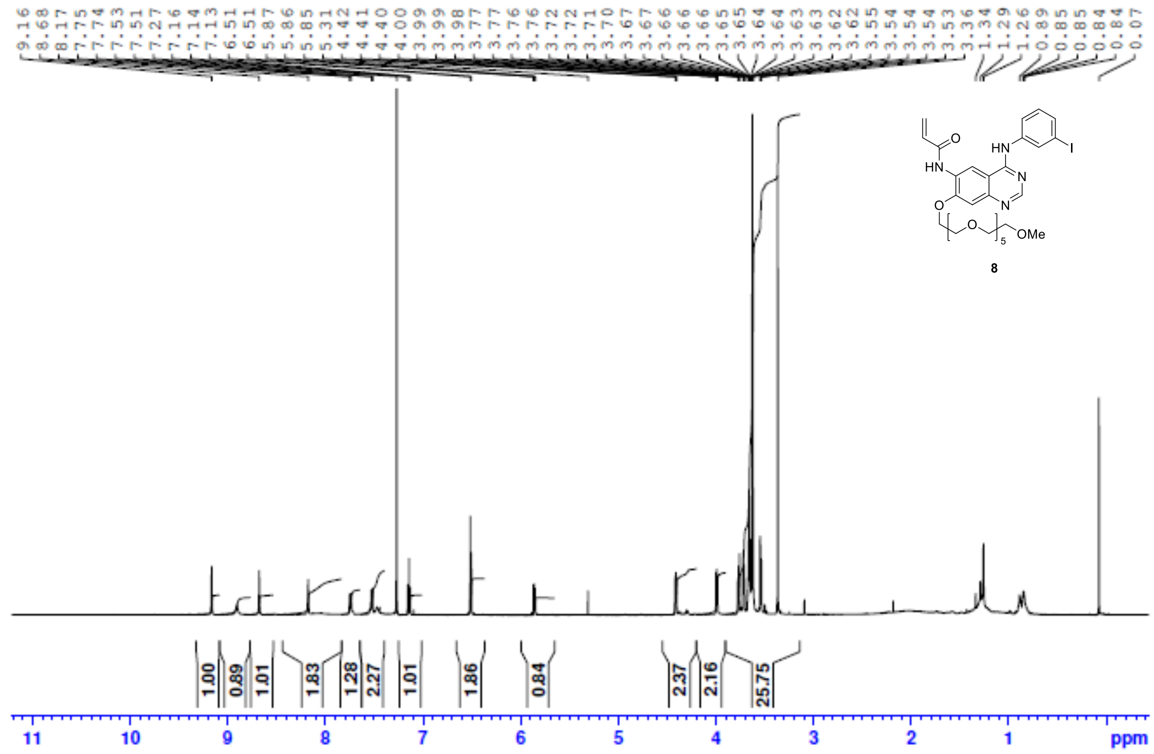
9.16
8.68
8.17
7.75
7.74
7.53
7.51
7.27
7.16
7.14
7.13
6.51
6.51
5.87
5.86
5.85
5.31
4.42
4.41
4.40
4.00
3.99
3.99
3.98
3.77
3.77
3.76
3.76
3.72
3.72
3.71
3.70
3.67
3.67
3.66
3.66
3.65
3.65
3.64
3.64
3.63
3.63
3.62
3.62
3.55
3.54
3.54
3.54
3.53
3.36
1.34
1.29
1.26
0.89
0.85
0.85
0.84
0.84
0.07
HN
O
HN
I
N
N
O
O
5
OMe
8
1.00
0.89
1.01
1.83
1.28
2.27
1.01
1.86
0.84
2.37
2.16
25.75
11
10
9
8
7
6
5
4
3
2
1
ppm

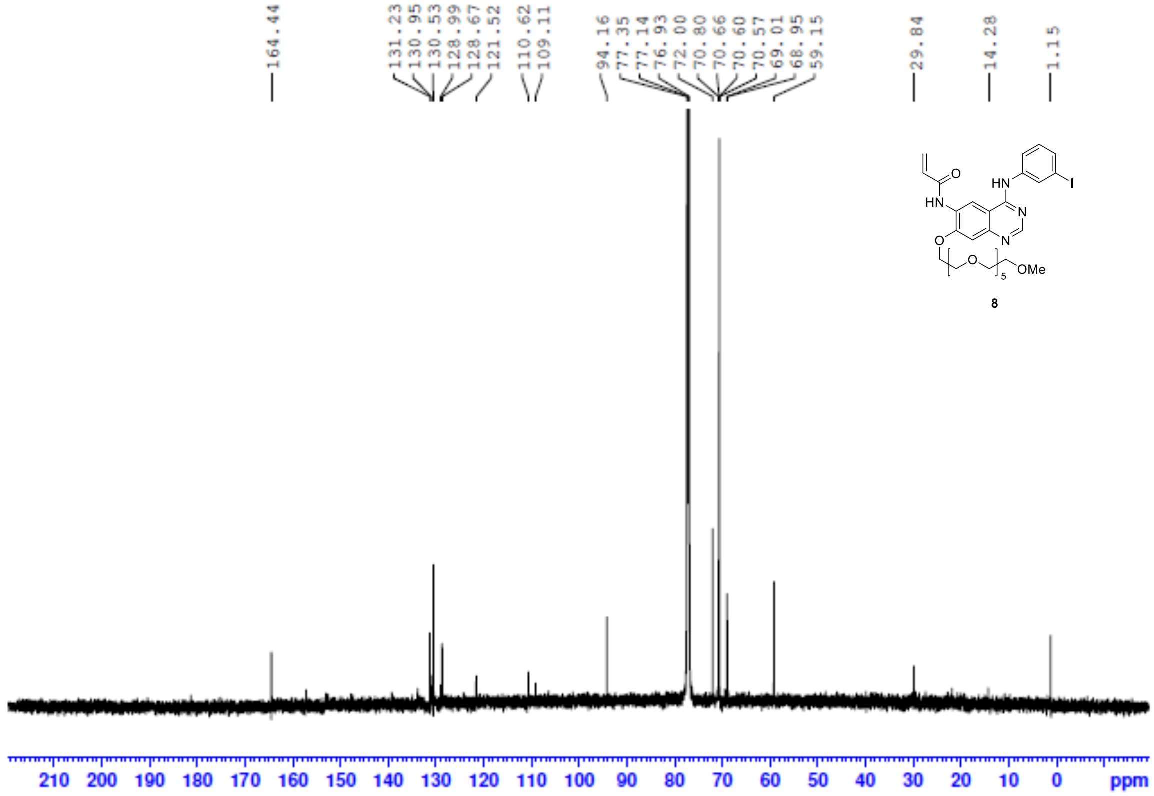
164.44
131.23
130.95
130.53
128.99
128.67
121.52
110.62
109.11
94.16
77.35
77.14
76.93
72.00
70.80
70.66
70.60
70.57
69.01
68.95
59.15
29.84
14.28
1.15
HN
O
HN
I
N
N
O
O
5
OMe
8
210
200
190
180
170
160
150
140
130
120
110
100
90
80
70
60
50
40
30
20
10
0
ppm

Compound 10

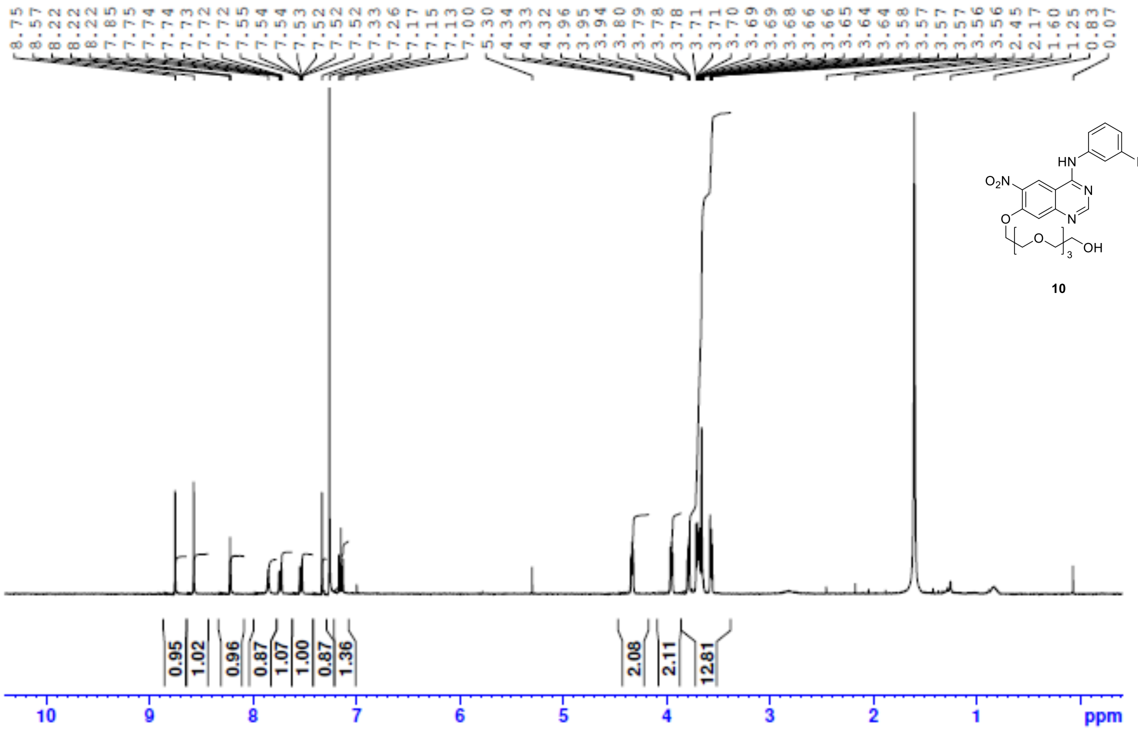

Compound 11

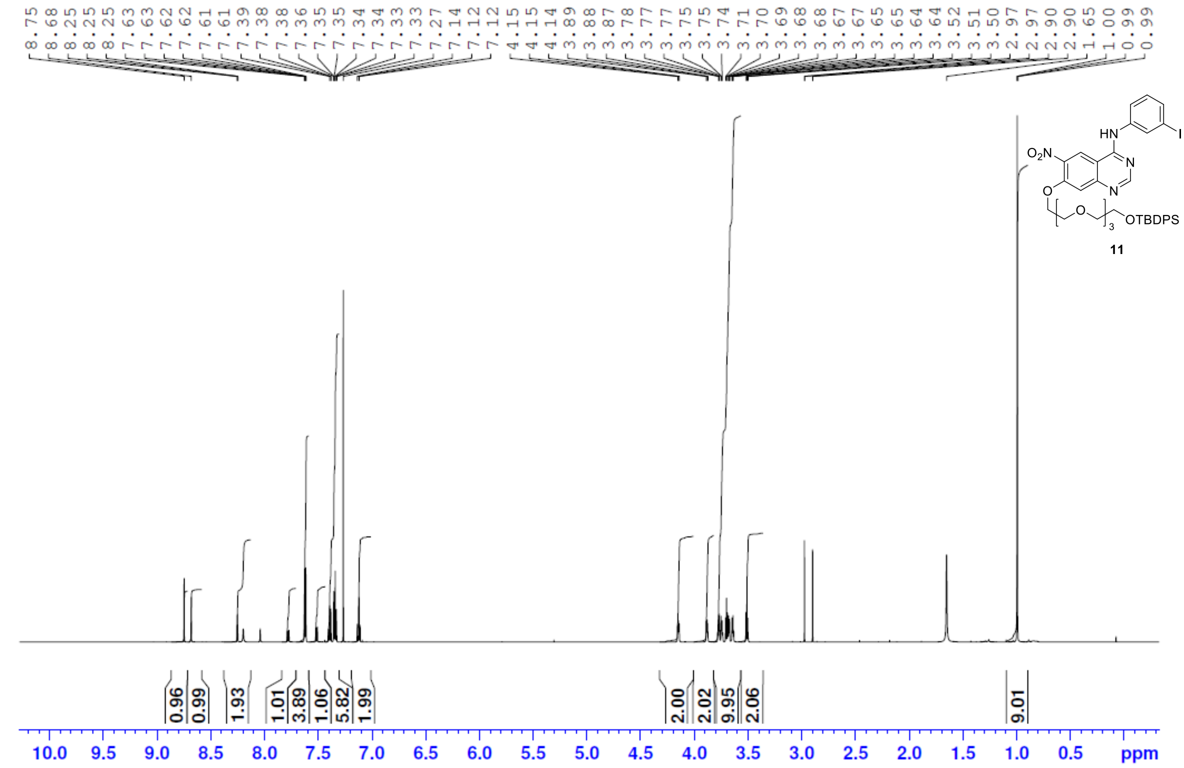
8.75 8.68 8.25 8.25 8.25 7.63 7.63 7.62 7.62 7.61 7.61 7.39 7.38 7.38 7.36 7.35 7.35 7.34 7.34 7.33 7.33 7.27 7.14 7.12 4.15 4.15 4.14 3.89 3.88 3.87 3.78 3.77 3.77 3.75 3.75 3.74 3.71 3.70 3.69 3.68 3.68 3.67 3.67 3.65 3.65 3.64 3.64 3.52 3.51 3.50 2.97 2.90 1.65 1.00 0.99 0.99
HN
I
O_2N
N
O
O
3
OTBDPS
11
0.96 0.99 1.93 1.01 3.89 1.06 5.82 1.99 2.00 2.02 9.95 2.06 9.01
10.0 9.5 9.0 8.5 8.0 7.5 7.0 6.5 6.0 5.5 5.0 4.5 4.0 3.5 3.0 2.5 2.0 1.5 1.0 0.5 ppm

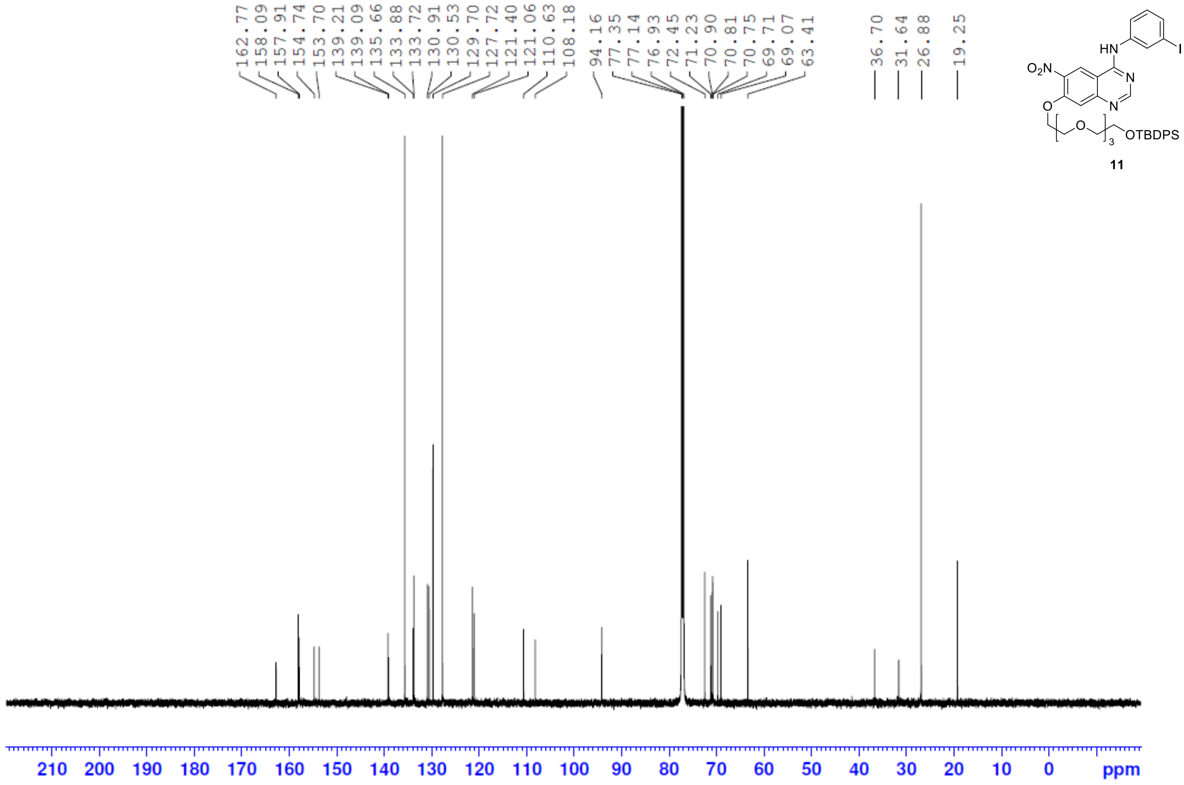
162.77 158.09 157.91 154.74 153.70 139.21 139.09 135.66 133.88 133.72 130.91 130.53 129.70 127.72 121.40 121.06 110.63 108.18 94.16 77.35 77.14 76.93 72.45 71.23 70.90 70.81 70.75 69.71 69.07 63.41 36.70 31.64 26.88 19.25
HN
I
O_2N
N
O
O
3
OTBDPS
11
210 200 190 180 170 160 150 140 130 120 110 100 90 80 70 60 50 40 30 20 10 0 ppm

Compound 12

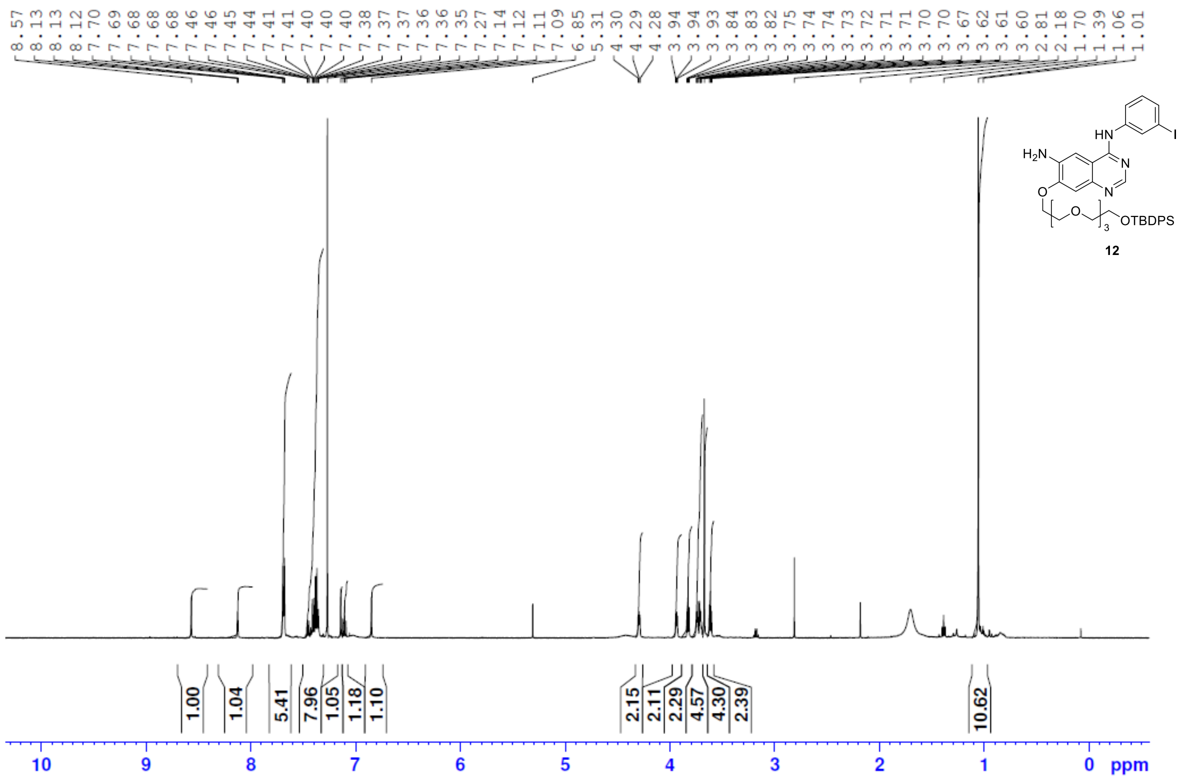

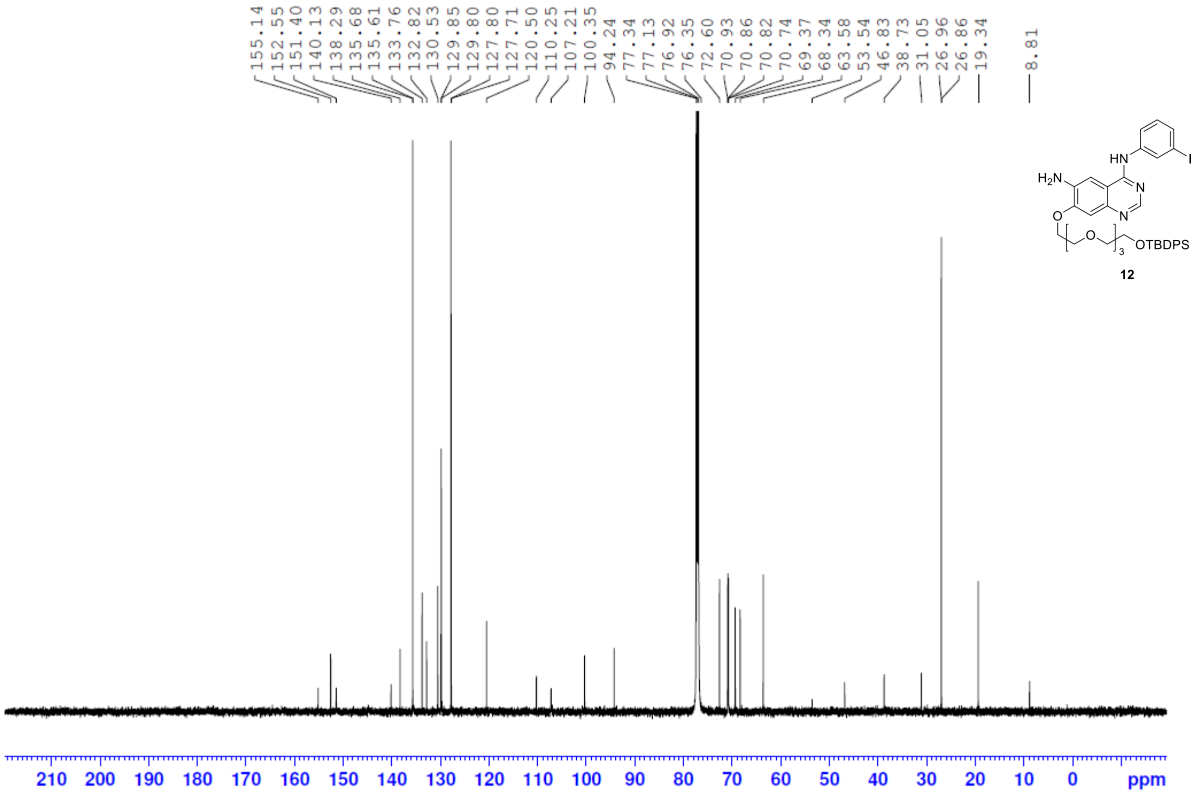

Compound 13

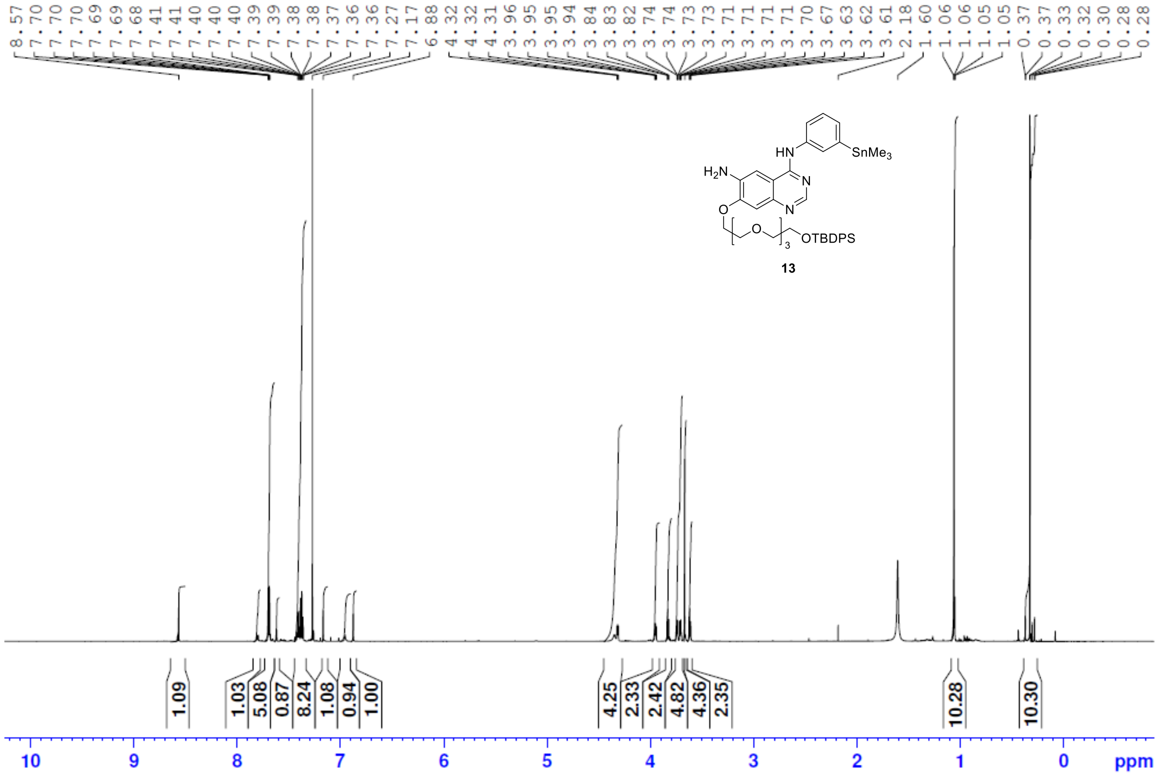
8.57 7.70 7.70 7.70 7.69 7.69 7.68 7.41 7.41 7.40 7.40 7.39 7.39 7.38 7.38 7.37 7.36 7.36 7.27 7.17 6.88 4.32 4.32 4.31 3.96 3.95 3.95 3.94 3.84 3.83 3.82 3.74 3.74 3.73 3.73 3.71 3.71 3.71 3.70 3.67 3.63 3.62 3.61 2.18 1.60 1.06 1.06 1.05 1.05 0.37 0.33 0.32 0.30 0.28 0.28
HN
SnMe3
H2N
N
N
O
O
3
OTBDPS
13
1.09 1.03 5.08 0.87 8.24 1.08 0.94 1.00 4.25 2.33 2.42 4.82 4.36 2.35 10.28 10.30
10 9 8 7 6 5 4 3 2 1 0 ppm

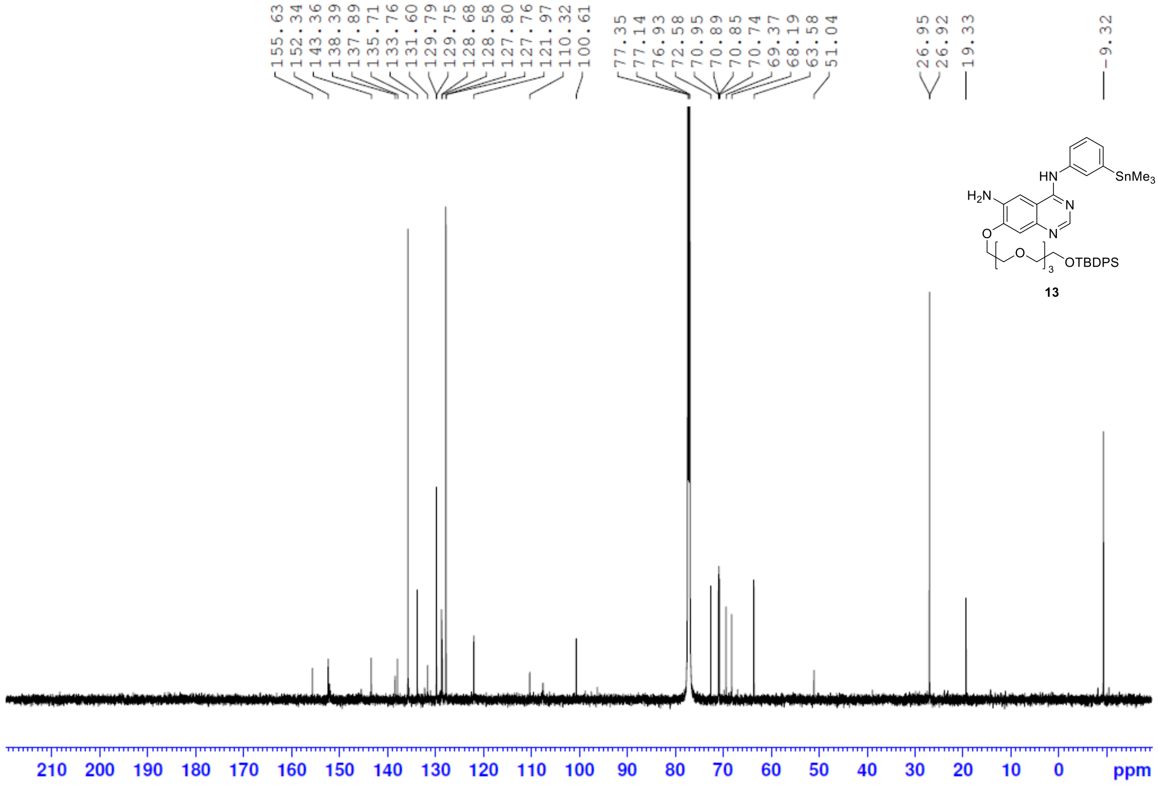
155.63 152.34 143.36 138.39 137.89 135.71 133.76 131.60 129.79 129.75 128.68 128.58 127.80 127.76 121.97 110.32 100.61 77.35 77.14 76.93 72.58 70.95 70.89 70.85 70.74 69.37 68.19 63.58 51.04 26.95 26.92 19.33 -9.32
HN
SnMe3
H2N
N
N
O
O
3
OTBDPS
13
210 200 190 180 170 160 150 140 130 120 110 100 90 80 70 60 50 40 30 20 10 0 ppm

Compound 14

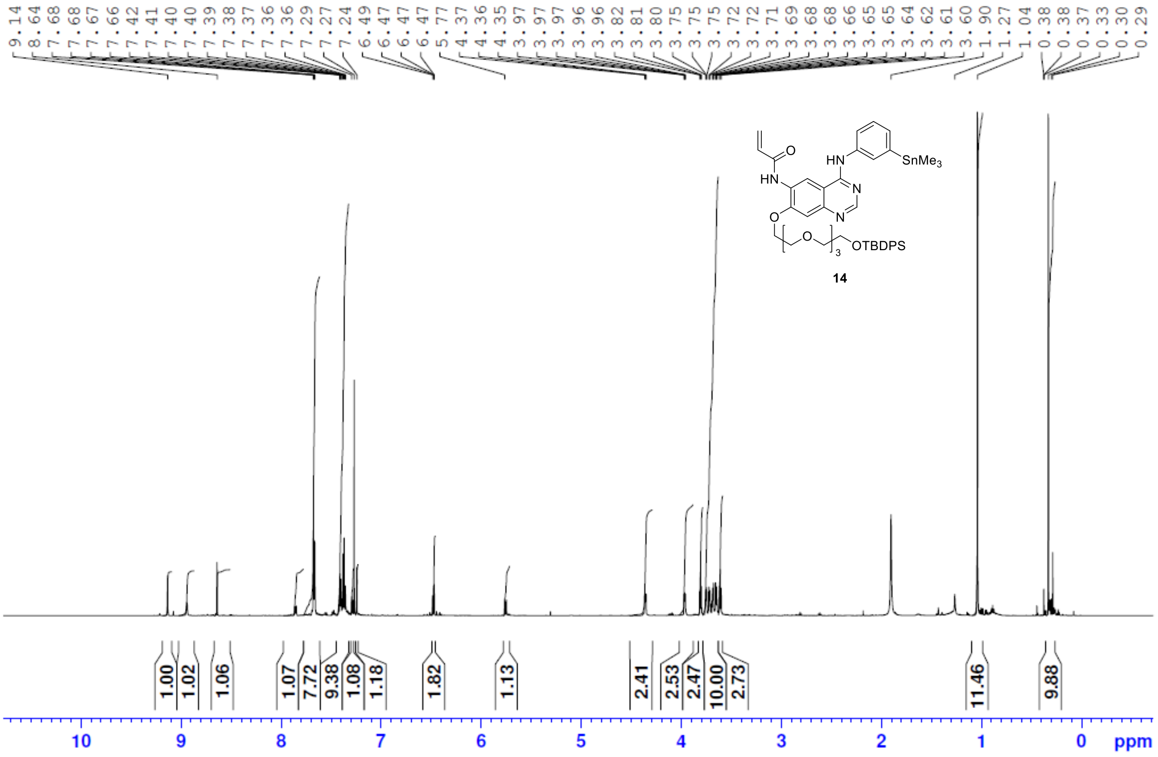
9.14 8.64 7.68 7.68 7.67 7.66 7.42 7.41 7.40 7.40 7.39 7.38 7.37 7.36 7.29 7.27 7.24 6.49 6.47 6.47 6.47 5.77 4.37 4.36 4.35 3.97 3.97 3.96 3.96 3.82 3.81 3.80 3.75 3.75 3.75 3.72 3.72 3.71 3.69 3.68 3.68 3.66 3.65 3.65 3.64 3.62 3.61 3.60 1.90 1.27 1.04 0.38 0.38 0.37 0.33 0.30 0.29
HN
O
HN
SnMe$_3$
N
N
O
O
3
OTBDPS
14
1.00 1.02 1.06 1.07 7.72 9.38 1.08 1.18 1.82 1.13 2.41 2.53 2.47 10.00 2.73 11.46 9.88
10 9 8 7 6 5 4 3 2 1 0 ppm

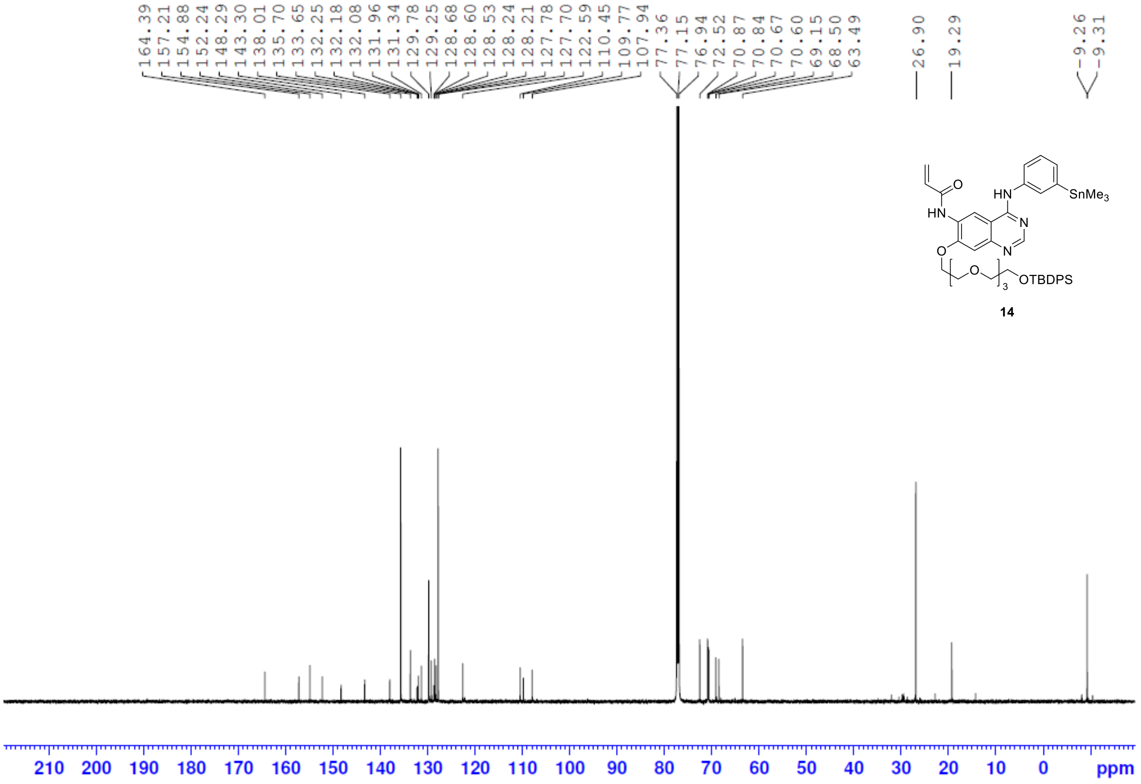
164.39 157.21 154.88 152.24 148.29 143.30 138.01 135.70 133.65 132.25 132.18 132.08 131.96 131.34 129.78 129.25 128.68 128.60 128.53 128.24 128.21 127.78 127.70 122.59 110.45 109.77 107.94 77.36 77.15 76.94 72.52 70.87 70.84 70.67 70.60 69.15 68.50 63.49
26.90
19.29
-9.26 -9.31
HN
O
HN
SnMe$_3$
N
N
O
O
3
OTBDPS
14
210 200 190 180 170 160 150 140 130 120 110 100 90 80 70 60 50 40 30 20 10 0 ppm

Compound 15

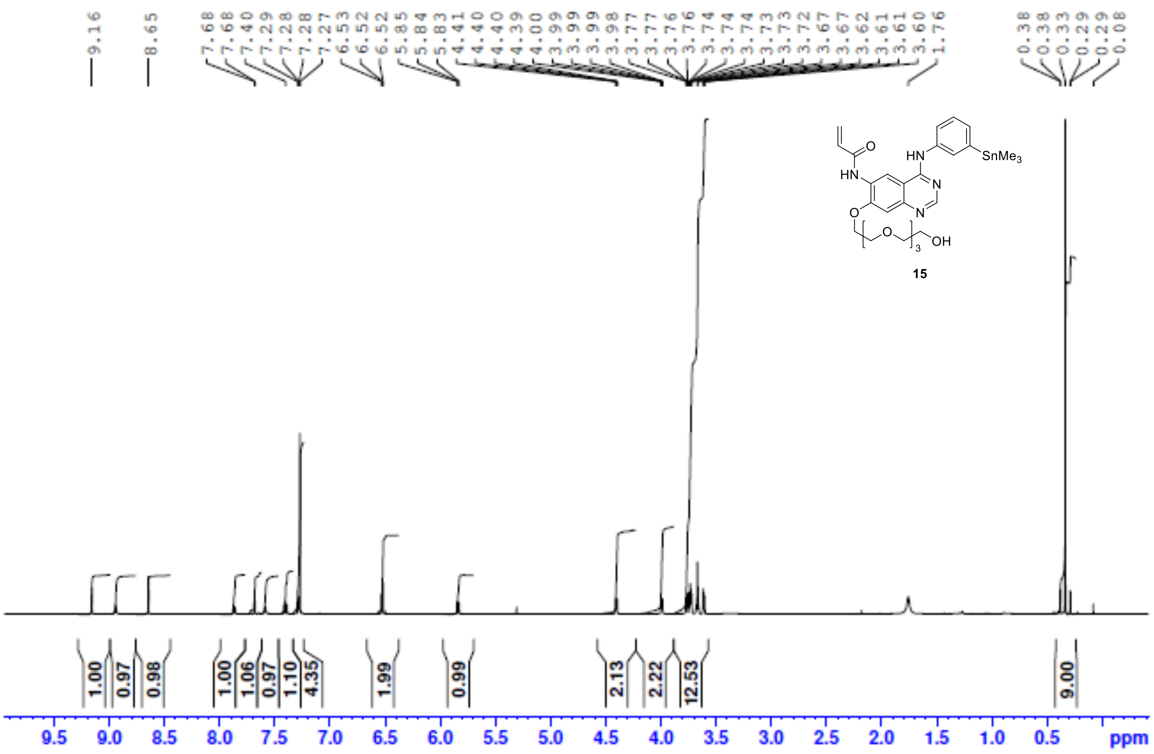
9.16
8.65
7.68 7.68 7.40 7.29 7.28 7.28 7.27 6.53 6.52 6.52 5.85 5.84 5.83 4.41 4.40 4.40 4.39 4.00 3.99 3.99 3.99 3.98 3.77 3.77 3.76 3.76 3.74 3.74 3.73 3.73 3.73 3.72 3.67 3.67 3.62 3.61 3.61 3.60 1.76
0.38 0.38 0.33 0.29 0.29 0.08
HN
O
SnMe3
N
OH
3
15
1.00 0.97 0.98 1.00 1.06 0.97 1.10 4.35 1.99 0.99 2.13 2.22 12.53 9.00
9.5 9.0 8.5 8.0 7.5 7.0 6.5 6.0 5.5 5.0 4.5 4.0 3.5 3.0 2.5 2.0 1.5 1.0 0.5 ppm

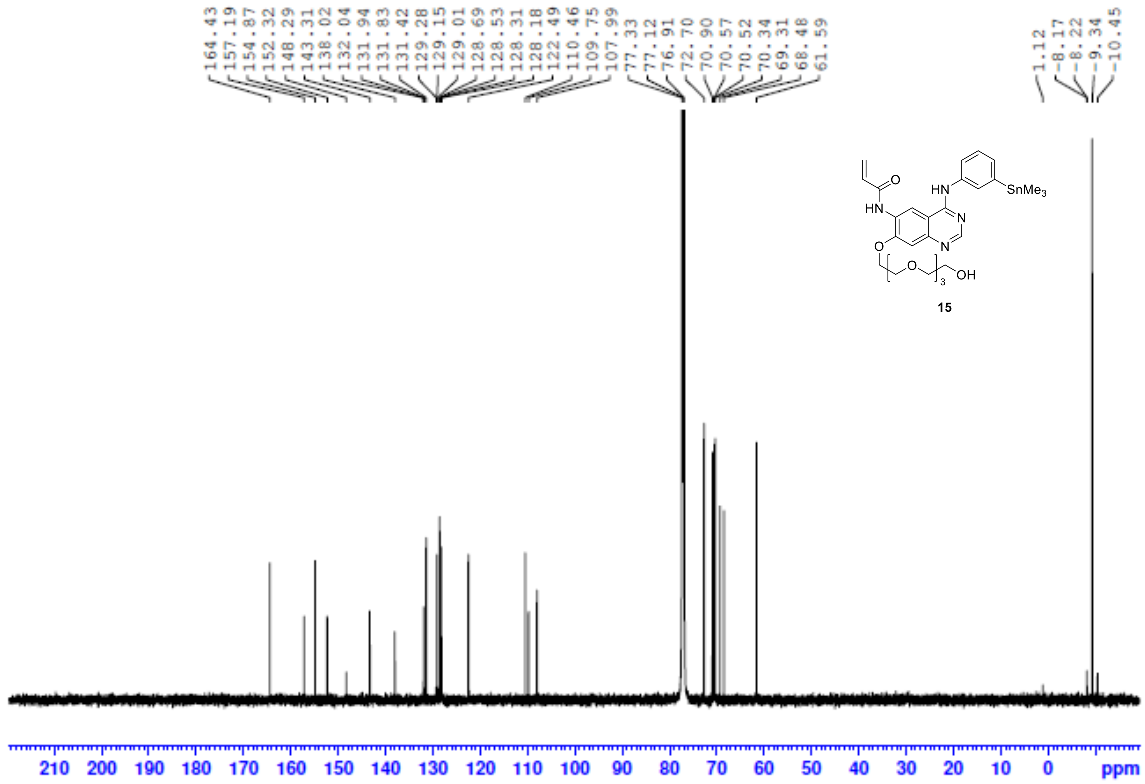
164.43 157.19 154.87 152.32 148.29 143.31 138.02 132.04 131.94 131.83 131.42 129.28 129.15 129.01 128.69 128.53 128.31 128.18 122.49 110.46 109.75 107.99
77.33 77.12 76.91 72.70 70.90 70.57 70.52 70.34 69.31 68.48 61.59
1.12 -8.17 -8.22 -9.34 -10.45
HN
O
SnMe3
N
OH
3
15
210 200 190 180 170 160 150 140 130 120 110 100 90 80 70 60 50 40 30 20 10 0 ppm

Compound 16

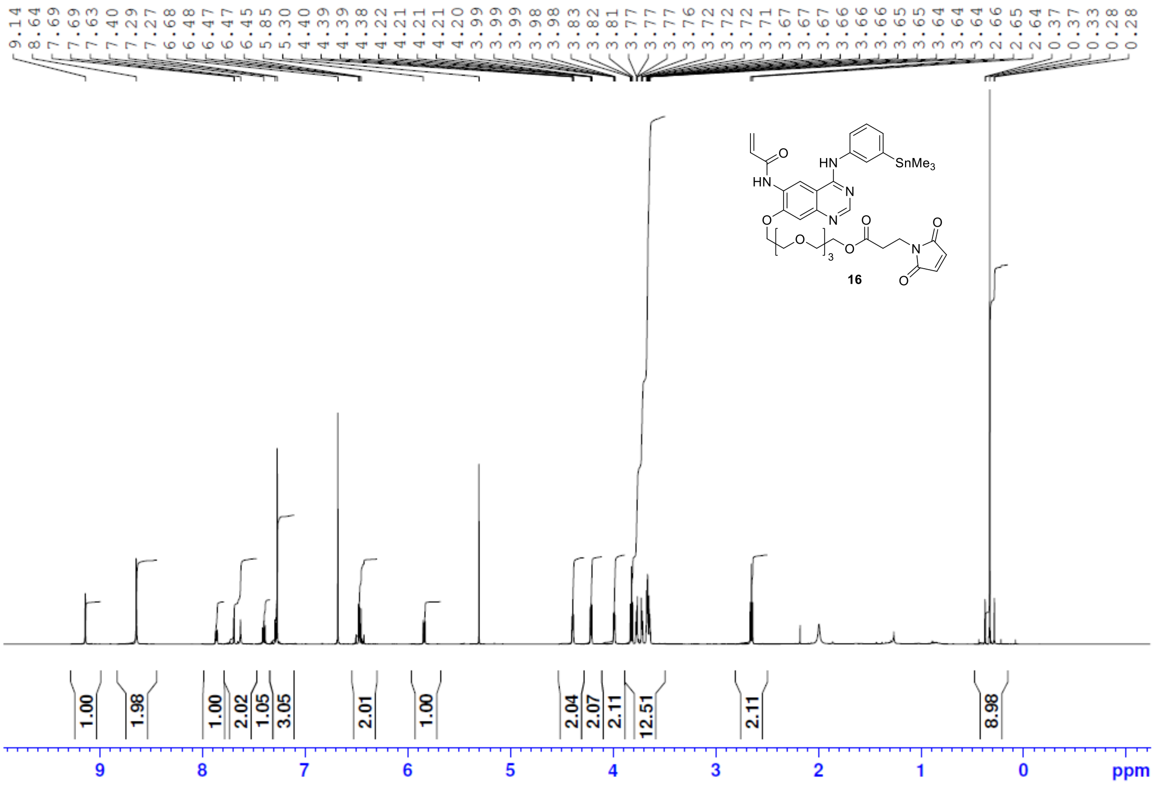
9.14
8.64
7.69
7.63
7.40
7.29
7.27
6.68
6.48
6.47
6.47
6.45
5.85
5.30
4.40
4.39
4.39
4.38
4.22
4.21
4.21
4.21
4.20
3.99
3.99
3.99
3.98
3.98
3.83
3.82
3.81
3.77
3.77
3.77
3.76
3.72
3.72
3.72
3.71
3.67
3.67
3.67
3.66
3.66
3.66
3.65
3.65
3.64
3.64
3.64
2.66
2.65
2.64
0.37
0.37
0.33
0.28
0.28
16
1.00
1.98
1.00
2.02
1.05
3.05
2.01
1.00
2.04
2.07
2.11
12.51
2.11
8.98
9
8
7
6
5
4
3
2
1
0
ppm

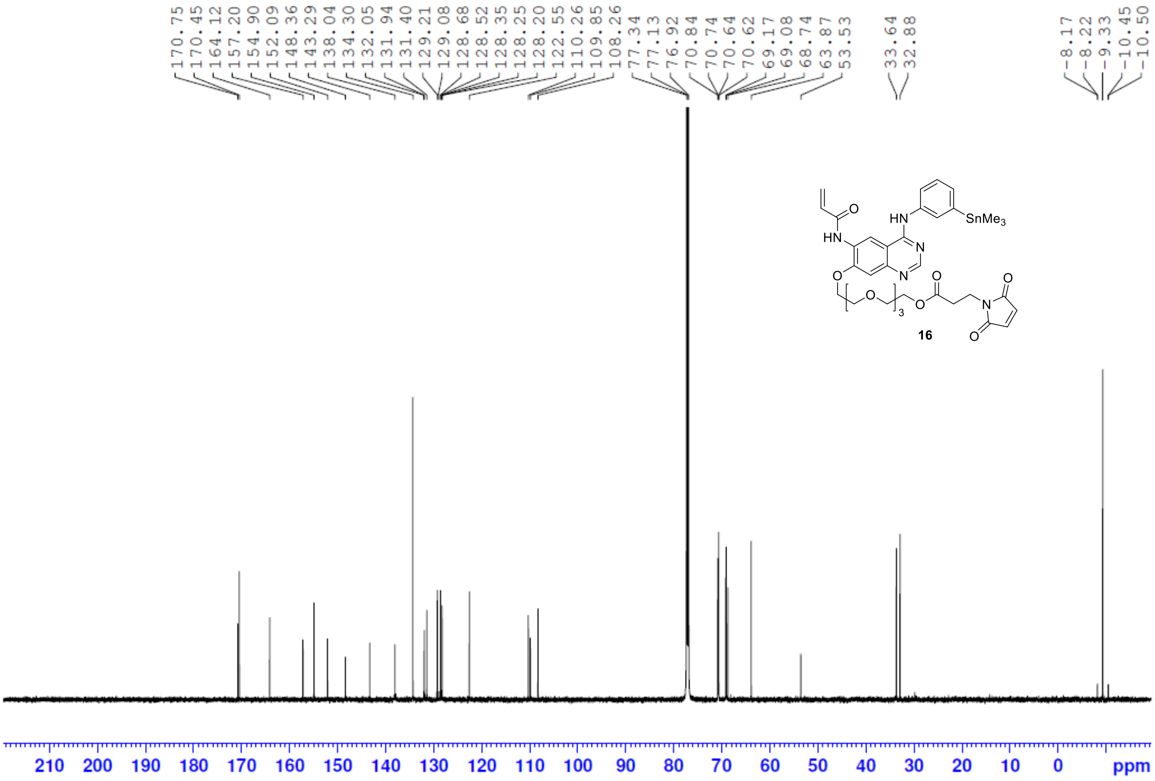
170.75
170.45
164.12
157.20
154.90
152.09
148.36
143.29
138.04
134.30
132.05
131.94
131.40
129.21
129.08
128.68
128.52
128.35
128.25
128.20
122.55
110.26
109.85
108.26
77.34
77.13
76.92
70.84
70.74
70.64
70.62
69.17
69.08
68.74
63.87
53.53
33.64
32.88
-8.17
-8.22
-9.33
-10.45
-10.50
16
210
200
190
180
170
160
150
140
130
120
110
100
90
80
70
60
50
40
30
20
10
0
ppm

Compound S1

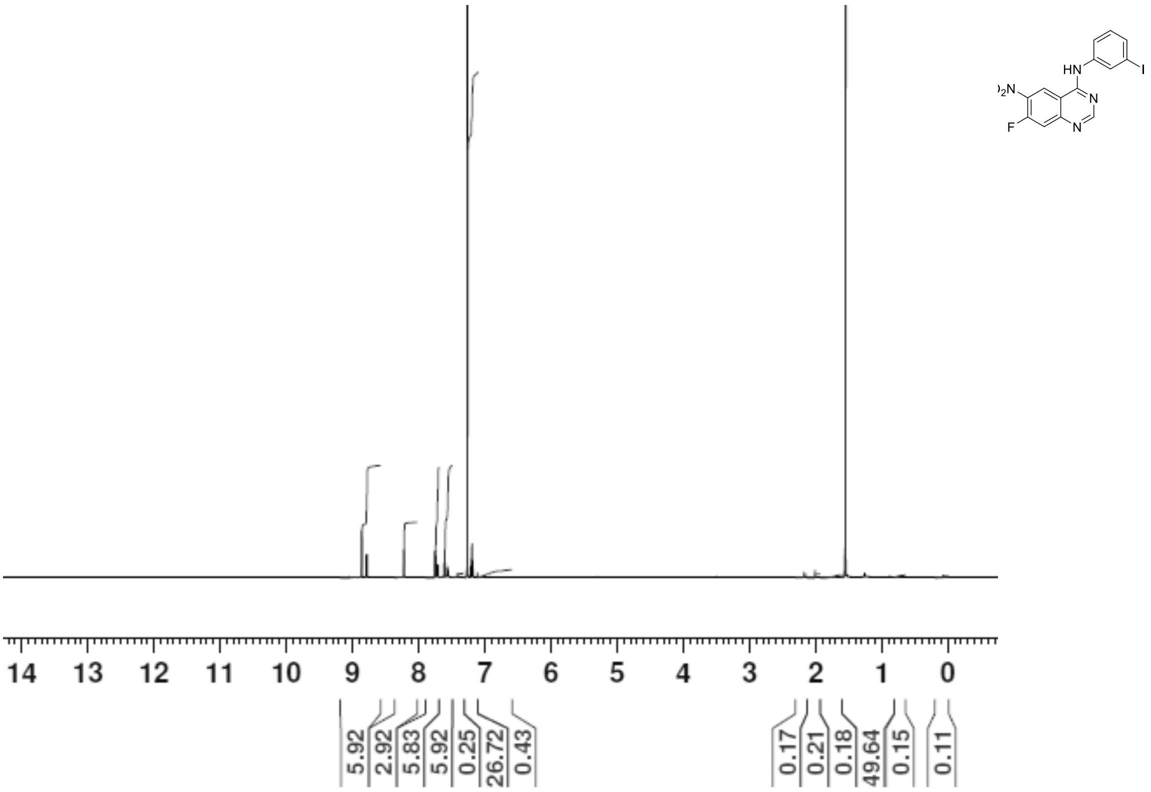

Compound S2

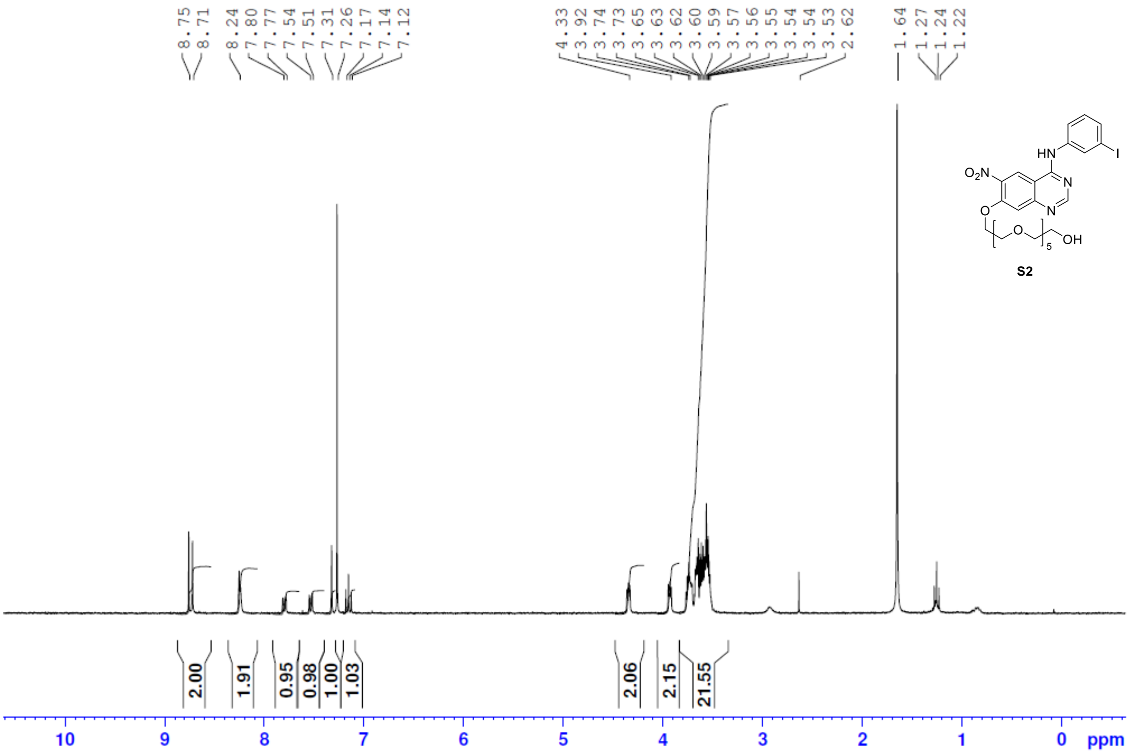

Compound S3

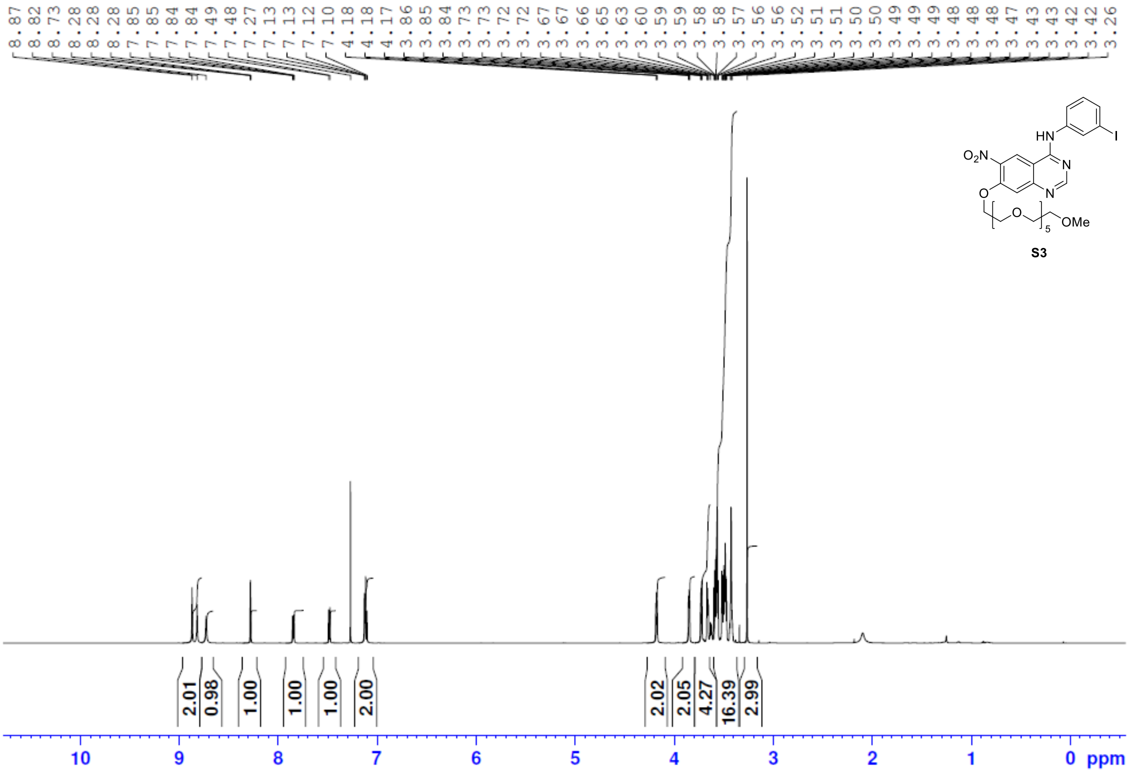
8.87 8.82 8.73 8.28 8.28 8.28 7.85 7.85 7.84 7.84 7.49 7.48 7.27 7.13 7.13 7.12 7.10 4.18 4.17 3.86 3.85 3.84 3.73 3.73 3.72 3.72 3.67 3.67 3.66 3.65 3.63 3.60 3.59 3.59 3.58 3.58 3.57 3.56 3.52 3.51 3.51 3.50 3.50 3.49 3.49 3.48 3.48 3.48 3.47 3.43 3.43 3.42 3.42 3.26
2.01
0.98
1.00
1.00
1.00
2.00
2.02
2.05
4.27
16.39
2.99
10 9 8 7 6 5 4 3 2 1 0 ppm
HN
I
O_2N
N
N
O
O
5
OMe
S3

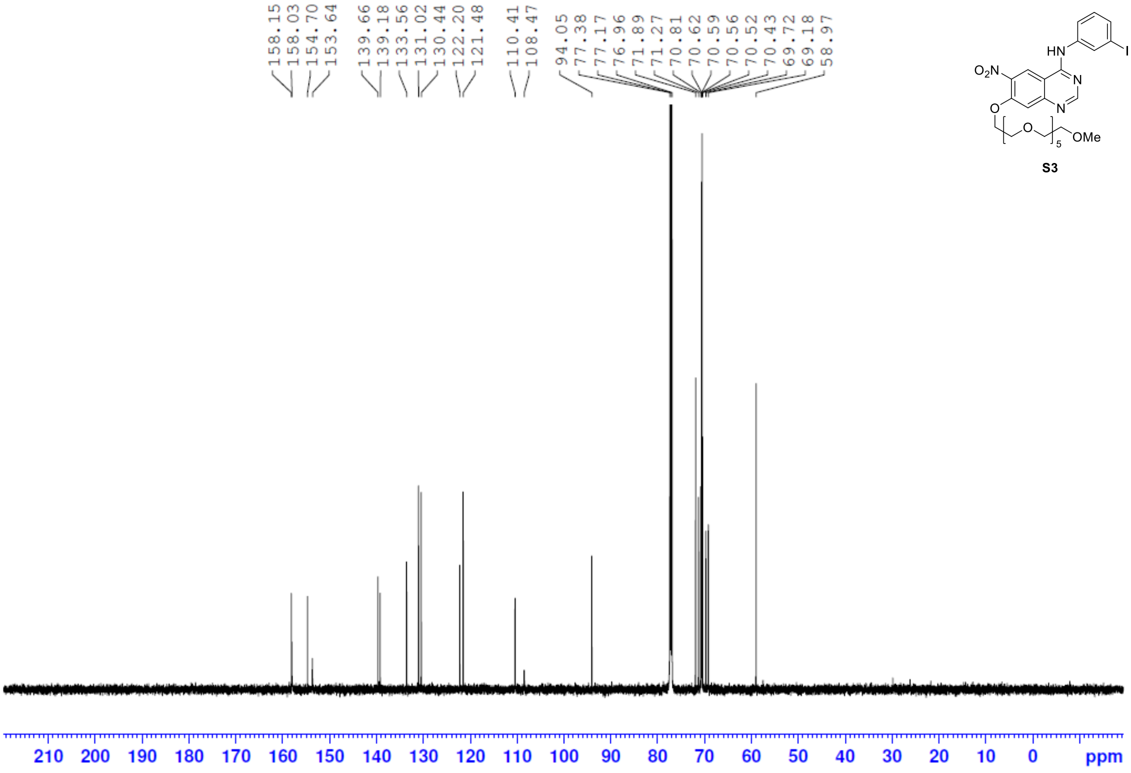
158.15 158.03 154.70 153.64 139.66 139.18 133.56 131.02 130.44 122.20 121.48 110.41 108.47 94.05 77.38 77.17 76.96 71.89 71.27 70.81 70.62 70.59 70.56 70.52 70.43 69.72 69.18 58.97
210 200 190 180 170 160 150 140 130 120 110 100 90 80 70 60 50 40 30 20 10 0 ppm
HN
I
O_2N
N
N
O
O
5
OMe
S3

Compound S4

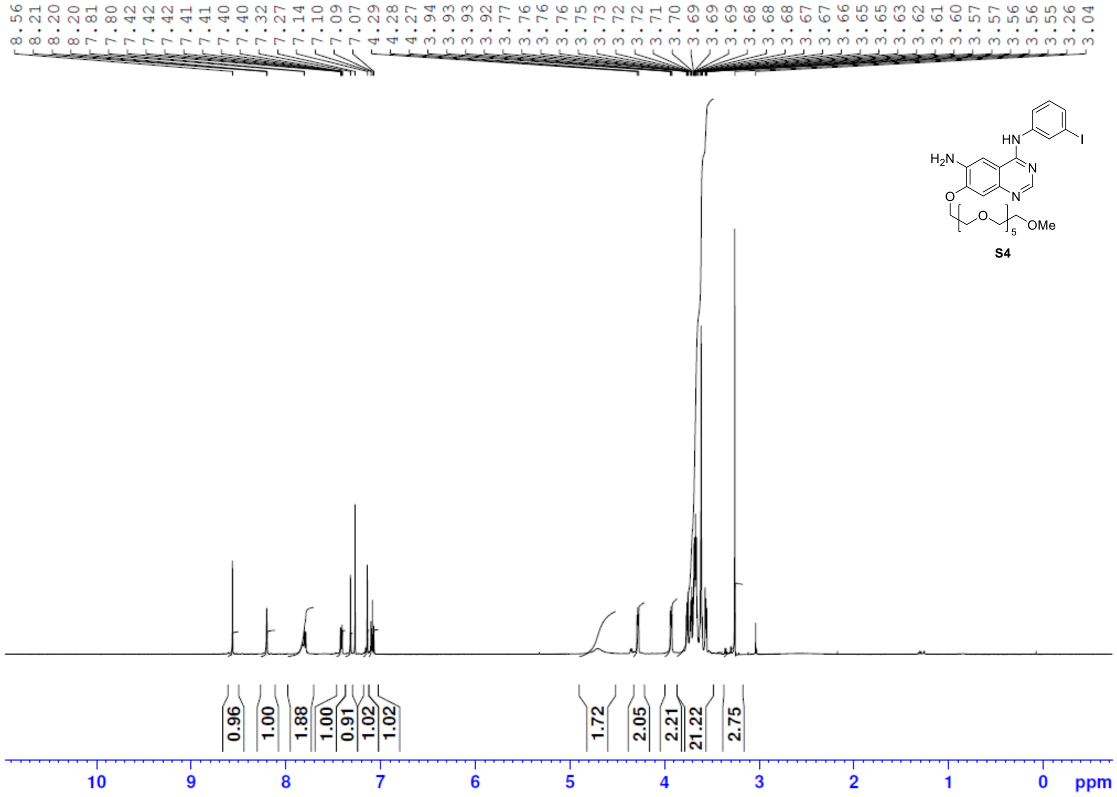
8.56
8.21
8.20
7.81
7.80
7.42
7.42
7.42
7.41
7.41
7.40
7.40
7.32
7.27
7.14
7.10
7.09
7.07
4.29
4.28
4.27
3.94
3.93
3.93
3.92
3.77
3.76
3.76
3.75
3.73
3.72
3.71
3.70
3.69
3.69
3.69
3.68
3.68
3.67
3.67
3.66
3.65
3.65
3.63
3.62
3.61
3.60
3.57
3.57
3.56
3.56
3.55
3.26
3.04
HN
I
H2N
N
N
O
O
5
OMe
S4
0.96
1.00
1.88
1.00
0.91
1.02
1.02
1.72
2.05
2.21
21.22
2.75
10
9
8
7
6
5
4
3
2
1
0
ppm

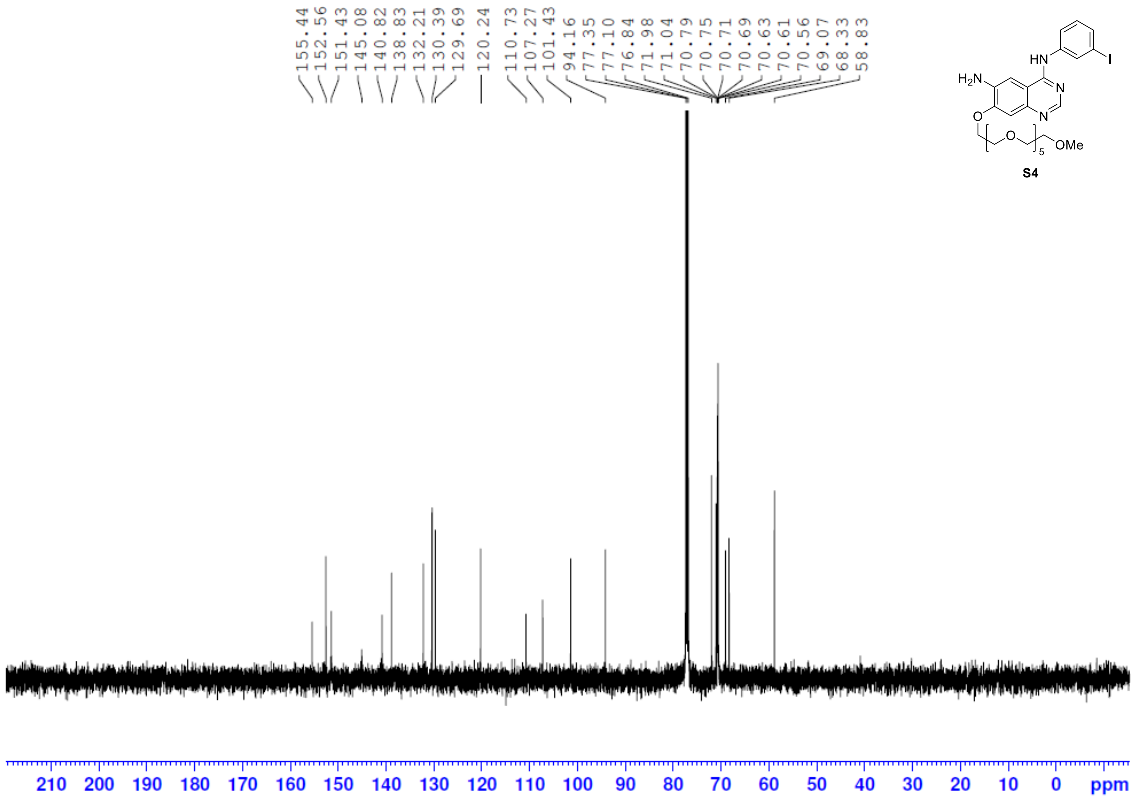
155.44
152.56
151.43
145.08
140.82
138.83
132.21
130.39
129.69
120.24
110.73
107.27
101.43
94.16
77.35
77.10
76.84
71.98
71.04
70.79
70.75
70.71
70.69
70.63
70.61
70.56
69.07
68.33
58.83
HN
I
H2N
N
N
O
O
5
OMe
S4
210
200
190
180
170
160
150
140
130
120
110
100
90
80
70
60
50
40
30
20
10
0
ppm

Compound S5

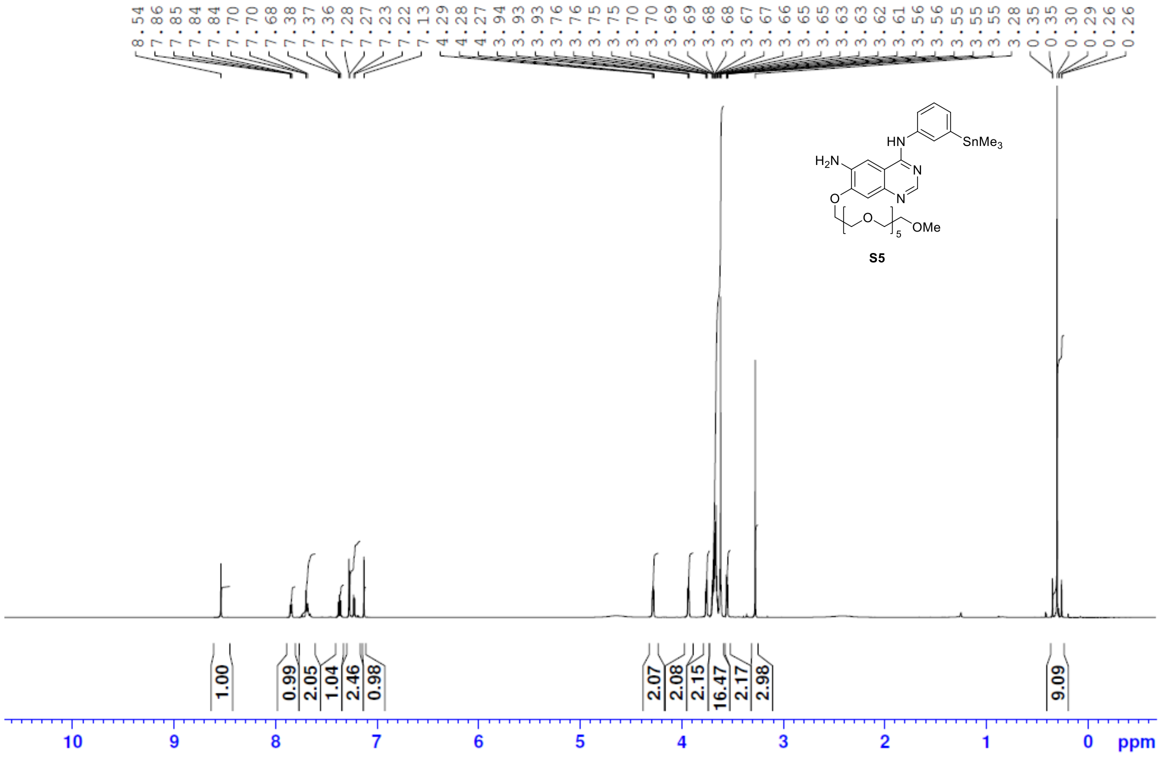

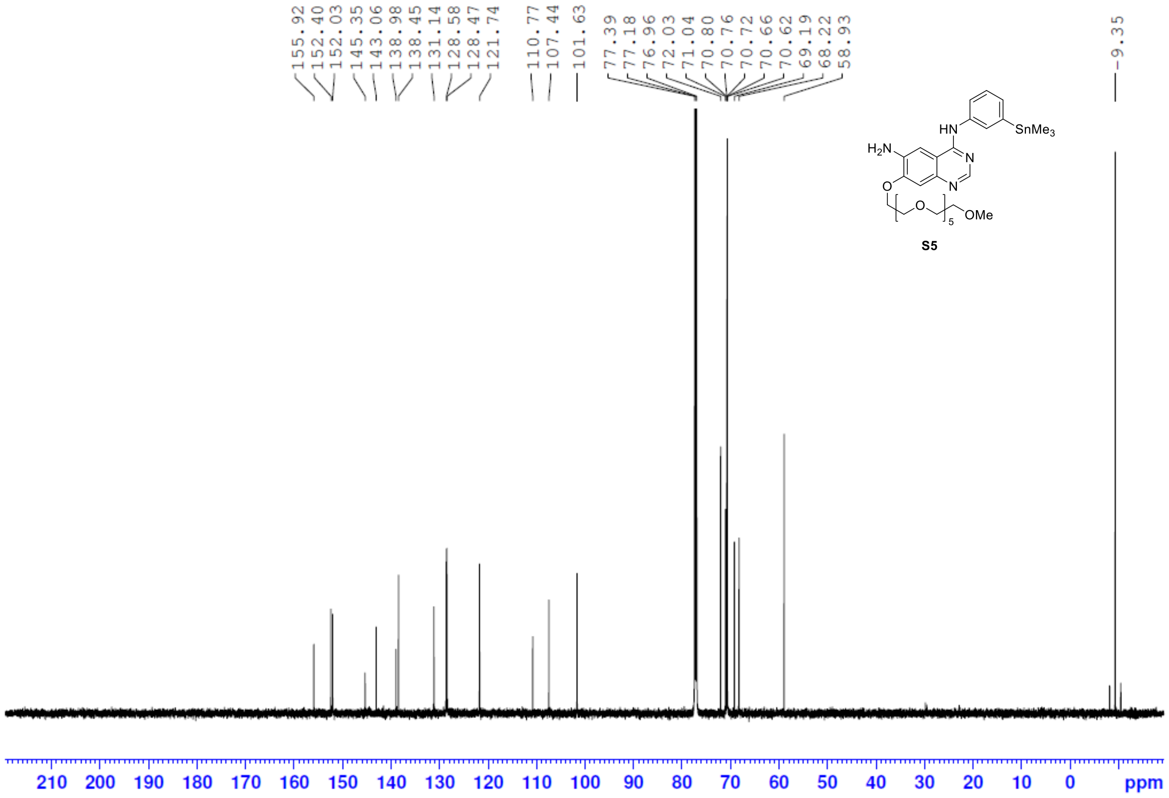

Compound S6

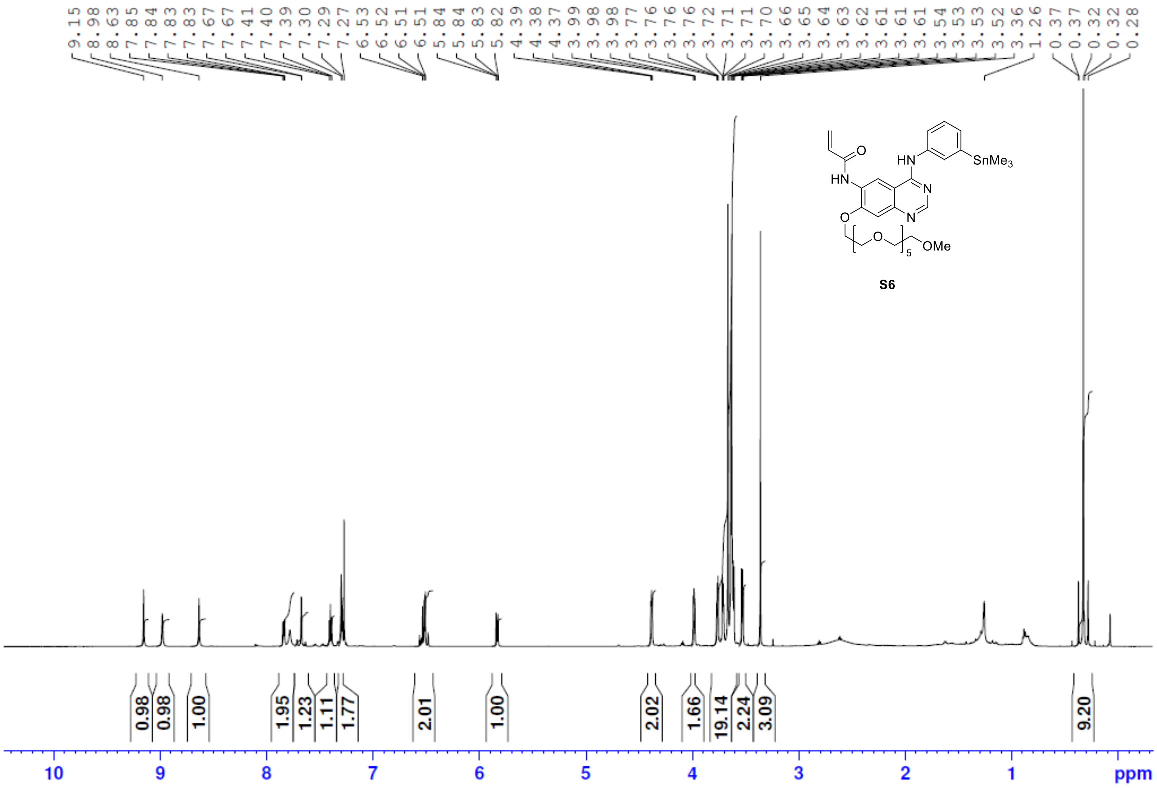
9.15 8.98 8.63 7.85 7.84 7.83 7.83 7.67 7.67 7.41 7.40 7.39 7.30 7.29 7.27 6.53 6.52 6.51 6.51 5.84 5.84 5.83 5.82 4.39 4.38 4.37 3.99 3.98 3.98 3.77 3.76 3.76 3.76 3.72 3.71 3.71 3.70 3.66 3.65 3.64 3.63 3.62 3.61 3.61 3.61 3.54 3.53 3.53 3.52 3.36 1.26 0.37 0.37 0.32 0.32 0.28
HN
SnMe3
O
HN
N
N
O
O
5
OMe
S6
0.98 0.98 1.00 1.95 1.23 1.11 1.77 2.01 1.00 2.02 1.66 19.14 2.24 3.09 9.20
10 9 8 7 6 5 4 3 2 1 ppm

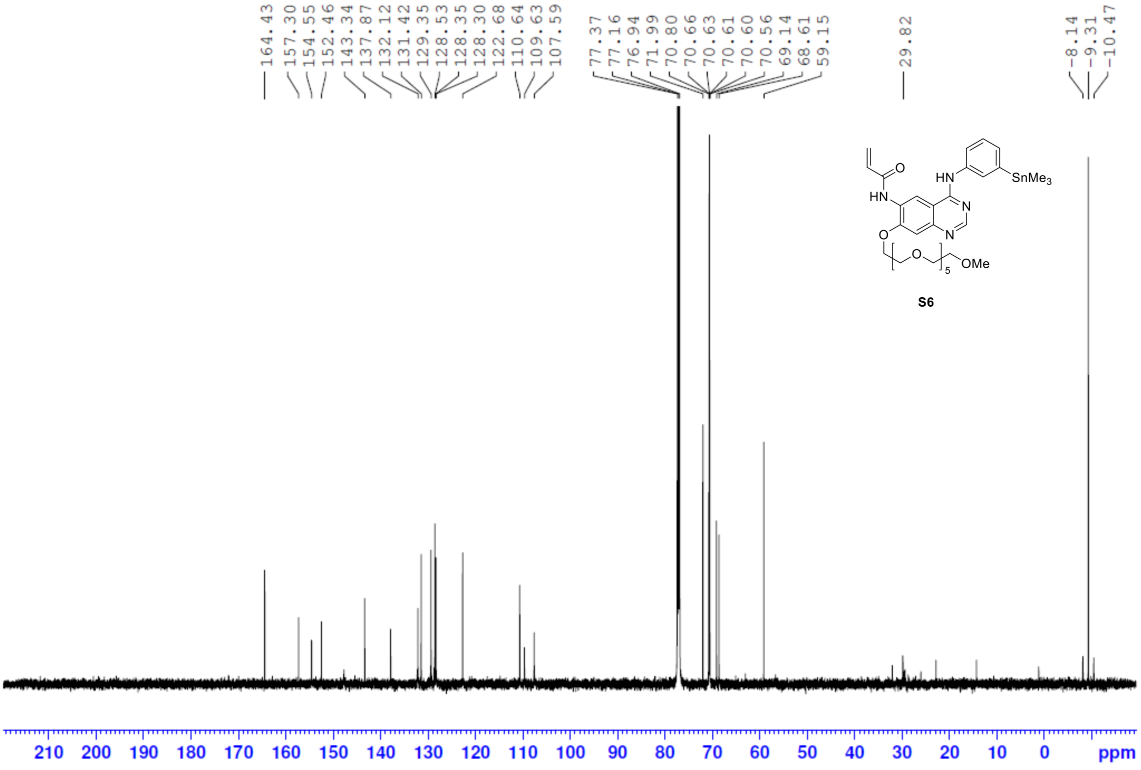
164.43 157.30 154.55 152.46 143.34 137.87 132.12 131.42 129.35 128.53 128.35 128.30 122.68 110.64 109.63 107.59 77.37 77.16 76.94 71.99 70.80 70.66 70.63 70.61 70.60 70.56 69.14 68.61 59.15 29.82 -8.14 -9.31 -10.47
HN
SnMe3
O
HN
N
N
O
O
5
OMe
S6
210 200 190 180 170 160 150 140 130 120 110 100 90 80 70 60 50 40 30 20 10 0 ppm

HPLC and HRMS for P5, $CysLys_{16}$ 7, 1, 17

AEYLR-RVRR-K16 (P5)

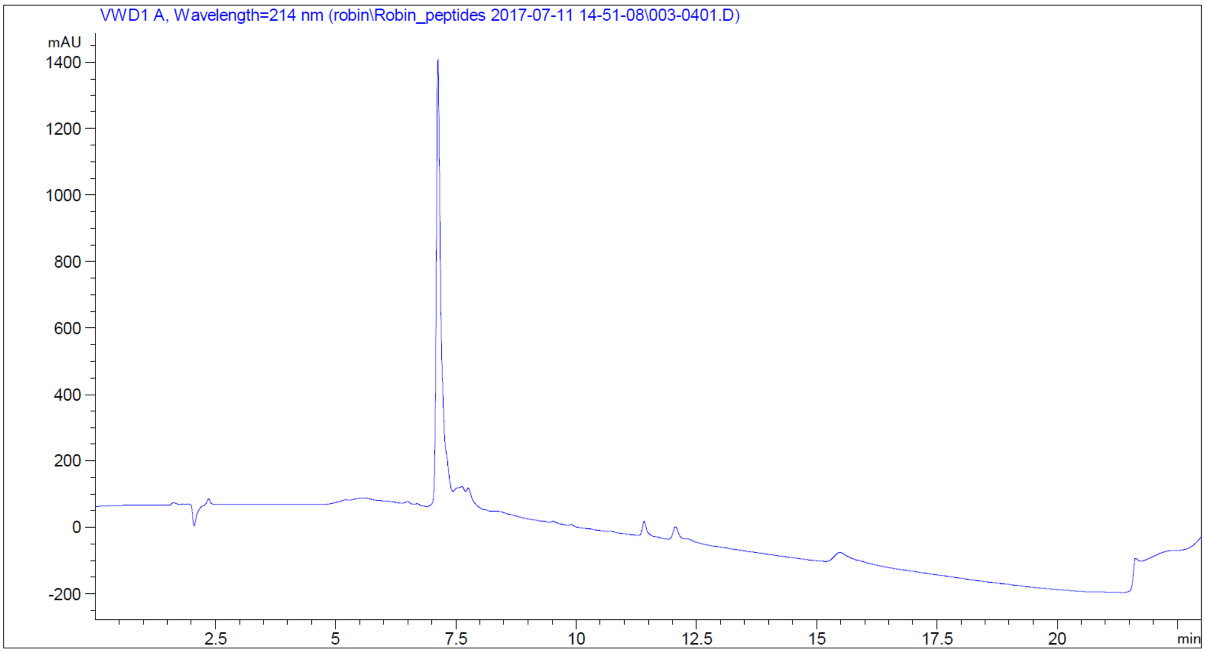

Analytical HPLC trace of purified AEYLR-RVRR-K_{16}; R_t 7.03 min (5% B: 0 to 2 min, 5% to 95% B: 2 to 19 min; 95% to 5% B: 19 to 20 min then 5% B 20 to 23 min λ = 214 nm).

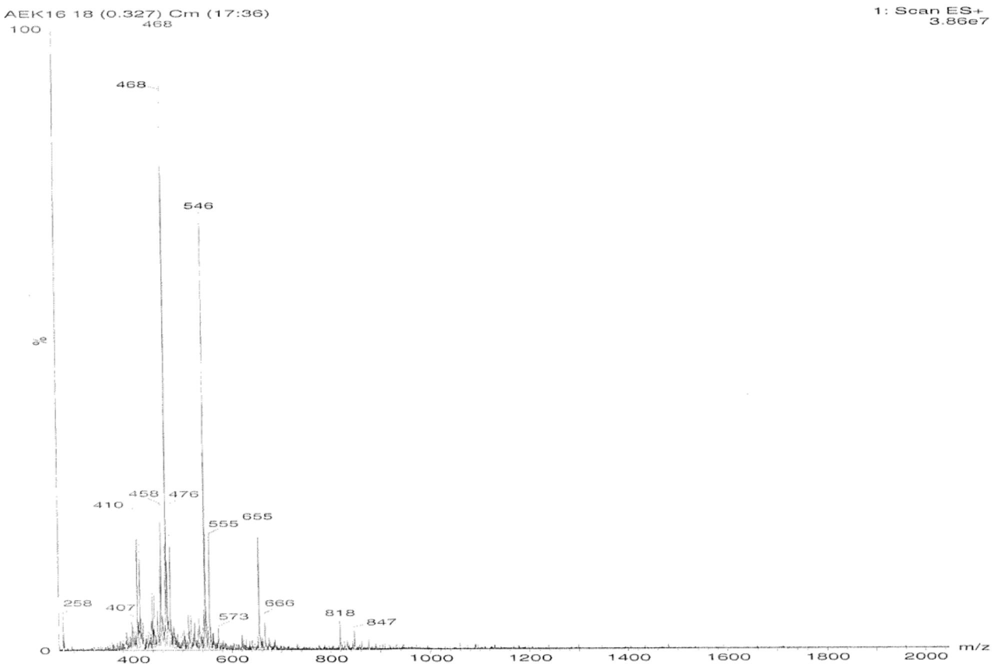

m/z Calculated Mass (ESI^+): 3269.19 $[M]^+$, 818.29 $[M+4H]^{4+}$, 654.83 $[M+5H]^{5+}$, 545.86 $[M+6H]^{6+}$, 468.02 $[M+7H]^{7+}$, 409.64 $[M+8H]^{8+}$, 364.24 $[M+9H]^{9+}$. Mass Found: 818 $[M+4H]^{4+}$, 655 $[M+5H]^{5+}$, 546 $[M+6H]^{6+}$, 468 $[M+7H]^{7+}$.

$CysLys_{16}$ 7

Preparative HPLC: conditions given in General Experimental, R_T 7.13 min

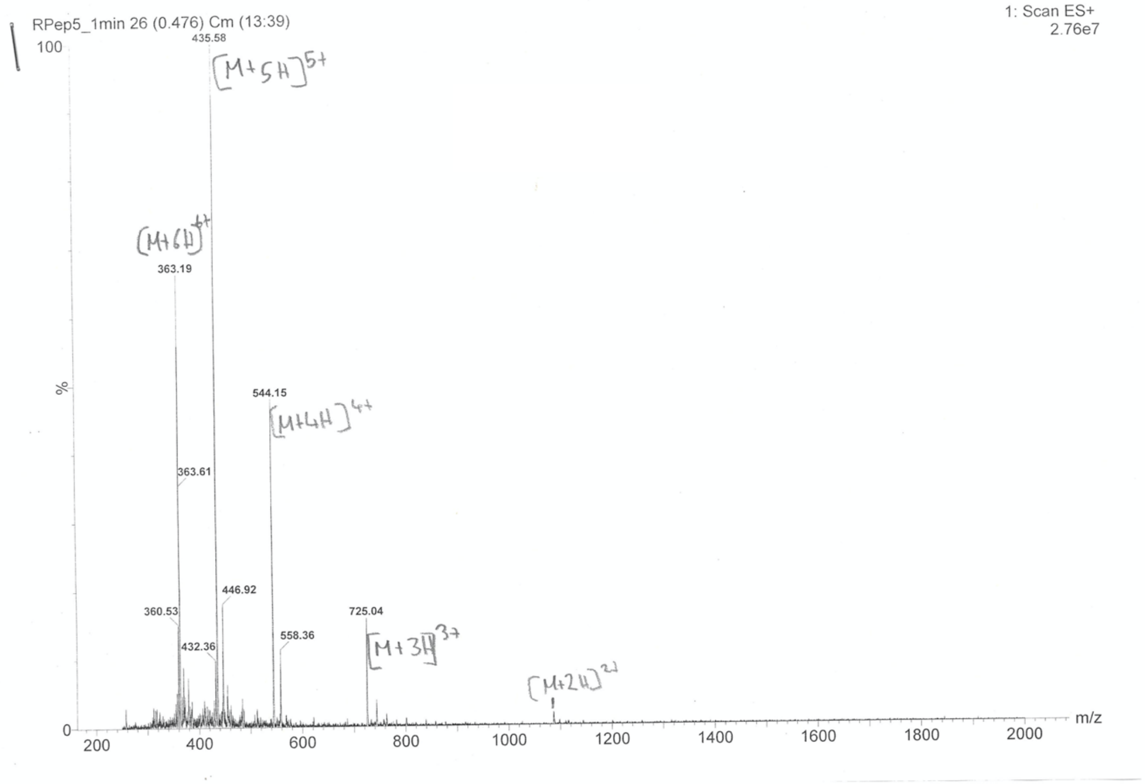

Compound 1

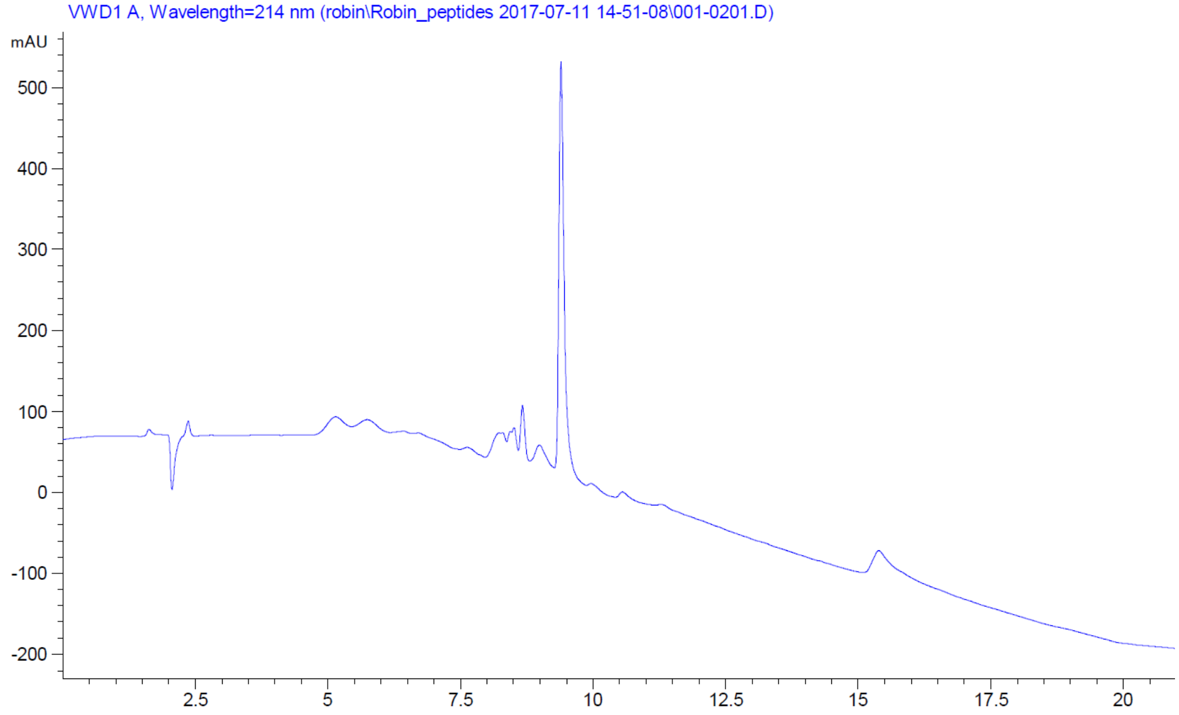

Analytical HPLC trace of purified **1**; R_t 9.49 min (5% B: 0 to 2 min, 5% to 95% B: 2 to 19 min; 95% to 5% B: 19 to 20 min then 5% B 20 to 23 min λ = 214 nm).

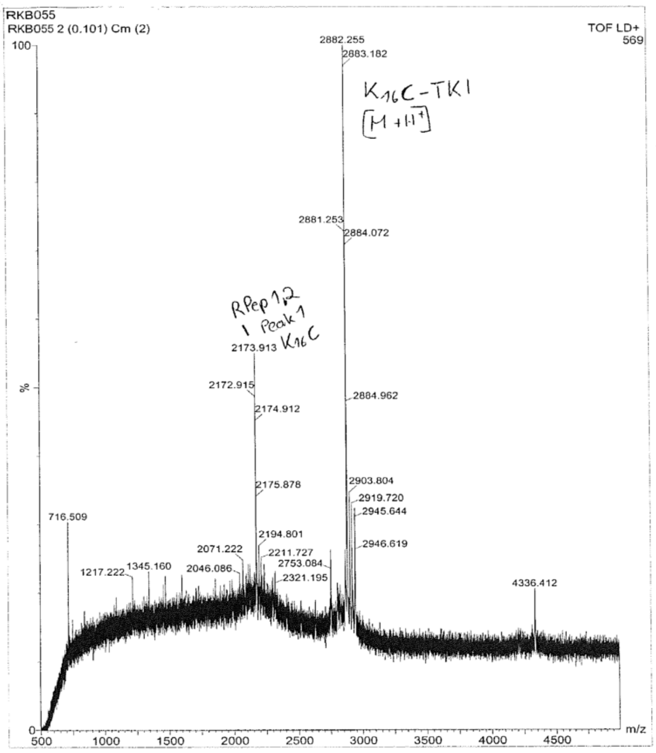

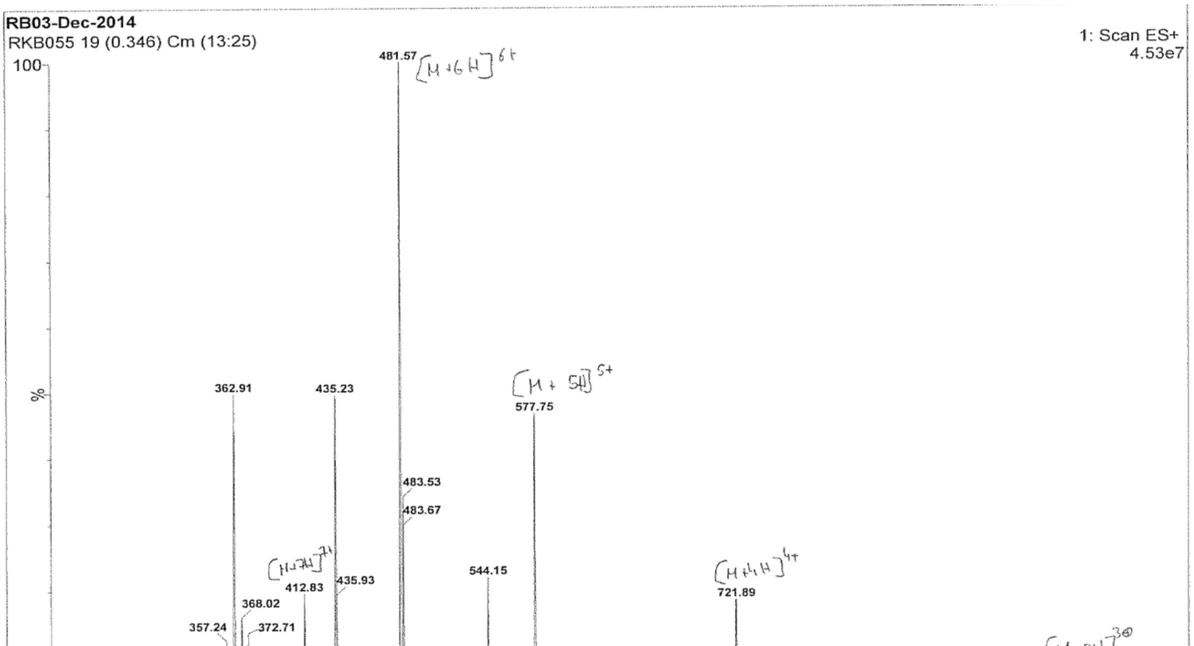

m/z Calculated Mass (ESI$^+$): 2880.67 $[M]^+$, 961.23 $[M+3H]^{3+}$, 721.18 $[M+4H]^{4+}$, 577.14 $[M+5H]^{5+}$, 481.12 $[M+6H]^{6+}$. Mass Found: 2881 $[M+H]^+$, 962 $[M+3H]^{3+}$, 721 $[M+4H]^{4+}$, 577 $[M+5H]^{5+}$, 481 $[M+6H]^{6+}$.

Compound 17

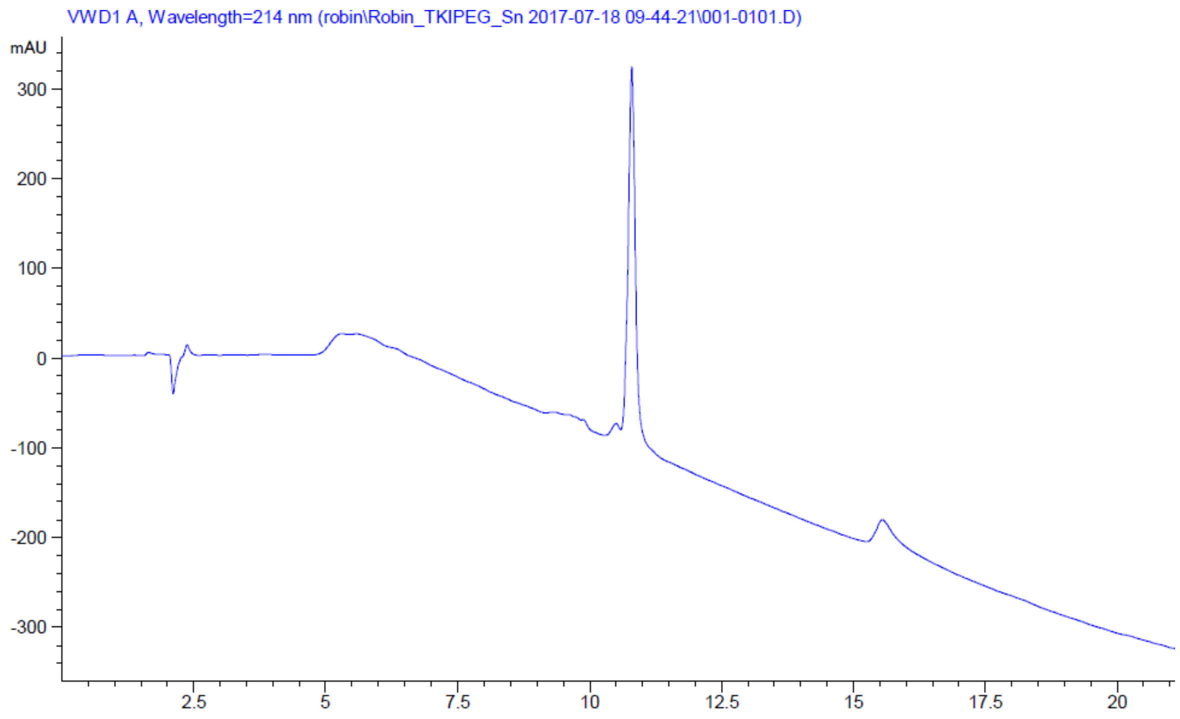

Analytical HPLC trace of purified **17**; R_t 11.11 min (5% B: 0 to 2 min, 5% to 95% B: 2 to 19 min; 95% to 5% B: 19 to 20 min then 5% B 20 to 23 min λ = 220 nm).

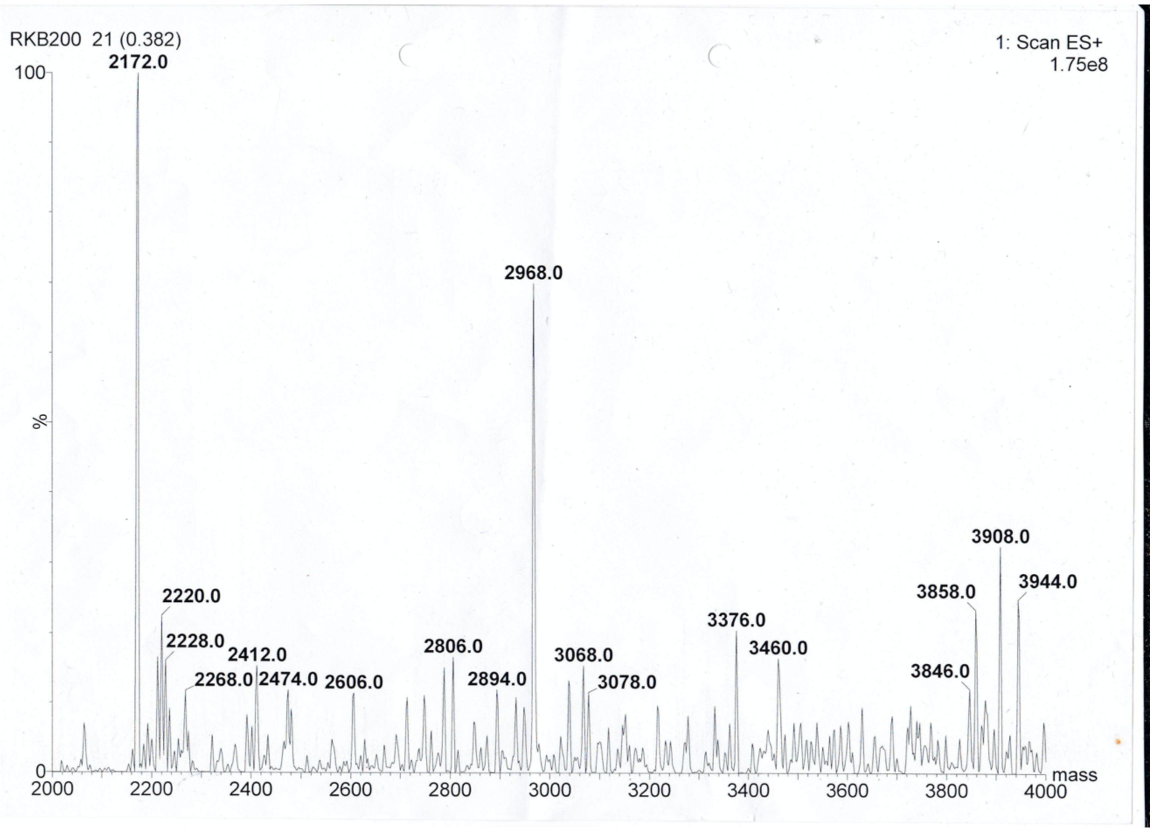

m/z Calculated Mass (ESI$^+$): 2967.75 $[M]^+$. Mass Found: 2968 $[M+H]^+$.

HPLC of Radiolabelled Compounds 8 and 9

Preparative HPLC profile of a [^{125}I]-**8** preparation (A) and quality control of isolated product (B).

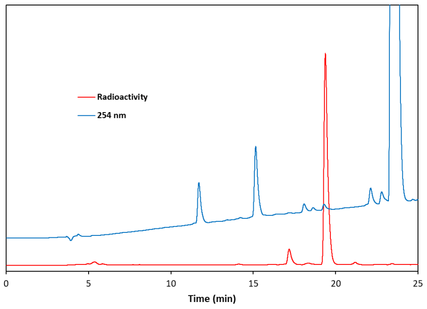

(A)
Column: Agilent Zorbax RP-18 (300 × 9.4 mm)
Flowrate: 3.00 mL/min

Gradient

Time (min)	H_2O + 0.1% TFA (%)	MeOH + 0.1% TFA (%)
0	60	40
25	10	90

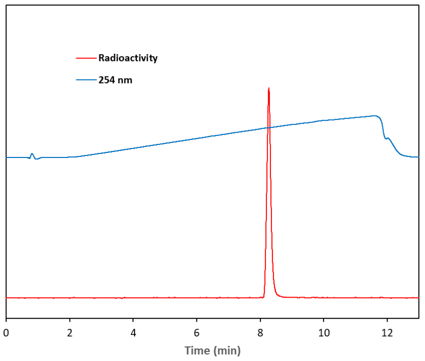

(B)
Column: Phenomenex Chromolith RP-18 (100 × 4.6 mm)
Flowrate: 3.00 mL/min

Gradient

Time (min)	H_2O + 0.1% TFA (%)	MeOH + 0.1% TFA (%)
0	90	10
10	10	90

Preparative HPLC profile of a [^{125}I]K_{16}-Cys-SMal-[PEG_3-IPQA] <u>**9**</u> preparation (A) and quality control of isolated product (B).

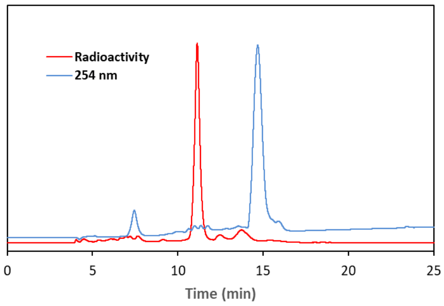

A) Column: Agilent Zorbax RP-18 (300 × 9.4 mm)
Flowrate: 3.00 mL/min

Gradient

Time (min)	H_2O + 0.1% TFA (%)	MeOH + 0.1% TFA (%)
0	60	40
25	10	90

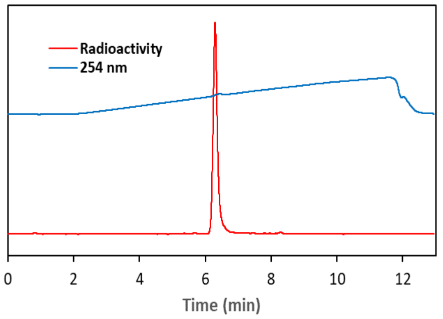

B) Column: Phenomenex Chromolith RP-18 (100 × 4.6 mm)
Flowrate: 3.00 mL/min

Gradient

Time (min)	H_2O + 0.1% TFA (%)	MeOH + 0.1% TFA (%)
0	90	10
10	10	90

Characterisation of lipopolyplexes

The lipopolyplexes were characterized using dynamic light scattering (DLS) and zeta potential measurements. Data were obtained using a Malvern Zetasizer Nano-ZS (Malvern, UK); aliquots of 25 – 50 µL were diluted to 1 mL in sterilized water and analysed in triplicate at 25 °C. For the *in vivo* experiments the lipopolyplexes were concentrated in order to provide a volume appropriate for injection, and the size and surface charge were characterised to ensure reproducibility.

Lipopolyplex Formulation	DLS /nm (PDI)	Zeta pot. /mV
CLA1	106.8 (0.213)	24.9 (+/- 2.1)
CLA2	136.0 (0.322)	34.8 (+/- 2.1)
CLA3	114.5 (0.273)	26.1 (+/- 3.7)
CLA4	132.9 (0.309)	31.3 (+/- 2.8)
NAE1	119.5 (0.225)	20.0 (+/- 2.7)
NAE2	130.2 (0.217)	27.2 (+/- 2.6)
NAE3	119.1 (0.246)	28.2 (+/- 1.4)
NAE4	115.1 (0.261)	18.5 (+/- 4.0)
NGE1	110.5 (0.256)	23.6 (+/- 2.9)
NGE2	122.2 (0.215)	31.6 (+/- 1.9)
NGE3	113.4 (0.225)	29.1 (+/- 1.1)
NGE4	112.0 (0.280)	22.6 (+/- 3.8)
F1LA1	135.2 (0.351)	11.7 (+/- 9.6)

Table S1. DLS and Zeta potential of liposome formulations.

DLS average of 2 values (except F1LA1 average of 3 values), zeta average of 3 values (+/- standard deviation).

References

1) G. W. Rewcastle, B. D. Palmer, A. J. Bridges, H. D. H. Showalter, L. Sun, J. Nelson, A. McMichael, A. J. Kraker, D. W. Fry and W. A. Denny, *J. Med. Chem.,* 1996, **39**, 918–928.
2) S. Dissoki, R. Eshet, H. Billauer and E. Mishani, *J. Labelled Compd. Radiopharm.*, 2009, **52**, 41–52.
3) A. Pal, J. A. Balatoni, U. Mukhopadhyay, K. Ogawa, C. Gonzalez-Lepera, A. Shavrin, A. Volgin, W. Tong, M. M. Alauddin and J. G. Gelovani, *Mol. Imaging Biol*. 2011, **13**, 853–861.
